# Combining MicroED and native mass spectrometry for structural discovery of enzyme–small molecule complexes

**DOI:** 10.1073/pnas.2503780122

**Published:** 2025-07-28

**Authors:** Niko W. Vlahakis, Cameron W. Flowers, Mengting Liu, Matthew P. Agdanowski, Samuel Johnson, Jacob A. Summers, Lian M. C. Jacobs, Catherine Keyser, Phoebe Russell, Samuel L. Rose, Julien Orlans, Nima Adhami, Yu Chen, Michael R. Sawaya, Shibom Basu, Daniele de Sanctis, Yu Chen, Soichi Wakatsuki, Hosea M. Nelson, Joseph A. Loo, Yi Tang, Jose A. Rodriguez

**Affiliations:** ^a^Department of Chemistry and Biochemistry, University of California, Los Angeles, CA 90095; ^b^Department of Energy Institute for Genomics and Proteomics, University of California, Los Angeles, CA 90095; ^c^Science and Technology Center on Real-Time Functional Imaging, NSF Science and Technology Center, University of California, Los Angeles, CA 90095; ^d^Department of Chemical and Biomolecular Engineering, University of California Los Angeles, Los Angeles, CA 90095; ^e^Division of Chemistry and Chemical Engineering, California Institute of Technology, Pasadena, CA 91125; ^f^Department of Structural Biology, Stanford University School of Medicine, Stanford, CA 94304; ^g^Biological Sciences Division, Stanford Linear Accelerator Center National Accelerator Laboratory, Menlo Park, CA 94025; ^h^Department of Molecular Medicine, Morsani College of Medicine, University of South Florida, Tampa, FL 33612; ^i^European Synchrotron Radiation Facility–The European Synchrotron, Grenoble 38000, France; ^j^European Molecular Biology Laboratory, Grenoble 38000, France

**Keywords:** cryoEM, MicroED, mass spectrometry, ligand, enzyme

## Abstract

With the goal of accelerating the discovery of small molecule–protein complexes, we leverage fast, low-dose, event-based electron counting microcrystal electron diffraction (MicroED) data collection and native mass spectrometry. This approach, which we term electron diffraction with native mass spectrometry (ED-MS), allows assignment of protein target structures bound to ligands with data obtained from crystal slurries soaked with mixtures of known inhibitors and crude biosynthetic reactions. This extends to libraries of printed ligands dispensed directly onto TEM grids for later soaking with microcrystal slurries, and complexes with noncovalent ligands. ED-MS resolves structures of the natural product, epoxide-based cysteine protease inhibitor E-64, and its biosynthetic analogs bound to the model cysteine protease, papain. It further identifies papain binding to its preferred natural products, by showing that two analogs of E-64 outcompete others in binding to papain crystals, and by detecting papain bound to E-64 and an analog from crude biosynthetic reactions, without purification. ED-MS also resolves binding of the CTX-M-14 β-lactamase, a target of active drug development, to the non-β-lactam inhibitor, avibactam, alone or in a cocktail of unrelated compounds. These results illustrate the utility of ED-MS for natural product ligand discovery and for structure-based screening of small molecule binders to macromolecular targets, promising utility for drug discovery.

## Significance

The accurate characterization of small molecule–protein interactions is critical for biomedical research. High-throughput structural methods, including X-ray crystallography, facilitate atomic views of such complexes and can rapidly inform drug development. By combining microcrystal electron diffraction with native mass spectrometry (ED-MS), we develop an approach to rapidly and accurately determine structures of biosynthetic or clinically relevant ligands bound to enzymes. ED-MS effectively captures the natural product, E-64, and its biosynthetic variants, bound to their target protease, papain. It also resolves the inhibition interaction of avibactam with an M14 β-lactamase. These complex structures can even be extracted from microcrystal slurries soaked with ligand mixtures and crude biosynthetic reactions, demonstrating the broad utility of ED-MS for research, drug discovery, and medicinal chemistry.

Several methods can inform on ligand binding to proteins. Techniques such as isothermal titration calorimetry or surface plasmon resonance offer both confirmation that a ligand is binding a protein of interest, and elucidate details about binding kinetics and thermodynamics of the complex (1, 2). Mass spectrometry can now not only unambiguously confirm the occurrence of protein–ligand binding interactions, but also precisely identify binding sites (3, 4). Likewise, NMR has been successfully leveraged for the detection of ligand-binding events in chemical fragment screens (5, 6), and can additionally offer three-dimensional (3D) structures of ligand binding sites for small protein targets (7). However, when detailed 3D atomic information about a target protein is needed, such as a case where conformational changes of the target in response to ligand binding are of interest, or where binding sites are unknown, crystallographic insights remain the gold standard (8).

The development of new methods to interrogate minuscule protein crystals has furthered the frontier of protein–ligand complex characterization. Laser and synchrotron-based X-ray sources continue to develop, delivering high-resolution structures from assemblies of shrinkingly fewer repeat units (9). This is facilitated in part by advances in serial crystallography at both synchrotrons and X-ray free electron lasers, which offer atomic resolution structures from slurries of nanocrystals (10–13). Complementing these efforts, microcrystal electron diffraction (MicroED), or 3D electron diffraction, yields high-resolution crystal structures from necessarily thin (<500 nm) crystals, often capitalizing on a target’s proclivity to form small crystals (14–16), and enabling more rapid ligand diffusion and binding than is possible with large, X-ray diffraction (XRD)-suitable crystals (17, 18).

The advantage of efficient ligand soaking in MicroED makes possible the high-throughput structural evaluation of ligand binding to proteins, especially where a binding site on the target protein is known. As such, a method that enables screening multiple candidate ligands against crystals of the protein of interest simultaneously, pinpointing whether one or more has bound, would be an advantageous addition to the structure-based drug discovery toolkit. This is illustrated by fragment-binding screens, where small molecule fragments representing a range of chemical space are simultaneously soaked into crystals of interest, typically prior to XRD (8, 19, 20). However, that approach is challenged when the bound ligand is not fully resolved or where resolution is insufficient for unambiguous atomic assignment (21, 22). If the identity of a potential ligand is not known beforehand, a secondary validation method, such as mass spectrometry, can be useful (23).

Native mass spectrometry (nMS) is ideal for this role since it allows for the observations of protein complexes and protein–ligand interactions in their near-native states, preserving noncovalent interactions (24). Nano electrospray ionization (ESI) facilitates the desolvation and ionization of proteins in a volatile buffer, and its coupling with mass spectrometry is capable of identifying species in complex mixtures with low sample volume (25). In fact, nMS has played a crucial role in evaluating small molecule libraries for downstream applications in XRD soaking experiments (26), highlighting its potential for more high-throughput analysis. The application of nMS directly to samples used for MicroED (ED-MS), therefore, offers the potential for accurate structures of protein–ligand crystals obtained from soaks with compound mixtures.

**Fig. 1.**
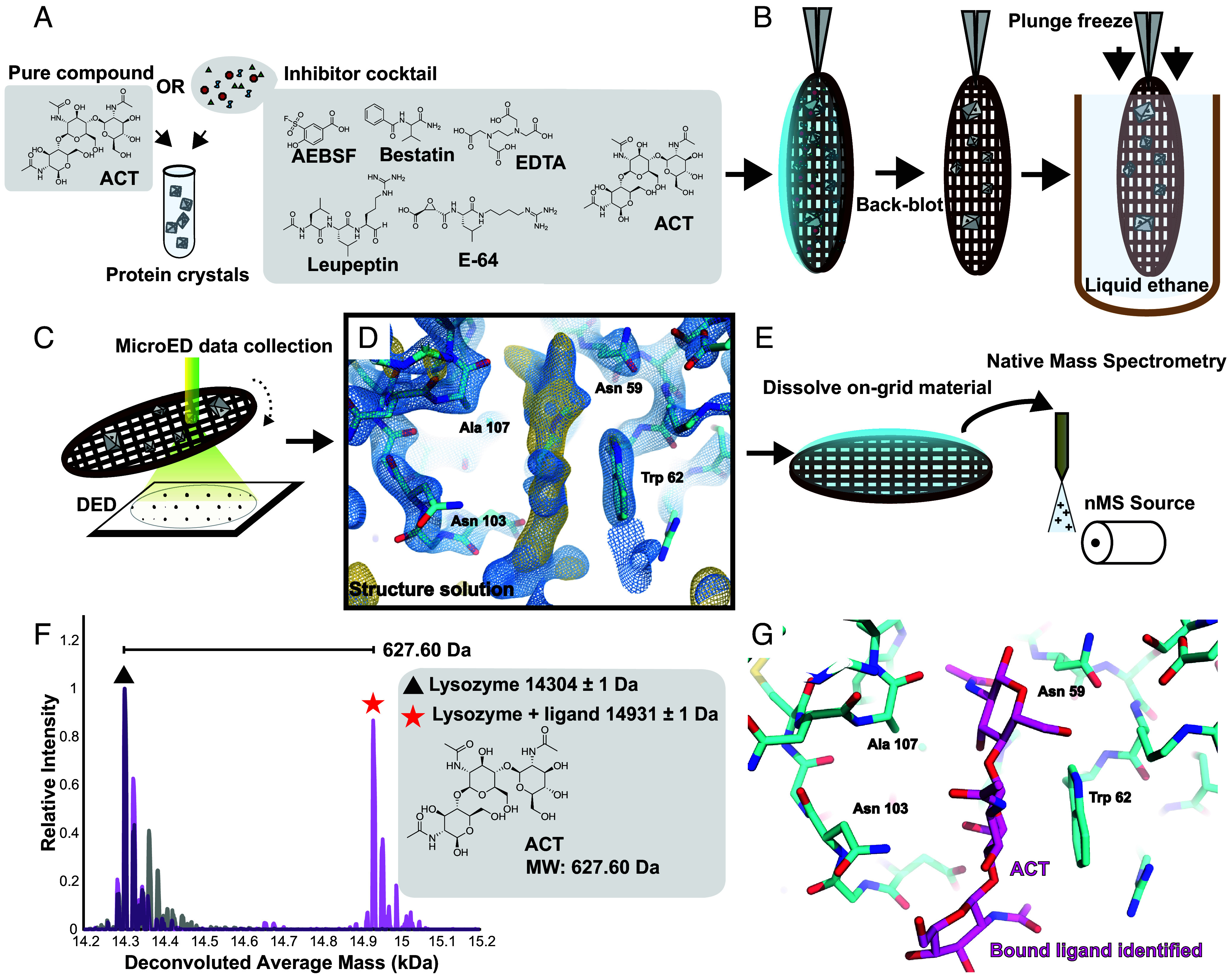
Scheme of ED-MS for resolving bound ligands in protein microcrystals. A slurry of protein microcrystals is soaked with a mixture of potential ligands; the process is demonstrated with lysozyme and its ligand, ACT (*A*). This microcrystalline suspension is loaded on a holey carbon TEM grid, blotted from the back to remove excess solvent, and plunge-frozen in liquid ethane (*B*). Low-dose MicroED data are acquired using a direct electron detector (*C*), yielding a structure solution of the microcrystals where unsatisfied density in the potential map can be identified as probable ligands (Blue mesh: 2F_o_ − F_c_ map at 1.5σ level; gold mesh: F_o_ − F_c_ map at the 3σ level) (*D*). The grid is then recovered from the TEM, and the on-grid crystals dissolved in a volatile solvent, prior to nMS analysis (*E*). An nMS spectrum of the complex (magenta) compared to the apo-protein (gray) indicates mass shifts for potential bound ligands, such as that of the 627.60 Da ACT bound to lysozyme shown in this example (*F*). This ligand is then modeled into the residual density in the MicroED structure and fully refined, resulting in an atomic structure of the complex (*G*).

Here, we demonstrate the utility of the combined ED-MS approach for resolving protein–ligand complexes that are generated by soaking protein microcrystal slurries with either a pure compound, or a cocktail of potential ligands (Fig. 1). We demonstrate this approach with the model protein lysozyme bound to its noncovalent ligand N,N′,N″-triacetylchitotriose (ACT), then use the cysteine protease papain as a scaffold on which to bind a panel of both known and potential inhibitors, including the epoxide-based covalent cysteine protease inhibitor E-64 (27), its commercially available chemical analogs (28), and analogs generated by biosynthetic reaction (29). We determine structures of papain soaked with mixtures of these potential inhibitors, and perform nMS directly on TEM grid-adsorbed crystals following diffraction data collection to identify the exact masses of bound ligands present. Last, we extend the ED-MS workflow to interrogate crystals of the β-lactamase enzyme, CTX-M-14, which imparts resistance to β-lactam-containing antibiotics by hydrolyzing their four-membered ring structural motif (30–32). In particular, we assessed the structure of β-lactamase in complex with avibactam, which acylates the active serine residue without being hydrolyzed by the enzyme (33). Overall, our results demonstrate that structures of protein–ligand complexes can be obtained directly from microcrystal slurries by ED-MS, with unambiguous confirmation of the identity of the bound ligands. The approach promises to structurally illuminate the binding of proteins to natural products and active drug-like molecules present in cocktails or reaction mixtures.

## Results

### ED-MS As a Facile Approach for Ligand–Protein Structure Determination.

The overall ED-MS workflow involves the soaking of microcrystal slurries with either a single, pure small molecule binder, or a cocktail of potential ligands that includes a binder molecule (Fig. 1*A*), preceding TEM grid preparation and sample vitrification (Fig. 1*B*). Using samples prepared by this strategy, we acquire continuous rotation MicroED tilt series at 200 kV using a direct electron detector (Fig. 1*C*) yielding a structure phased by molecular replacement, where unassigned residual density may suggest the presence of one or more bound ligands (Fig. 1*D*). The TEM grid is then recovered, the on-grid material dissolved, and analyzed directly by nMS (Fig. 1*E*), producing a spectrum where mass shifts confirm the identity of bound species (Fig. 1*F*). The identified ligand can then be modeled into the residual density of the MicroED structure and refined (Fig. 1*G*).

To assess the utility of ED-MS for characterizing protein–ligand complexes, we set out to determine the structure of the venerable protein standard, lysozyme, bound to its noncovalent ligand, ACT. Macroscopic lysozyme crystals (*SI Appendix*, Fig. S2*A*) verified to diffract well by single-crystal XRD (*SI Appendix*, Fig. S2*B*) were crushed to form microcrystalline slurries suitable for producing high-resolution electron diffraction (*SI Appendix*, Fig. S1 *A* and *B*). Compared to structures of the unliganded protein (*SI Appendix*, Fig. S1*C* and Table S1), a MicroED structure at 2.3 Å resolution of lysozyme cocrystallized with ACT showed prominent density indicating the presence of the anticipated ligand (*SI Appendix*, Fig. S1*F* and Table S1). Likewise, relative to the nMS spectrum acquired from the grid of apo-form lysozyme crystals (*SI Appendix*, Fig. S1*D*), the spectrum recorded from the grid of cocrystals showed a clear 627 Da shift relative to the apo-form mass indicating bound ACT. Curiously, this spectrum also contained a peak at 15,135 Da, 204 Da heavier than the mass of the bound state, suggesting the presence of an additional binder to lysozyme we did not observe in our structure (*SI Appendix*, Fig. S1*G*). Nevertheless, this result indicated that noncovalent ligands detectable by MicroED can be preserved during sample preparation for nMS, and captured in the measured spectrum. To assess the robustness of this procedure in the more complicated case of a cocktail-soaked microcrystalline slurry, we performed a similar experiment, this time mixing ACT with a concentrated protease inhibitor cocktail, and soaking a microcrystalline slurry of apo-form lysozyme for 10 min prior to plunge freezing on TEM grids. The MicroED structure determined from these crystals, at 2.4 Å resolution, showed ACT density matching that seen in the cocrystal structure (*SI Appendix*, Fig. S1*J* and Table S1), and the corresponding nMS spectrum once again confirmed the 627 Da mass shift for the ligand, this time without any evidence of additional ligands from the mixture (*SI Appendix*, Fig. S1*K*). Density for ACT in each MicroED experiment was comparable to that observed from single-crystal XRD structures of lysozyme cocrystallized or soaked with either pure ACT or a cocktail containing ACT (*SI Appendix*, Fig. S2 *D*–*H* and Table S2).

### MicroED Structures of Papain Bound to the Cysteine Protease Inhibitor Natural Product, E-64.

**Fig. 2.**
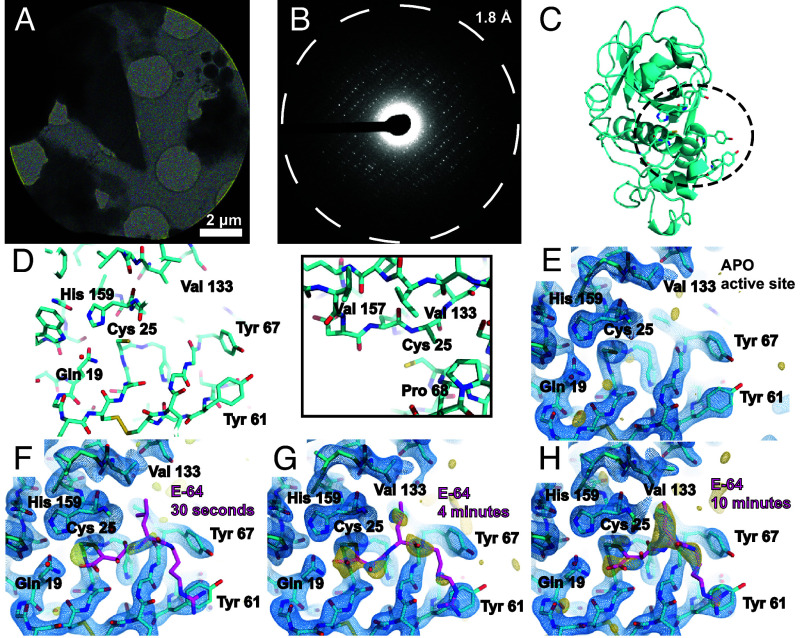
MicroED structures of papain and E-64–papain complexes. TEM image of a thin frozen-hydrated papain microcrystalline fragment (*A*), which yields high resolution electron diffraction when illuminated with a low-flux parallel beam (*B*). Atomic model of the cysteine protease papain, determined by MicroED to 2.5 Å resolution with secondary structure and key active-site residue side chains rendered, with the dashed circle highlighting the active site (*C*). View of papain’s active site from the same model, with key residues labeled. Covalent inhibitors bind in this pocket to cysteine 25, and a hydrophobic pocket (view in *Inset*) constituting valine 133, valine 157, and proline 68 accommodates aliphatic moieties of potential binders (*D*). Structure of the apo-form of papain at 2.5 Å resolution determined by MicroED from crystals such as the one shown in panel *A*, where the active site is viewed (*E*). MicroED structures of the papain active site at 2.5 Å resolution from microcrystals soaked with a concentrated solution of E-64 for 30 s (*F*), 4 min (*G*), and 10 min (*H*). Blue mesh indicates the 2F_o_ − F_c_ map at 1.5σ level following modeling of the ligand (magenta coordinates), and gold mesh indicating the F_o_ − F_c_ map at the 3σ level that was present prior to modeling a ligand. 10 min of soaking is sufficient for high (>80%) occupancy to be achieved.

We next interrogated ligand interactions of the cysteine protease papain, as isolated from papaya latex (34). Papain crystals (Fig. 2*A*) were readily grown and cocrystallized or soaked with target ligands for MicroED analysis. Crystals that diffracted to high resolution by XRD (*SI Appendix*, Fig. S3 *D* and *E*) were crushed by repeated pipetting in their crystallization solution and frozen on cryo-electron microscopy (cryoEM) grids to yield a population of well-diffracting nanocrystalline fragments (Fig. 2 *A* and *B*). A 2.5 Å resolution MicroED structure of papain was phased by molecular replacement (34) (Fig. 2 *C*–*E* and *SI Appendix*, Table S3).

We validated that the known epoxide-based cysteine protease inhibitor E-64 covalently bound papain (*SI Appendix*, Fig. S4 *A* and *B*) and could be detected by MicroED when soaked into papain microcrystal slurries. Given the potential speed advantage in ligand soaking with the thin crystals used for MicroED, we evaluated the soaking time required to observe sufficient occupancy of E-64 in the papain active site. Papain microcrystals frozen on cryoEM grids following either 30 s, 4 min, or 10 min of soaking in 2.5 mM E-64 were interrogated by MicroED, each yielding a structure with a clear view of the binding pocket surrounding critical residue cysteine 25 (Cys25) at 2.5 Å resolution (Fig. 2 *F*–*H* and *SI Appendix*, Table S4). While a weak trace of E-64 density was observed following 30 s of soaking (Fig. 2*F*), both the 4- and 10-min structures showed sufficient density to model E-64 and carry out refinement (Fig. 2 *G* and *H*). In particular, the refined occupancy of E-64 atoms in the structure from crystals soaked for 10 min exceeded 75%, with ligand B-factors similar in value to the average refined B-factor of papain atoms (*SI Appendix*, Table S4). This confirmed the 10-min soaking time as sufficient to visualize strong covalent binding by E-64 (*SI Appendix*, Table S5). The soaking efficiency noted in microcrystals exceeded that of equivalently soaked macroscopic crystals needed for single-crystal XRD (*SI Appendix*, Fig. S5 and Table S6).

Although achieving high completeness MicroED data can require the merging of partial datasets from multiple crystals, we noted that the clearest density in papain’s active site in F_o_ − F_c_ ligand omit maps was often achieved from unmerged or minimally merged datasets, provided completeness exceeded approximately 85%. This is likely due to variable ligand occupancy between different microcrystals on the same TEM grid (*SI Appendix*, Fig. S6 *A*–*C*). As such, the ideal data collection modality required wedges of data that are as large as possible, to be quickly acquired on individual papain crystals prior to much accumulation of radiation damage. This was uniquely facilitated by the use of a fast direct electron detector (DE Apollo) performing event based electron counting (35, 36). Leveraging the DE Apollo camera for fast, low-flux MicroED data collection (37), we were able to routinely acquire structure-worthy tilt series delivering no more than 6 e^−^/Å^2^ total fluence per crystal.

### Unambiguous Determination of Papain–E-64-Like Natural Product Complexes Enabled by ED-MS.

**Fig. 3.**
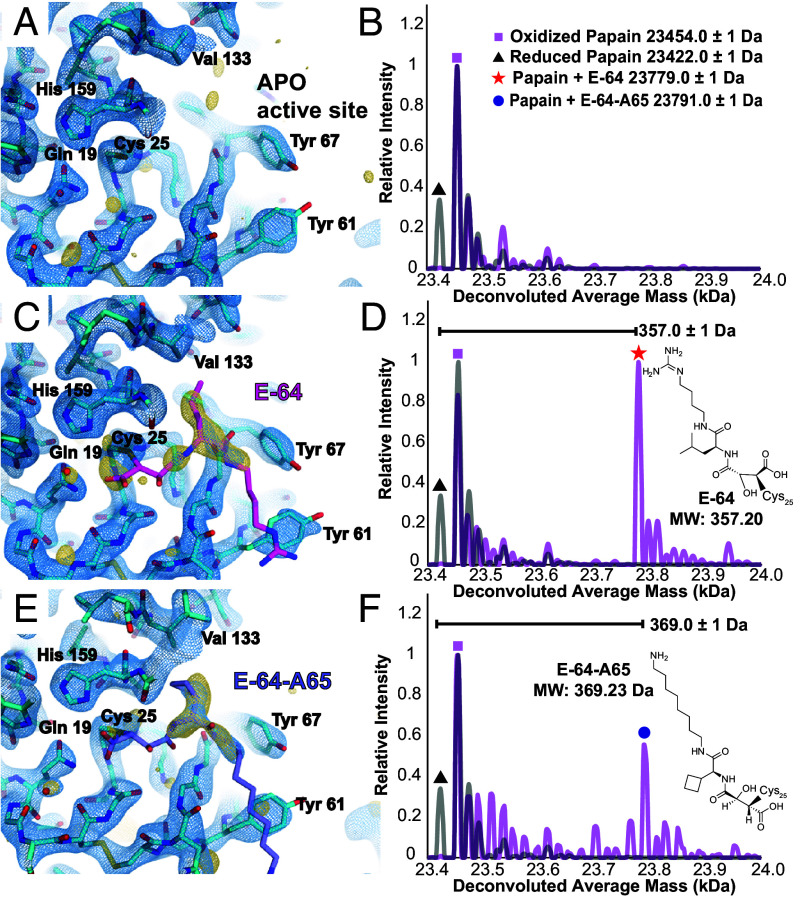
ED-MS of papain complexed with E-64 and a biosynthetic analog. Structure of the apo-form of papain at 2.5 Å resolution determined by MicroED, where the active site is viewed (*A*), and nMS spectrum of the same material harvested and dissolved from the cryoEM grid (*B*). Structure of the papain–E-64 cocrystal complex at 2.3 Å resolution determined by MicroED (*C*), and corresponding nMS spectrum confirming presence of the papain–E-64 complex on the grid (*D*). Structure of the papain–E-64-A65 cocrystal complex at 2.5 Å resolution determined by MicroED (*E*), and corresponding nMS spectrum confirming presence of the papain–E-64-A65 complex on the grid (*F*). Blue mesh indicates the 2F_o_ − F_c_ map at 1.5σ level following modeling of the ligand, and gold mesh indicates the F_o_ − F_c_ map at the 3σ level that was present prior to modeling a ligand. In *B*, *D* and *F*, grid-derived nMS spectra (magenta) are superimposed on the spectrum acquired from the apo-protein (gray).

We anticipated MicroED would be a useful tool for ligand discovery from natural product libraries. We therefore proceeded to determine the structure of papain microcrystals cocrystallized in the presence of an E-64 analog recently discovered by a combinatorial biosynthesis platform (29), which we refer to as E-64-A65 (*SI Appendix*, Fig. S4 *C* and *D*). A 2.5 Å resolution structure of the papain–E-64-A65 cocrystal complex showed prominent density in the active site compared to the respective apo site, confirming the bound biosynthetic compound (Fig. 3 *A* and *E* and *SI Appendix*, Table S3). We noted in doing so that although MicroED was able to rapidly deliver a structure of the bound ligand complex, the ligand density was not always sufficient to disambiguate between structurally similar binders such as E-64-A65 and its parent, E-64. This is evident by comparing the E-64-A65-bound structure to a high-occupancy structure of papain cocrystallized with E-64 and determined to 2.3 Å resolution (Fig. 3*C* and *SI Appendix*, Table S3). This scenario would be common during screening of crystal binding to highly similar ligands, or to mixtures or cocktails of similar ligands, as might be performed in high-throughput drug discovery efforts, and presented an opportunity to apply ED-MS to resolve the ambiguity.

Mass spectra acquired from apo papain crystals harvested from a TEM grid showed masses corresponding to the protein with both a doubly oxidized, sulfinic acid form (+32 Da) of its catalytic residue Cys25, and a reduced form of the protein (Fig. 3*B*). The assignment of this oxidation state is substantiated by its resistance to reduction with dithiothreitol (DTT) or tris(2-carboxyethyl)phospine (TCEP), unlike other +32 Da modifications such as persulfides, which would be reduced under these conditions (*SI Appendix*, Fig. S7). By comparison, mass spectra acquired from on-grid material of both papain–E-64 and papain–E-64-A65 cocrystals offered unambiguous confirmation of the identity of each bound species. A 357 Da and 369 Da mass-shifted species relative to the reduced form of papain, in the mass spectrum acquired from papain–E-64 and papain–E-64-A65 samples respectively, dominated in each, matching the expected molecular weights of the ligands: E-64 (357.20 Da) and E-64-A65 (369.23 Da) (Fig. 3 *D* and *F* and *SI Appendix*, Table S7). These results therefore validated ED-MS as an effective approach for measuring the mass of bound compounds within microcrystals used for structure determination, informing on both the identity and bound structure of an unknown ligand.

### Structure of Papain–E-64 Captured by ED-MS from Microcrystal Slurry Soaked with an Inhibitor Cocktail.

**Fig. 4.**
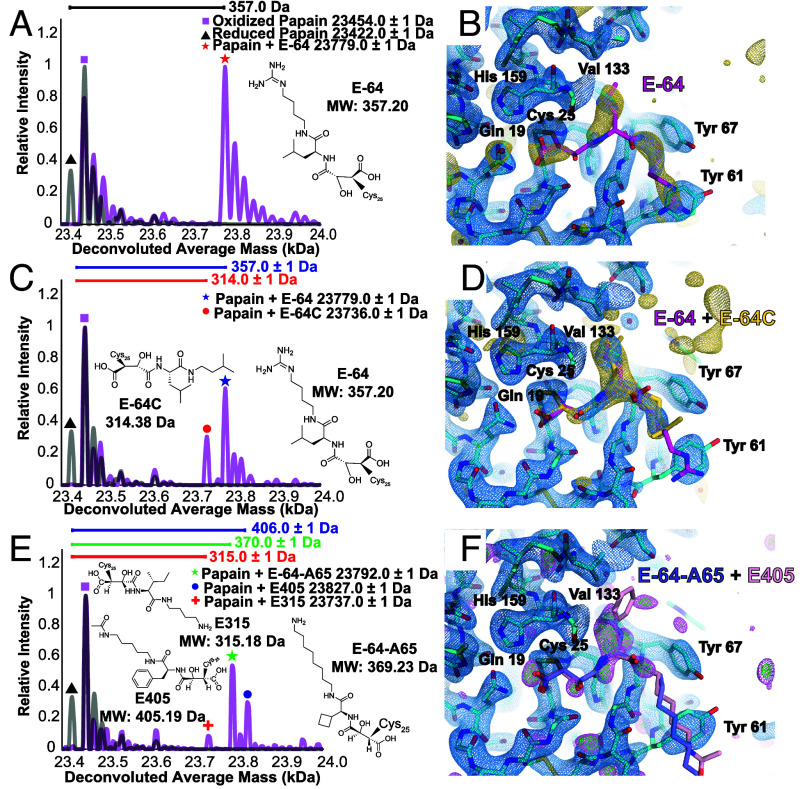
ED-MS reveals complex structures from papain microcrystals soaked with cocktails of potential ligands. nMS spectrum from on-grid papain microcrystals soaked with inhibitor cocktail containing AEBSF, bestatin, EDTA, leupeptin, aprotinin, and E-64, revealing presence of the papain–E-64 complex and no other bound species (*A*). MicroED structure of papain with E-64 modeled into its residual active-site density; an 2.5σ F_o_ − F_c_ ligand-omit map is overlaid in gold (*B*). nMS spectrum from on-grid papain microcrystals soaked with an equimolar mixture of the high-affinity inhibitors E-64, E-64C, and E-64D, showing presence of the papain–E-64 and papain–E-64C complexes (*C*), and active-site view of the MicroED structure with E-64 and E-64C modeled as alternate conformations and the 2.5σ F_o_ − F_c_ ligand-omit map overlaid in gold (*D*). nMS spectrum from on-grid papain microcrystals soaked with an equimolar mixture of biosynthetic E-64 analogs E-64-A65, E315, E371, and E405, showing presence of primarily the papain–E-64-A65 and papain–E405 complexes simultaneously, with trace amounts of the papain–E315 complex (*E*). Active-site view of the corresponding MicroED structure with no ligands modeled in the binding pocket, but with E-64-A65 and E405 in their conformations determined by XRD overlaid onto the model (F_o_ − F_c_ map displayed at 2.5σ level in magenta and 3σ level in green) (*F*). In *A*, *C* and *E*, grid-derived nMS spectra (magenta) are superimposed on the spectrum acquired from the apo-protein (gray).

Having validated that we could quickly soak ligands into papain microcrystals, visualize them by MicroED, and confirm their identities by nMS, we verified the utility of this workflow for higher-throughput ligand discovery efforts. In particular, we aimed to test whether the same information about ligand presence, conformation, and identity could be extracted by ED-MS for papain crystals soaked with a mixture of potential ligands. To evaluate this, we soaked papain microcrystals with a commercially available protease inhibitor cocktail containing E-64, AEBSF, leupeptin, aprotinin, bestatin, and ethylenediaminetetraacetic acid (EDTA) at varying relative concentrations (*SI Appendix*, Fig. S8). Of these components, both E-64 and leupeptin are known ligands of papain (27, 38). Cocktail-soaked papain microcrystals were then deposited and plunge-frozen on TEM grids and interrogated by ED-MS. A 2.5 Å resolution MicroED structure revealed unsatisfied active-site density in the absence of a modeled ligand. nMS analysis confirmed that the papain–E-64 complex was the only bound species dominantly present in the TEM grid-adsorbed crystals (Fig. 4*A*). Therefore, E-64 was subsequently modeled into the unassigned density in the final steps of structure refinement (Fig. 4*B* and *SI Appendix*, Table S8).

Even at the resolution afforded by single-crystal XRD, distinguishing E-64 from leupeptin based solely on the appearance of active site density in a 1.5 Å structure from macroscopic crystals soaked with the same cocktail was nontrivial (*SI Appendix*, Table S9) without the additional information afforded by nMS (*SI Appendix*, Fig. S9). Notably, nMS confirmed that leupeptin does indeed bind papain in solution and in microcrystals (*SI Appendix*, Fig. S10 *B*−*D*). Likewise, when crystallized in the presence of a molar excess of leupeptin, single-crystal XRD shows unambiguous density for bound leupeptin in the papain active site (*SI Appendix*, Fig. S10*A* and Table S10). Therefore, the results of the inhibitor cocktail soaking experiment indicate that, if multiple binders are present in the soaking mixture, the higher affinity binder may outcompete the others and be unambiguously identified by ED-MS at the short time and length scales evaluated.

### ED-MS Applied to Papain Bound to Mixtures of Its High Affinity Ligands, E-64, E-64C, and E-64D.

We set out to test the limits of structural information achievable by ED-MS for crystals soaked with mixtures of multiple high-affinity binders. In addition to E-64, papain is known to bind its commercially available analogs E-64C and E-64D (28), and their complexes have known structures (29). We expected ED-MS might reveal whether one of these known high-affinity ligands could outcompete the others when mixed in equimolar quantity and soaked into papain microcrystals. A 2.5 Å resolution MicroED structure was determined from mixture-soaked papain crystals, where unsatisfied density in the active site was detected that could not be confidently assigned to any one of the three structurally similar candidate compounds. nMS of the same crystals revealed the mass shifts corresponding to the E-64 and E-64C bound states of papain were both present in the mixture (Fig. 4*C*). The active site of the MicroED structure was therefore modeled as an ensemble of the papain–E-64 and papain–E-64C bound states (Fig. 4*D* and *SI Appendix*, Table S6). Even in higher-resolution XRD structures, determined either from serial synchrotron XRD (SSX) of papain microcrystals soaked with the same mixture (*SI Appendix*, Fig. S11 and Table S11), or from single-crystal XRD of a large crystal soaked with the mixture for an extended 20 h time (*SI Appendix*, Fig. S12*A*), the residual density in the active site only clearly showed the structural fragment common to all three ligands (*SI Appendix*, Table S9).

Curiously, when interrogating papain–E-64C and papain–E-64D cocrystal complexes separately by ED-MS (*SI Appendix*, Fig. S13 and Table S12), nMS revealed in each case that only the bound species corresponding to E-64C, indicated by a 314 Da mass shift from the reduced apo-form papain species, was present (*SI Appendix*, Fig. S13 *C* and *F*). To confirm the veracity of the stock E-64D, we determined its structure directly from the microcrystalline powder used for the ligand soaking experiment, and noted the refined 0.8 Å resolution structure (*SI Appendix*, Fig. S14 *E*–*G* and Table S13) matched the expected structure of E-64D, with a molecular weight of 342 Da. Likewise, liquid chromatography / mass spectrometry (LC-MS) of the E-64D dissolved in 66% methanol solution revealed the 342 Da mass, distinct from the 314 Da mass of E-64C (*SI Appendix*, Fig. S14 *A*–*D*). The only structural difference between E-64C and E-64D is the presence of an ester, rather than a terminal carboxylic acid, bound to the epoxy warhead. We therefore concluded that the ester of E-64D is likely hydrolyzed specifically in the course of its covalent attachment to the papain active site cysteine, effectively converting into E-64C, as observed in the crystal structure. This hypothesis is consistent with previous work studying the increased likelihood of ester hydrolysis in the presence of neighboring sulfides (39).

### ED-MS of Papain Bound to On-Grid Arrayed Ligand Sets.

Leveraging arrayED technology (40), we interrogated papain crystals applied to grids on which one or more target ligands had been predeposited (*SI Appendix*, Fig. S15). We reasoned that the printing of potential ligands on grids using microarrayer devices would streamline high-throughput soaking and ED-MS efforts, in cases where the compounds of study can be allowed to dry without degrading. A microarrayer (40) was used to deposit 250 to 350 picoliter droplets of E-64 onto TEM grids, which were then dried and stored until ready for soaking (*SI Appendix*, Fig. S15*A*). A slurry of papain microcrystals was then applied to the arrayED grids and incubated for 5 min before freezing and ED-MS analysis (*SI Appendix*, Fig. S15*B*). Data collected from these grids yielded a 2.8 Å structure of papain complexed with E-64 (*SI Appendix*, Fig. S15 *C*–*E* and Table S14), and nMS on the same grid revealed distinct presence of the papain–E-64 complex on the sample (*SI Appendix*, Fig. S15*F*). This nMS measurement additionally confirmed that the printed E-64 could be dried on the grid, later to be resuspended to bind papain.

### ED-MS Detects High Affinity Papain–Inhibitor Complexes from a Cocktail of Biosynthetic E-64 Derivatives.

To test the utility of ED-MS for drug discovery efforts from mixtures of natural product libraries, we soaked papain microcrystals with an equimolar mixture of multiple biosynthetic E-64 analogs. This collection included E-64-A65, as well as a series of similar compounds generated by substituting different amino acids and amine groups as precursors for the biosynthetic reaction used to produce E-64 (29). These compounds, with molecular weights of 315, 371, and 405 Da, are referred to as E315, E371, and E405 (*SI Appendix*, Fig. S16). While nMS of on-grid crystals soaked in a mix of these compounds found clear signal for both papain–E-64-A65 and papain–E405 complexes, and trace signals for the papain–E315 complex (Fig. 4*E*), only weak density could be observed in the active site of a 2.4 Å MicroED structure of the mixture (*SI Appendix*, Table S8). Nonetheless, omit difference maps showed residual active site density that partially matched the more ordered fragments of E-64-A65 and E405 (Fig. 4*F*). Repeating the same experiment, but excluding E-64-A65 from the cocktail, we measured masses for papain complexes with each of the three other natural products, though active site density from the MicroED structure of these crystals remained weak in the absence of the highest-affinity ligand (*SI Appendix*, Fig. S17 and Table S8). SSX structures of papain microcrystals soaked with each of the individual components of the mixture, E-64-A65, E315, E371, and E405, support these ED-MS results, where active site density in these structures confirmed E405 as a clear binder to papain (*SI Appendix*, Fig. S18 and Table S15).

### Determination of Papain Binders Directly from Crude Biosynthetic Reaction Mixtures by ED-MS.

**Fig. 5.**
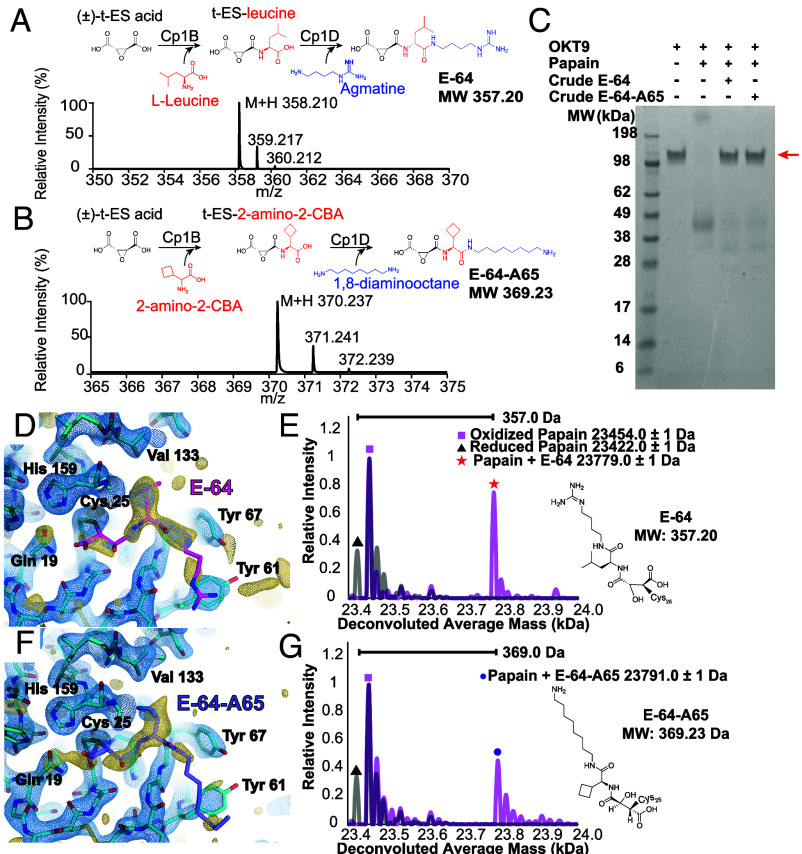
Strategy and demonstration of structural characterization of protein microcrystals soaked with crude biosynthetic products by ED-MS. Schematic for biosynthetic production of E-64 (*A*) and E-64-A65 inhibitors (*B*) with corresponding LCMS traces. Sodium dodecyl sulfate polyacrylamide gel electrophoresis (SDS-PAGE) demonstrating activity of papain in cleaving the 150 kDa antibody OKT9; when only papain and OKT9 are present, the 150 kDa band disappears indicating successful cleavage. When papain is pretreated with either crude preparation of biosynthetic products E-64 or E-64-A65, the 150 kDa band for OKT9 is retained indicating successful inhibition of the protease (*C*). MicroED structure of papain microcrystals soaked with this preparation of E-64 without additional purification, showing the active site at 2.3 Å resolution with density indicating the presence of the ligand (*D*), and nMS spectrum from this sample indicating the presence of the papain–E-64 complex (*E*). MicroED structure of papain microcrystals soaked with a similar crude E-64-A65 preparation without additional purification, showing the active site at 2.5 Å resolution (*F*), and nMS spectrum from this sample indicating the presence of the papain–E-64 complex (*G*). Blue mesh indicates the 2F_o_ − F_c_ map at 1.5σ level following modeling of the ligand, and gold mesh indicates the F_o_ − F_c_ map at 2,5σ level that was present prior to modeling a ligand. In *G* and *E*, grid-derived nMS spectra (magenta) are superimposed on the spectrum acquired from the apo-protein (gray).

Isolation of previously uncharacterized natural products is often nontrivial, and achieving comparable yields to commercially available compounds can be limiting. In such cases, we anticipate utility of ED-MS for discovery of binders from heterogeneous research samples, natural product extracts, and reactions performed as part of drug discovery efforts, despite the potentially low concentration of binders in those samples. To test this use-case, we soaked papain microcrystals directly with biosynthetic reaction mixtures, where E-64 and E-64-A65 were each the intended major product and were active against papain (Fig. 5 *A*–*C*). Still containing reaction precursors, side products, and enzyme catalysts, the crude solutions contained at most 1 mM of the intended E-64 product, and 2 mM of the E-64-A65 product (*SI Appendix*, Fig. S19). To compensate for this limited concentration, microcrystals were allowed to soak for a longer period of 4 h prior to freezing on cryoEM grids. MicroED data collection on these microcrystals yielded a 2.3 Å resolution structure of the crude E-64-soaked crystals (Fig. 5*D* and *SI Appendix*, Table S16), and a 2.5 Å resolution structure of the crude E-64-A65-soaked crystals (Fig. 5*F* and *SI Appendix*, Table S16). In each structure, prominent density in the active site indicated the presence of a bound inhibitor. nMS of the on-grid material once again revealed that the mass of the ligand-bound complex matched the papain–E-64 complex, for the crude E-64 soaking trial (Fig. 5*E*), and the papain–E-64-A65 complex, for the crude E-64-A65 trial (Fig. 5*G*). In neither experiment was a species corresponding to papain bound with a precursor or side product of the biosynthetic reaction detected.

### Application of ED-MS to β-Lactamase–Inhibitor Complexes.

**Fig. 6.**
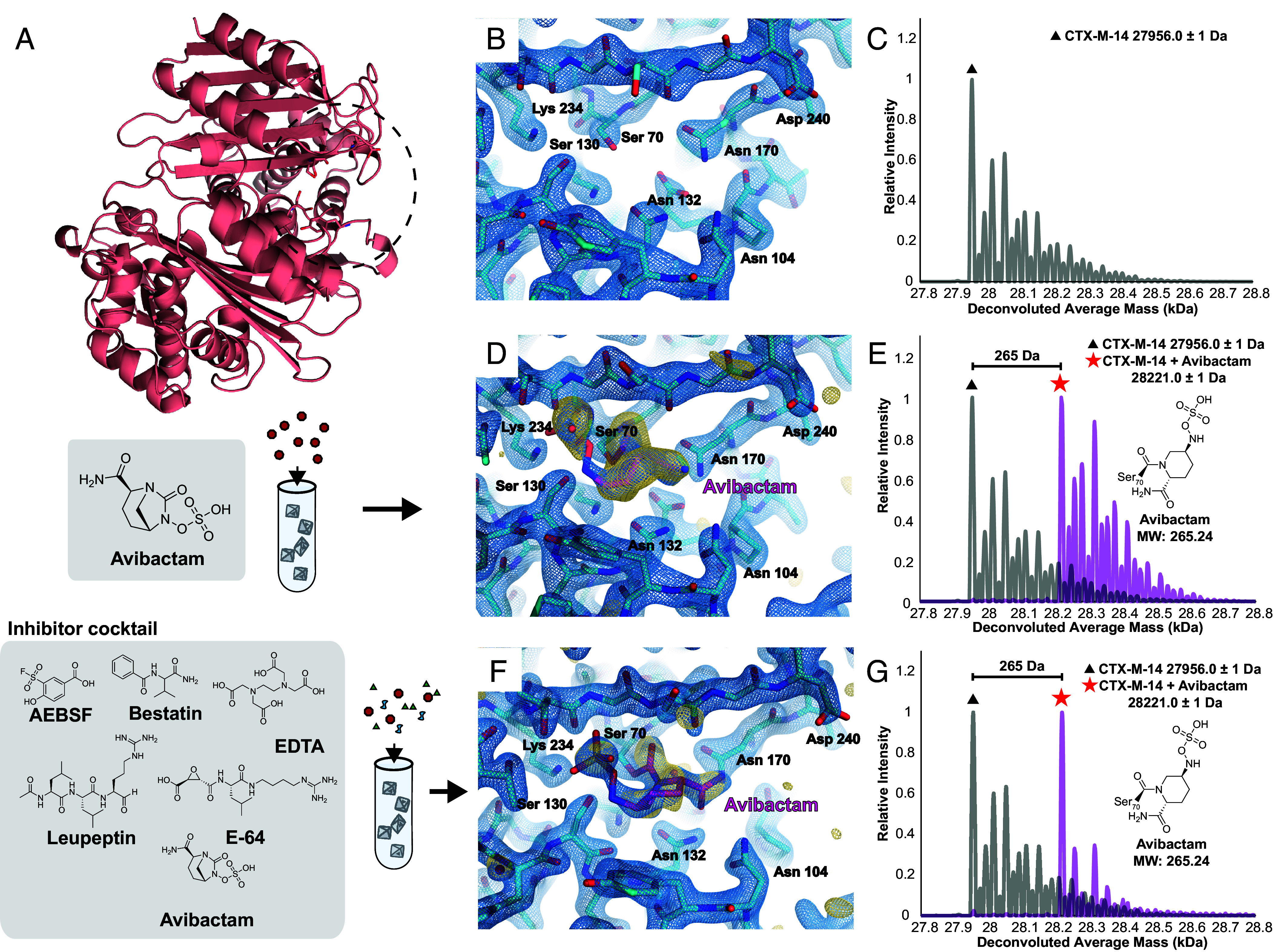
ED-MS resolves CTX-M-14 β-lactamase bound to the inhibitor avibactam. Atomic model of CTX-M-14 β-lactamase, determined by MicroED to 2.5 Å resolution with secondary structure and key active-site residue side chains rendered, and a dashed circle highlighting the antibiotic binding site (*A*). Enhanced view of the active site from the same model, with key residues labeled and the 2F_o_ − F_c_ map at 1.5σ level superimposed in blue (*B*). nMS spectrum acquired from the on-grid material that yielded this structure, with the highest peak indicating the anticipated mass of CTX-M-14 (*C*). View of the antibiotic binding site in a MicroED structure of CTX-M-14 cocrystallized with the covalent inhibitor avibactam, determined to 2.0 Å resolution (*D*), and corresponding nMS spectrum (magenta) superimposed on the spectrum acquired from the apo-form (gray) indicating shift corresponding to the mass of avibactam for the bound state (*E*). MicroED structure of CTX-M-14 microcrystals soaked with a mixture of avibactam and a protease inhibitor cocktail at 2.0 Å resolution, showing density for bound avibactam (*F*), and corresponding nMS spectrum indicating that avibactam is the primary binder in the cocktail (*G*). For protein–ligand structure graphics, blue mesh indicates the 2F_o_ − F_c_ map at 1.5σ level following modeling of the ligand (magenta coordinates), and gold mesh indicates the F_o_ − F_c_ map at the 3σ level that was present prior to modeling and refining the ligand.

We last aimed to assess the utility of ED-MS for resolving ligand binding in a protein of interest for drug development, the CTX-M-14 β-lactamase, referred to simply as CTX-M-14. We anticipated ED-MS might serve as a helpful tool to determine the structure of β-lactamase bound to the non-beta-lactam inhibitor, avibactam. Crystals of CTX-M-14 were grown, crushed into a slurry of nanocrystalline fragments, frozen on TEM grids and interrogated by MicroED (*SI Appendix*, Fig. S20). Using the same data collection routines established for papain, MicroED tilt series were acquired which yielded a 2.5 Å resolution structure of the apo-form of CTX-M-14 (Fig. 6 *A* and *B* and *SI Appendix*, Table S17). This structure was comparable to single-crystal XRD structures determined on macroscopic crystals grown from the same batches (*SI Appendix*, Fig. S21 and Table S18), and offered a clear view of the antibiotic binding pocket surrounding catalytic residue serine 70 (Fig. 6*B*). The nMS spectrum acquired from this grid, while somewhat afflicted by salt adducts due to the high potassium phosphate concentration in the crystals’ mother liquor, still showed its most prominent peak at 29567 Da, the anticipated mass of CTX-M-14, which we used as a reference for subsequent ligand binding studies by nMS (Fig. 6*C*). CTX-M-14 crystals soaked with avibactam yielded a 2.3 Å resolution MicroED structure of the complex, with clear density accounting for the inhibitor at high occupancy in each of the two binding sites within the asymmetric unit of the crystal (Fig. 6*D* and *SI Appendix*, Table S17). nMS from this on-grid material indicated a 265 Da mass shift of the most intense peak and the corresponding salt adduct series relative to the apo-form spectrum (Fig. 6*E*). No mass corresponding to the unliganded form of CTX-M-14 was detected by nMS in the inhibitor-soaked sample, indicating effectively complete saturation of its binding sites. We then extended that procedure to CTX-M-14 crystals soaked with a mixture of avibactam solution and protease inhibitor cocktail (Fig. 6 *F* and *G*). The results of this experiment, both from MicroED and nMS, were almost entirely consistent with those from the measurements taken on crystals soaked with pure avibactam. This indicated that avibactam binding to CTX-M-14 could be resolved by ED-MS just as readily from cocktail-soaking experiments as from crystals soaked with pure compound, and suggested that ED-MS paired with cocktail-soaked crystals might be of use for β-lactamase inhibitor discovery.

## Discussion

X-ray crystallography excels when large crystals of high quality can be grown, or when large volumes of concentrated crystal slurries are available. However, for microcrystalline samples, or where crystal quantity or yield may be limited, electron diffraction promises to be a valuable addition to this toolkit. MicroED has proven robust at delivering molecular-resolution structures of protein micro- and nanocrystals and has been applied to the determination of protein–small molecule complexes (41). Nonetheless, the application of MicroED to more systematic screening of bound drug-like ligands remains limited. Also understudied are the constraints of MicroED in this context, and the strategies for overcoming them to deliver accurate structural information about protein–ligand complexes.

We find that MicroED can be leveraged to determine molecular resolution structures of well-ordered enzyme microcrystals cocrystallized and soaked with a panel of potential ligands. Structures of protein–ligand complexes that afforded the most distinct density for bound ligands were determined from tilt series acquired on single microcrystals. Each crystal was exposed to less than 4.5 to 6.0 electrons per square Angstrom total fluence using 200 keV electrons, in order to mitigate beam-induced damage (36). Damage plays a role in data quality, since structures determined from data within this dose budget already showed evidence of radiation damage, including broken disulfide bonds, and required use of fast, sensitive direct electron counting detectors (36). Under these conditions, merging of partial datasets benefits completeness, but in turn reduces the clarity of ligand signal in the active site due to crystal nonisomorphism and variable occupancy between crystals. Although rare, the optimal condition is achieved when sufficiently complete data are collected from a single crystal, or when uniform occupancy is achieved across crystals. This is consistent with the relatively high ligand occupancies and well-clarified ligand density observed across the structures of CTX-M-14 β-lactamase in this study despite the persistent need for merging data, as nMS suggested these crystals were uniformly saturated with avibactam. Other efforts have likewise shown that when MicroED-associated radiation damage is mitigated, strongly bound ligands can be clearly resolved (42).

While MicroED can confirm a ligand’s presence in a target binding site, the observed density, as is often true for E-64, may be partial, lacking clarity at its more flexible, solvent-exposed terminus. For E-64 analogs and derivatives, this challenged unambiguous de novo identification. This limitation is not an issue when protein crystals are soaked with a solution of only one compound of interest, but becomes critical when they are soaked with mixtures; the latter are important for high-throughput screens, or when investigating less pure research samples. We find that complementing MicroED with nMS of grid-adsorbed crystals, following MicroED data collection, provides an efficient solution to this problem and enables unambiguous verification of which bound species are present in a sample of mixture-soaked microcrystals. In addition to reliably validating the identity of ligands present in structures from controlled soaking and cocrystallization experiments on papain and E-64 analogs, ED-MS was able to inform on papain complexed with mixtures of potential binders that MicroED alone may not have resolved. ED-MS also captured the binding of a clinically relevant inhibitor to M14 β-lactamase crystals soaked from an impure mixture of potential ligands, highlighting its applicability to targets of immediate therapeutic interest. Applying nMS to crystals used for MicroED might additionally reveal unexpected changes to the protein during crystallization, evidenced by the −18 Da mass shift between CTX-M-14 measured in solution deconvolution (*SI Appendix*, Fig. S22*G*) versus from on-grid microcrystals (*SI Appendix*, Fig. S22*H*). In cases where an unknown mass shift is present in the nMS deconvolution, the peak of interest can be isolated and fragmented to reveal the mass shift of the ligand and the apo state (*SI Appendix*, Fig. S23). Application of creative data collection strategies for limiting the impact of radiation damage on MicroED data might additionally improve the likelihood of de novo ligand identification (42) in cases where nMS is not available as a complementary method.

Despite the promise of ED-MS as a high-throughput solution for fragment-based drug discovery, it faces unsolved challenges. The labor-intensive nature of conventional MicroED can be a bottleneck. Automated routines for cryogenic MicroED data collection are becoming more effective (43), and comparable to those routinely applied to single particle CryoEM acquisition (44, 45). As these methods advance, we anticipate that the efficiency of data collection for ED-MS efforts will starkly improve and allow for more broad screening of potential ligands to a target than was achievable here. Additionally, depending on the identity of the target protein crystals, particular optimization of sample preparation conditions might often be necessary, as many common crystallization conditions contain molecules such as polyethylene glycol, which can be ion-suppressing for nMS. This is also true of common cryoprotectants such as glycerol. These agents shield protein signal during mass spectrometry measurement (46, 47) and are nontrivial to remove from crystals prior to study by nMS. This may also pose a challenge when studying ligands that are poorly soluble in water, as dimethyl sulfoxide (DMSO) may also pose these obstacles for nMS, and is commonly used to dissolve such compounds. We found in this study that volatile alcohols such as methanol are quite compatible with nMS sample preparation, and will solubilize some of these compounds, and may be used as alternative solvents. Optimization efforts aimed at circumventing the ion-suppression effect might benefit from ligand printing on grids at scale, facilitated by arrayED technologies (40), and from advances to sample preparation approaches that facilitate nMS data collection.

By allowing for the determination of ligand-bound protein structures directly from biosynthetic reaction mixtures, ED-MS shows promise for natural product-focused ligand discovery efforts, where compounds are often of limited concentration and purity. As demonstrated by our results, both covalent and noncovalent binders may be identified and structurally characterized by the method. This workflow can also be readily applied to automated ligand fragment screening, mimicking experimental schemes increasingly used in synchrotron and XFEL experiments but foregoing the requirements of rare beamtime and sufficiently large crystals. In such a case, MicroED would offer a first indication of binders present in a given mixture and would provide an essential picture of any binding sites detected on the target protein, while nMS verifies precisely which fragments or components of the mixture successfully bound. Finally, the application of nMS to TEM grid-adsorbed samples promises to be readily translatable to other cryoEM modalities, indicating potential for similarly correlative measurements to deliver unique insights in single-particle imaging and tomography experiments.

## Materials and Methods

### Protein Crystallization.

#### Papain.

Twice-crystallized papain extract from papaya latex in buffered aqueous suspension at approximately 25 mg/mL protein concentration was purchased from Sigma and used for crystallization experiments without further purification. Crystals formed by vapor diffusion in sitting drop trays, where the commercial papain suspension was mixed with methanol at a 1:2 volume ratio of papain to methanol. A volume of 15 μL was typically targeted in each well, incubated with a reservoir solution containing 59% methanol and 889 mM NaCl. Large single crystals suitable for single-crystal XRD would typically grow within 48 h under these conditions. Such crystals were resuspended in 5 to 10 μL of reservoir solution, crushed by repeated pipetting, and used to seed fresh crystals by transferring small amounts of crystal fragments with horsehair and streaking across a freshly prepared sitting drop prior to incubation. For cocrystallization experiments, each potential ligand was dissolved in 66% methanol and included in the crystallization well, such that it was present at 1.6 times molar excess of papain in the well.

#### Hen egg-white lysozyme (HEWL).

HEWL lyophilized powder was purchased from Sigma and resuspended at 100 mg/mL in 80 mM Sodium Acetate pH 4.8. Crystals were formed by vapor diffusion in sitting well drop trays. The HEWL solution was mixed 1:1 with 80 mM sodium acetate pH 4.8 for a final concentration of 30 μL in each well. The sitting drop was incubated with a reservoir containing 800 mM NaCl and 80 mM sodium acetate pH 4.8. Large crystals grew within 48 h. For cocrystallization with N,N′,N-triacetylchitotriose, 10 mM N,N′,N″-triacetylchitotriose was included in the crystallization buffer.

#### CTX-M-14 β-lactamase.

*Escherichia coli* BL21 cells were transformed with the plasmid pET-blaCTX-M and cultured in 2×YT medium supplemented with 2 M sorbitol, 50 mM betaine, and 50 μg/mL each of ampicillin and kanamycin. Cultures were incubated at 37 °C until an optical density of 0.6 (OD_600_) was reached, at which time protein expression was induced with the addition of 0.25 mM isopropyl β-D-1-thiogalactopyranoside (IPTG). Cultures were incubated overnight at 25 °C with vigorous shaking. Cells were harvested by centrifugation at 4,000× g for 15 min at 4 °C. The cell pellet was resuspended in lysis buffer containing 10 mM MES (pH 6.0) and 2 mM EDTA at 0.4 mS/cm conductivity. Cells were lysed by ultrasonication, and the lysate was clarified by centrifugation at 45,000 rpm for 1 h at 4 °C. Supernatant was diluted with lysis buffer to a conductivity of 1.4 mS before purification. Protein purification was carried out using ion exchange chromatography on a HiTrap CM Sepharose column equilibrated with 50 mM 2-(N-morpholino)ethanesulfonic acid (MES) buffer (pH 6.0) containing 2 mM EDTA, with a 0 to 0.15 M NaCl elution gradient. Eluted fractions were pooled, concentrated, and further purified using a HiPrep 16/60 Sephacryl S-300 HR size exclusion column in storage buffer (10 mM Tris pH 7.0, 50 mM NaCl, 2 mM EDTA). The protein was concentrated to 25.7 mg/mL and crystallized by vapor diffusion, either in hanging or sitting drop, against a reservoir solution of 1.4 M potassium phosphate pH 7.9. Large crystals suitable for single crystal XRD were grown by mixing the protein solution with the reservoir solution at a 1:1 volume ratio, while showers of smaller crystals suitable for MicroED could more rapidly be grown by adding preexisting CTX-M-14 β-lactamase microcrystal seeds to the drop.

### MicroED Sample Preparation.

#### Papain.

Crystals were resuspended in their sitting drops in a solution of 59% (v/v) methanol and 296 mM NaCl, and crushed by repeated pipetting to produce a 5 μL slurry of microcrystalline fragments. For structure determination of apo-form papain and ligand-soaked papain crystals, 0.5 μL of 500 mM DTT was added to the slurry. For each ligand soaking experiment, 4 μL of ligand solution (5.1 mM pure E-64, or between 1 to 2 mM crude E-64 or E65-A65) was added to the crystal slurry a set amount of time prior to plunge freezing. For pure E-64, samples were frozen 30 s, 4 min, and 10 min after the ligand was added. For microcrystals soaked with protease inhibitor cocktail, samples were frozen both 10 min and 4 h after adding the cocktail (purchased from Sigma as a powder and dissolved to its solubility limit). For microcrystals soaked with crude E-64 and crude E-64-A65, samples were frozen 4 h after ligand was added to the slurry. For papain–ligand cocrystals, no additional treatment was delivered after crushing crystals into slurries prior to freezing.

All cryoEM samples were prepared on R2/1 Quantifoil holey carbon grids with a copper mesh. Grids were glow discharged using a PELCO easiGlow system on each side for 30 s each, with a plasma current of 15 mA, then mounted in an FEI Vitrobot with a humidity-saturated chamber held at 12 °C. 1 μL of 66% methanol was loaded on the back side of each grid, and 1.5 μL crystal slurry was loaded on the front. Excess solvent was blotted away from the grid by manually contacting only the back side with a piece of filter paper gripped in a long pair of tweezers for 10 s. An additional 1 μL of 66% (v/v) methanol was then quickly added to the same side of the grid crystals had been applied to wash away excess salt from the mother liquor and immediately blotted away, once again by blotting only the back side of the grid. The grid was then plunge frozen in liquid ethane and stored under liquid nitrogen until transferred to the TEM.

#### HEWL.

HEWL crystals were resuspended in their reservoir solution and crushed by repeated pipetting. For ligand soaking experiments, crystal slurries prepared in this manner were mixed at a 1:1 volume ratio with 5 mM N,N′,N″-triacetylchitotriose, or with a 1:1 mixture of 10 mM N,N′,N″-triacetylchitotriose and saturated protease inhibitor cocktail from Sigma. 1 μL of reservoir solution was loaded on the back side of each grid, and 1.5 μL crystal slurry was loaded on the front. Grids were then backblotted as detailed above for papain and plunge-frozen.

#### CTX-M-14 β-lactamase.

CTX-M-14 β-lactamase crystals were resuspended in 0.5 M potassium phosphate pH 7.9 and crushed by repeated pipetting. For ligand soaking experiments, crystal slurries prepared in this manner were mixed at a 1:1 volume ratio with 5 mM avibactam, or with a 1:1 mixture of 10 mM avibactam and saturated protease inhibitor cocktail from Sigma. 1 μL of 0.5 M potassium phosphate pH 7.9 was loaded on the back side of each grid, and 1.5 μL crystal slurry was loaded on the front. Grids were then backblotted and plunge-frozen.

### MicroED Data Acquisition.

MicroED data were collected at a ThermoFisher Scientific Talos F200C TEM operating at 200 keV, equipped with both a Direct Electron Apollo detector with a native frame rate of 60 Hz (37), and a Ceta-D scintillator-based camera. A side-entry cryotransfer holder was used to preserve each grid used for data collection at 100 K. A typical data collection session spanned 2 to 4 h, where for each sample, a grid atlas was first acquired at 155× magnification on the DE Apollo using SerialEM (~30 min) (*SI Appendix*, Fig. S6 *D*, *G*, and *J*). Promising thin crystals with sharp edges were identified from these maps, navigated to, and a static diffraction pattern was acquired (*SI Appendix*, Fig. S6 *E*, *H*, and *K*). Crystals that diffracted well, producing many reflections to high scattering angles, were added to a list of potential targets (30 min to 1 h). At this stage, an assessment could be made on if the sample was likely to produce a series of useful diffraction datasets; if the distribution of crystals on the grid was too sparse, the vitreous ice layer surrounding the majority of crystals too thick, or the crystals themselves thicker than ~500 nm or damaged by the soaking treatment, the session was ended and a new sample prepared for the conditions of interest. Promising crystals were aligned to eucentricity at higher magnification using imaging-mode settings at 4,300× magnification, spot size 11, with the C2 lens condensed to ~44.8%. This configuration gave an electron beam flux density of approximately 0.01 e^−^/Å^2^s on the sample, which was suitable for minimizing dose delivered to each crystal prior to diffraction data collection. Diffraction was measured by centering each crystal of interest within a 100 μm diameter selected area aperture projecting an area approximately 3 μm in diameter onto the specimen, and illuminating the crystal in diffraction mode with a parallel beam, configured at spot size 10 with a C2 lens current of ~45.5% and a virtual camera length of 960 mm. This configuration produced an electron beam flux density of approximately 0.03 e^−^/Å^2^s on the sample. A single 3 s diffraction exposure was acquired on each crystal using the Ceta-D detector to assess whether it diffracted well, while subsequent acquisition was performed on the DE Apollo. Continuous-rotation tilt series in diffraction mode were acquired on each crystal targeting as wide an angular wedge as was achievable without the crystal of interest becoming obstructed, typically no more than 120°. Depending on an initial assessment of diffraction quality of the crystal, one of two data collection schemes was performed. For papain crystals that diffracted well, to high resolution, the stage rotation speed was set to 0.6°/s, paired with 90-frame integration on the DE Apollo, to produce a movie of images corresponding to 1.5 s effective exposures and 0.9°/frame oscillations through reciprocal space. For more weakly diffracting papain crystals, the stage rotation speed was set to 0.3°/s paired with 180-frame integration on the DE Apollo, as allowing greater electron flux per final frame of the movie positively influenced the measurable diffraction signal. However, this slower configuration typically necessitated the merging of more partial datasets to achieve high data completeness, as crystals would suffer from radiation damage prior to completing a tilt series. In general, for papain, the faster acquisition strategy was more ideal for recording high-completeness datasets with more favorable data reduction statistics. For all data acquired on HEWL and CTX-M-14 β-lactamase samples, the 0.3°/s modality was used, as radiation damage did not appear to drastically compromise datasets acquired in this manner. Acquisition of each diffraction movie took approximately 3 to 6 min. All movies were collected using a binning factor of two during acquisition, such that frames were 4,096 × 4,096 pixels in size. Following MicroED data collection, grids were recovered into liquid nitrogen and stored until they could be analyzed by nMS.

### MicroED Data Processing and Structure Determination.

All MicroED movies from the DE Apollo camera were saved in MRC file format and converted to SMV image stacks using a script developed in-house and run using MATLAB version 2023b. The only modification made to pixel values in these movies during conversion was a universal addition of 1 to all pixels, such that no counts of zero were present in the converted images. Indexing and data reduction was performed using XDS (48), and scaling and merging were performed using *XSCALE* (49). For each dataset, phase retrieval was achieved by molecular replacement with a known model of apo-form papain (PDB ID: 9PAP) (34), apo-form lysozyme (PDB ID: 1DPX) (50), or apo-form M-14 β-lactamase (PDB 1YLT) (51) using *PHASER* (52). Structure refinement was performed in *PHENIX* (53) by the following strategy: Reference model restraints from the original search model from the PDB were applied, while three cycles of refinement of XYZ coordinates in real and reciprocal space, group B-factors, and occupancies were performed. The model and map were then examined in *COOT*, and the model was edited to better agree with the measured data as indicated by maps in the F_o_ − F_c_ map. Water molecules were modeled in where positive peaks in the F_o_ − F_c_ map exceeding three sigma levels were found, if a hydrogen-bonding partner between 2.5 and 3.3 Å distance from the site was present and if they were not identified as close contacts with protein or other solvent atoms. Following these modifications, refinement was repeated in *PHENIX*, and this procedure was iteratively performed until no more modifications to the model were warranted. In the latter cycles of refinement, individual B-factors, rather than group B-factors, were refined only if doing so lowered the free R-factor and narrowed the gap between R_work_ and R_free_. For papain–ligand complexes, as much refinement as was possible was performed prior to modeling any ligand in the active site. If evidence of a ligand was present in the F_o_ − F_c_ map in the form of strong peaks stemming from the active cysteine 25 residue and exceeding three sigma levels, the ligand was modeled prior to the final cycles of refinement. A 1.8 Å restraint, with an allowed SD of 0.02 Å, on the bond distance between the sulfur atom of Cys25 and the covalently bound carbon atom of the ligand was applied in *PHENIX*, and the occupancies of all ligand atoms were refined together as a group. The same ligand building strategy was performed for ligand-bound structures of lysozyme and CTX-M-14. No bond length restraint was required for refinement of lysozyme ligand bound structures, while a 1.38 Å restraint, with an allowed SD of 0.02 Å was enforced between the oxygen atom of the serine 70 sidechain and the acyl-forming carbon of avibactam.

### nMS of On-Grid Material.

On-grid material was harvested by removing the grid from liquid nitrogen, allowing it to thaw, and immediately pipetting 7 µL of 150 mM ammonium acetate onto the grid surface. Repeated pipetting was done to dislodge and dissolve material on the grid, and once the sample was fully dissolved it was subjected to nMS analysis. Mass spectra were measured using a Thermo Q Exactive Plus UHMR Orbitrap instrument (Thermo Fisher Scientific, Bremen, Germany). NanoESI glass capillaries were pulled and coated with gold in-house. Samples were loaded into the gold coated capillary tubes and a 1.0 to 1.4 kV voltage was applied to the nanoESI source. All mass spectra are collected at a resolution of 12,500 at *m/z* 400 and instrument tuning parameters are listed in *SI Appendix*, Table S19. Native mass spectra were deconvoluted using UniDec (version 7.0.2), a software tool for Bayesian deconvolution of mass spectra (54). Spectra were imported as Thermo raw data and processed with the following parameters for each protein crystal target. *Papain*: mass range of 5,000 to 50,000 Da, charge state range of 1 to 20, *m/z* range from 2,000 to 4,000, with masses sampled every 1 Da. *HEWL:* mass range of 1,000 to 50,000 Da, charge state range of 1 to 20, m/z range from 1,500 to 3,000, with masses sampled every 1 Da. *CTX-M-14:* mass range of 1,000 to 50,000 Da, charge state range of 1 to 20, m/z range from 2,000 to 4,000, with masses sampled every 1 Da.

### Verification of On-Grid Protein Integrity within Crystals by Gel Electrophoresis.

Microcrystalline protein samples were harvested from TEM grids as described above. Protein solutions were diluted to 0.25 mg/mL in 100 mM ammonium acetate and a total of 2 μg was loaded into each well. All samples were brought to a final concentration of 100 mM DTT and 1× NuPAGE™ LDS Sample Buffer (4×). 10 μL of SeeBlue™ Plus2 Prestained Protein Standard was loaded into the ladder. All samples were loaded onto Invitrogen™ NuPAGE™ Bis-Tris Mini Protein Gels, 4 to 12%, 1.0 to 1.5 mm 9-well gel and ran at 110 V for 50 min. Gels were stained with SimplyBlue™ SafeStain for visualization.

### nMS of Protein–Ligand Binding in Solution.

All protein solutions were buffer exchanged into 150 mM ammonium acetate and diluted to 1 to 50 μM before loaded onto ESI tips. Ligands were added to the binding assays at an equimolar ratio of ligand to protein prior to nMS measurement.

### Single Crystal XRD and NMS of Macroscopic Crystals.

Macroscopic crystals, both unliganded and in complex with ligands, were mounted on Mitegen loops and flash-frozen under a nitrogen stream at 100 K. Ligand soaking and cocrystallization conditions matched the relative stoichiometry between protein and ligand used for the MicroED soaking experiments, though a longer 20 h soaking time was applied to allow sufficient time for the compound to diffuse through the crystal of interest. Complete XRD datasets were collected using a Rigaku FRE+ rotating anode X-ray diffractometer equipped with a Cu Kα source (λ = 1.54 Å) and a Rigaku HTC detector. Data were acquired with 2-min integrated exposures over 0.5° oscillations per frame, with total collection times of approximately 26 h per crystal. The detector was positioned at a distance of 78 mm from the crystal, mapping a resolution of 1.4 Å at the edge of the detector field.

Equivalent crystals were prepared for nMS by sequentially washing them in three 10 µL aliquots of 500 mM ammonium acetate to remove excess crystallization solution. Following the washes, crystals were dissolved in 10 µL of a working buffer containing 150 mM ammonium acetate to promote complete dissolution. The resulting samples were centrifuged at 10,000× g for 5 min to ensure the removal of any residual crystal fragments. The supernatant was then transferred into glass capillaries for analysis.

### Fixed-Target Serial Synchrotron X-Ray Crystallography Data Collection and Processing.

All SSX experiments were performed at the European Synchrotron Radiation Facility (ESRF) at beamline ID29 (55). 5 µL of a concentrated slurry of papain microcrystals, either in apo-form or presoaked with a solution of E-64 or natural product analogs, was loaded between two faces of mylar foil and allowed to spread by capillary action. This film was loaded onto a fixed target chip and rastered while illuminated by an 11.56 keV X-ray beam delivering a series of 90 µs pulses at >1.2 × 10^15^ photons per second flux. Diffraction was acquired using the *MXCuBE-Web* software (56) on a Jungfrau 4 M detector, and frames containing diffraction were identified in real-time by the *LimA2* software (57). Reflections from these hits were indexed using the *MOSFLM* (58) and *xGandalf* routines (59) facilitated by *CrystFEL* (60), with the most likely known unit cell and space group symmetry for papain enforced. Data were scaled in *CrystFEL*, after which phasing and refinement were performed by the same protocol as described above for single-crystal data.

### LC-MS of E-64 and Natural Product Analogs.

E-64 and analogs were diluted to a final concentration of 1 mg/mL, and 1 μL injections were used for each run. Samples were separated by reversed-phase liquid chromatography through a C18 column (Poroshell 120 HPLC Column 2.7 μm 3.0 × 50 mm) with a gradient flow from 0% ACN 0.1% formic acid to 100% ACN 0.1% formic acid at a flow rate of 0.6 mL/min over a course of 15 min. The samples were analyzed with an Agilent 6545 quadrupole time of flight (Q-TOF) LC-MS in positive ion mode with a 1260 Infinity LC system.

### Intact Protein LC–MS.

Samples were crystallized, and papain was purified using the same method employed for ED-MS. For the preparation, samples were incubated at 57 °C for 1 h under either reducing conditions (2 mM TCEP) or nonreducing conditions (no TCEP). Subsequently, 4 mM iodoacetamide was added to alkylate the protein, and the reaction was carried out for 30 min in the dark. The reaction was quenched with 8 mM TCEP. The final reaction volume was adjusted to 30 µL, with the protein concentration standardized to 1 mg/mL as determined by NanoDrop absorbance at A280. For LC–MS analysis, 5 µL of the 1 mg/mL sample was injected onto a C18 column (ZORBAX Reversed-Phase 2.7 μm 2.1 × 150 mm). Separation was performed using a gradient elution from 0% ACN 0.1% formic acid to 100% ACN 0.1% formic acid at a flow rate of 0.8 mL/min over 15 min on an Agilent 6530 Q-TOF LC-MS system in positive ion mode coupled with a 1260 Infinity LC system.

### Papain–Inhibitor Activity Inhibition Assay.

To test the inhibition efficiency of natural product E-64 analogs against papain, a series of cleavage inhibition assays were performed against the 150 kDa antibody OKT9, which is cleaved by papain. Solutions of OKT9, twice-crystallized papain extract, and E-64 analogs were diluted to working concentrations of 1 mg/mL, 1.25 mg/mL, and 2 mM respectively in digestion buffer (20 mM sodium phosphate pH 7.0, 10 mM EDTA). Reactions were carried out at a 1:10 papain:OTK9 mass ratio in 25 μL volumes testing cleavage ability in either the absence of E-64 analogs, or presence of E-64 analogs at 0.4 mM concentration. Reactions were carried out at 37 °C for 5 to 6 h before quenching by snap freezing the mixture in liquid nitrogen. Cleavage activity was assessed by SDS-PAGE, comparing inhibited and uninhibited lanes to OKT9 untreated with papain, where disappearance of a band at the 150 kDa position indicating OKT9 was taken as evidence of papain activity.

### Synthesis of Crude E-64 and E-64 Analogs.

Biosynthetic ligands were generated in one-pot reactions by adding precursor compounds to a solution containing the bioactive enzymes Cp1B and Cp1D, which catalyze the two-step reaction resulting in E-64 and its analogs. For the production of E-64, 100 μL reactions containing 50 mM sodium phosphate buffer 8.0, 25 μM Cp1B, 25 μM Cp1D, 5 mM (±)-*trans*-epoxy-succinic acid, 2.5 mM L-leucine, 5 mM agmatine, 10 mM ATP, and 10 mM MgCl_2_ were incubated at 30 °C for 16 h. Reactions producing E-64-A65 were prepared identically to those for E-64, but substituting the L-leucine group with 2.5 mM 2-amino-2-cyclobutylacetic acid and agmatine with 5 mM 1,8-diaminooctane. Following reaction completion, crude mixtures were spun through a 30 kDa Amicon concentrator tube to filter out the Cp1B and Cp1D enzymes. The flow-through from these spins containing the product inhibitors were used in subsequent experiments.

### ArrayED Ligand Printing and On-Grid Soaking.

Using a Scienion S3 microarrayer, droplets of a ~26 mM solution of E-64 (250 to 350 pL volume per drop) were automatically printed onto Quantifoil R2/2 Formvar/Carbon 200 Mesh Cu TEM grids in a 12 × 12 pattern, with one droplet per grid square (*SI Appendix*, Fig. S15 *A* and *B*). After drying, the printing process was repeated on the same grid to increase the amount of E-64 present. The grids were allowed to dry and stored under ambient conditions at room temperature until used for mixing with papain crystals and freezing. 2 μL of 66% methanol was added to the arrayed face of the grid and mixed for approximately 1 min by repeated pipetting. Grids were mounted in an FEI Vitrobot with a humidity-saturated chamber held at 12 °C. 1 μL of 66% methanol was loaded on the back side of each grid, and 1.5 μL crystal slurry pretreated with DTT was loaded on the front. The microcrystal slurry was allowed to mix with E-64 by incubating on the grid within the Vitrobot chamber for 5 min. After this waiting period, the grid was manually back blotted as described above, and plunge frozen into liquid ethane (*SI Appendix*, Fig. S15*A*).

## Supplementary Material

Appendix 01 (PDF)

## Data Availability

Structures are available via PDB entries as follows. MicroED structures of papain and papain–ligand complexes: 9NAG (61), 9NAE (62), 9NAR (63), 9NAO (64), 9N9D (65), 9NBQ (66), 9NC1 (67), 9NAX (68), 9NAY (69), and 9NBP (70). MicroED structures of lysozyme and lysozyme–ligand complexes: 9ORZ (71), 9OS1 (72), 9OS8 (73), and 9OS0 (74). MicroED structures of CTX-M-14 β-lactamase and β-lactamase–ligand complexes: 9ORG (75), 9ORS (76), 9ORL (77), and 9ORH (78). Single crystal X-ray diffraction structures of papain–ligand complexes: 9NB2 (79) and 9NAT (80). SSX structures of papain and papain–ligand complexes: 9NCA (81), 9NBF (82), 9NBJ (83), 9NBK (84), 9NBN (85), 9NB4 (86), and 9NB7 (87). Single-crystal XRD structures of lysozyme and lysozyme–ligand complexes: 9ORW (88), 9ORV (89), 9ORY (90), and 9ORX (91). Single-crystal XRD structures of CTX-M-14 β-lactamase and β-lactamase–ligand complexes: 9OQE (92), 9OR3 (93), 9OR7 (94), and 9ORB (95). MicroED structure of E-64D is available via CCDC deposition number 2423833 (96). Diffraction data are available via Zenodo entries: 15527919 (lysozyme MicroED data) (97), 14876608 (papain MicroED data) (98), 15529745 (CTX-M-14 β-lactamase MicroED data) (99), 15530247 (lysozyme and CTX-M-14 β-lactamase single-crystal XRD data) (100), and 14876819 (papain single-crystal XRD data) (101). SSX data are available on the ESRF data portal under DOI: 10.15151/ESRF-DC-2206265994 (102). Files associated with additional structures referenced in this report but not deposited in the PDB are available via Zenodo entries 14885217 (103) and 15530411 (104). Native mass spectrometry data are available via Zenodo entry 15522990 (105).

## References

[1] L. Damian, “Isothermal titration calorimetry for studying protein-ligand interactions” in Protein-Ligand Interactions, M. A. Williams, T. Daviter, Eds. (Methods in Molecular Biology, Humana Press, 2013), pp. 103–118. 10.1007/978-1-62703-398-5_4 23729250

[2] N. O’Connell, “Protein ligand interactions using surface plasmon resonance” in Targeted Protein Degradation, A. M. Cacace, C. M. Hickey, M. Békés, Eds. (Methods in Molecular Biology, Springer, US, 2021), pp. 3–20. 10.1007/978-1-0716-1665-9_1 34432236

[3] K. Ishii, M. Noda, S. Uchiyama, Mass spectrometric analysis of protein–ligand interactions. Biophysics **13**, 87–95 (2016). 10.2142/biophysico.13.0_87 PMC5042164 27924262

[4] J. Nonomiya, K. S. Li, B. M. Babin, M. M. Mulvihill, Covalent library screening by targeted mass spectrometry for rapid binding site identification. Anal. Chem. **95**, 3779–3788 (2023). 36706310 10.1021/acs.analchem.2c04967

[5] J. Fejzo, C. Lepre, X. Xie, Application of NMR screening in drug discovery. Curr. Top. Med. Chem. **3**, 81–97 (2003). 12570779 10.2174/1568026033392796

[6] M. J. Harner, A. O. Frank, S. W. Fesik, Fragment-based drug discovery using NMR spectroscopy. J. Biomol. NMR **56**, 65–75 (2013). 23686385 10.1007/s10858-013-9740-z PMC3699969

[7] J. J. Ziarek, F. C. Peterson, B. L. Lytle, B. F. Volkman, “Binding site identification and structure determination of protein-ligand complexes by NMR” in Methods in Enzymology (Elsevier, 2011), pp. 241–275. 10.1016/B978-0-12-381274-2.00010-8 PMC3635485 21371594

[8] Z. Chilingaryan, Z. Yin, A. J. Oakley, Fragment-based screening by protein crystallography: Successes and pitfalls. Int. J. Mol. Sci. **13**, 12857–12879 (2012). 23202926 10.3390/ijms131012857 PMC3497300

[9] J. M. Holton, K. A. Frankel, The minimum crystal size needed for a complete diffraction data set. Acta Crystallogr. D Biol. Crystallogr. **66**, 393–408 (2010). 20382993 10.1107/S0907444910007262 PMC2852304

[10] J.-P. Colletier , De novo phasing with X-ray laser reveals mosquito larvicide BinAB structure. Nature **539**, 43–47 (2016). 27680699 10.1038/nature19825 PMC5161637

[11] A. S. Brewster , Indexing amyloid peptide diffraction from serial femtosecond crystallography: New algorithms for sparse patterns. Acta Crystallogr. D Biol. Crystallogr. **71**, 357–366 (2015). 25664747 10.1107/S1399004714026145 PMC4321489

[12] P. Roedig , High-speed fixed-target serial virus crystallography. Nat. Methods **14**, 805–810 (2017). 28628129 10.1038/nmeth.4335 PMC5588887

[13] F. Stellato , Room-temperature macromolecular serial crystallography using synchrotron radiation. IUCrJ **1**, 204–212 (2014). 10.1107/S2052252514010070 PMC4107920 25075341

[14] D. Shi, B. L. Nannenga, M. G. Iadanza, T. Gonen, Three-dimensional electron crystallography of protein microcrystals. Elife **2**, e01345 (2013). 24252878 10.7554/eLife.01345 PMC3831942

[15] I. Nederlof, E. van Genderen, Y.-W. Li, J. P. Abrahams, A Medipix quantum area detector allows rotation electron diffraction data collection from submicrometre three-dimensional protein crystals. Acta Crystallogr. Sect. D Biol. Crystallogr. **69**, 1223–1230 (2013). 23793148 10.1107/S0907444913009700 PMC3689525

[16] K. Yonekura, K. Kato, M. Ogasawara, M. Tomita, C. Toyoshima, Electron crystallography of ultrathin 3D protein crystals: Atomic model with charges. Proc. Natl. Acad. Sci. U.S.A. **112**, 3368–3373 (2015). 25730881 10.1073/pnas.1500724112 PMC4372003

[17] M. T. B. Clabbers, S. Z. Fisher, M. Coinçon, X. Zou, H. Xu, Visualizing drug binding interactions using microcrystal electron diffraction. Commun. Biol. **3**, 417 (2020). 32737395 10.1038/s42003-020-01155-1 PMC7395157

[18] M. W. Martynowycz, T. Gonen, Ligand incorporation into protein microcrystals for MicroED by on-grid soaking. Structure **29**, 88–95.e2 (2021). 33007196 10.1016/j.str.2020.09.003 PMC7796918

[19] D. A. Erlanson, S. W. Fesik, R. E. Hubbard, W. Jahnke, H. Jhoti, Twenty years on: The impact of fragments on drug discovery. Nat. Rev. Drug Discov. **15**, 605–619 (2016). 27417849 10.1038/nrd.2016.109

[20] L. R. De Souza Neto , Fragment library screening by X-ray crystallography and binding site analysis on thioredoxin glutathione reductase of Schistosoma mansoni. Sci. Rep. **14**, 1582 (2024). 38238498 10.1038/s41598-024-52018-2 PMC10796382

[21] C. Verlinde , Fragment-based cocktail crystallography by the Medical Structural Genomics of Pathogenic Protozoa Consortium. Curr. Top. Med. Chem. **9**, 1678–1687 (2009). 19929835 10.2174/156802609790102383 PMC2897734

[22] D. Patel, J. D. Bauman, E. Arnold, Advantages of crystallographic fragment screening: Functional and mechanistic insights from a powerful platform for efficient drug discovery. Prog. Biophys. Mol. Biol. **116**, 92–100 (2014). 25117499 10.1016/j.pbiomolbio.2014.08.004 PMC4501029

[23] S. L. Cohen, B. T. Chait, Mass spectrometry as a tool for protein crystallography. Annu. Rev. Biophys. Biomol. Struct. **30**, 67–85 (2001). 11340052 10.1146/annurev.biophys.30.1.67

[24] S. Tamara, M. A. Den Boer, A. J. R. Heck, High-resolution native mass spectrometry. Chem. Rev. **122**, 7269–7326 (2022). 34415162 10.1021/acs.chemrev.1c00212 PMC9052423

[25] J. B. Fenn, M. Mann, C. K. Meng, S. F. Wong, C. M. Whitehouse, Electrospray ionization for mass spectrometry of large biomolecules. Science **246**, 64–71 (1989). 2675315 10.1126/science.2675315

[26] A. F. M. Gavriilidou, K. Sokratous, H.-Y. Yen, L. Colibus, High-throughput native mass spectrometry screening in drug discovery. Front. Mol. Biosci. **9**, 1–15 (2022). 10.3389/fmolb.2022.837901 PMC9047894 35495635

[27] K. I. Varughese , Crystal structure of a papain-E-64 complex. Biochemistry **28**, 1330–1332 (1989). 2713367 10.1021/bi00429a058

[28] M. J. Kim , Crystal structure of papain-E64-c complex. Binding diversity of E64-c to papain S2 and S3 subsites. Biochem. J. **287**, 797–803 (1992). 1445241 10.1042/bj2870797 PMC1133078

[29] M. Liu , Enzymatic combinatorial synthesis of E-64 and related cysteine protease inhibitors. Nat Chem Biol. (2025), 10.1038/s41589-025-01907-2. PMC12568646 40346252

[30] A. Matagne, A. Dubus, M. Galleni, J.-M. Frère, The β-lactamase cycle: A tale of selective pressure and bacterial ingenuity. Nat. Prod. Rep. **16**, 1–19 (1999). 10101880 10.1039/a705983c

[31] Y. Chen, B. Shoichet, R. Bonnet, Structure, function, and inhibition along the reaction coordinate of CTX-M β-lactamases. J. Am. Chem. Soc. **127**, 5423–5434 (2005). 15826180 10.1021/ja042850a PMC1360657

[32] L. M. C. Jacobs, P. Consol, Y. Chen, Drug discovery in the field of β-lactams: An academic perspective. Antibiotics **13**, 59 (2024). 38247618 10.3390/antibiotics13010059 PMC10812508

[33] D. E. Ehmann , Avibactam is a covalent, reversible, non–β-lactam β-lactamase inhibitor. Proc. Natl. Acad. Sci. U.S.A. **109**, 11663–11668 (2012). 22753474 10.1073/pnas.1205073109 PMC3406822

[34] I. G. Kamphuis, K. H. Kalk, M. B. A. Swarte, J. Drenth, Structure of papain refined at 1.65 Å resolution. J. Mol. Biol. **179**, 233–256 (1984). 6502713 10.1016/0022-2836(84)90467-4

[35] J. Hattne, M. T. B. Clabbers, M. W. Martynowycz, T. Gonen, Electron counting with direct electron detectors in MicroED. Structure **31**, 1504–1509.e1 (2023). 37992709 10.1016/j.str.2023.10.011 PMC10756876

[36] N. Vlahakis , Fast event-based electron counting for small-molecule structure determination by MicroED. Acta Crystallogr. C Struct. Chem. **81**, 116–130 (2025). 39982366 10.1107/S2053229624012300 PMC11881165

[37] R. Peng , Characterizing the resolution and throughput of the Apollo direct electron detector. J. Struct. Biol. X **7**, 1–10 (2023). 10.1016/j.yjsbx.2022.100080 PMC9791170 36578473

[38] E. Schröder, C. Phillips, E. Garman, K. Harlos, C. Crawford, X-ray crystallographic structure of a papain-leupeptin complex. FEBS Lett. **315**, 38–42 (1993). 8416808 10.1016/0014-5793(93)81128-m

[39] A. Rydholm, K. Anseth, C. Bowman, Effects of neighboring sulfides and pH on ester hydrolysis in thiol–acrylate photopolymers. Acta Biomater. **3**, 449–455 (2007). 17276150 10.1016/j.actbio.2006.12.001 PMC2041895

[40] D. A. Delgadillo , High-throughput identification of crystalline natural products from crude extracts enabled by microarray technology and microED. ACS Cent. Sci. **10**, 176–183 (2024). 38292598 10.1021/acscentsci.3c01365 PMC10823509

[41] J. Lin, M. J. Gallenito, J. Hattne, T. Gonen, Ligand screening and discovery using cocktail soaking and automated microcrystal electron diffraction. ChemMedChem **20**, e202500156 (2025), 10.1002/cmdc.202500156. 40139972 PMC12174747

[42] A. Shaikhqasem , Strategies for mitigating radiation damage and improving data completeness in 3D electron diffraction of protein crystals. bioRxiv [Preprint] (2025). 10.1101/2025.02.06.636927 (Accessed 23 February 2025). PMC12809435 41410349

[43] D. Eremin , Spatially-aware diffraction mapping enables fully autonomous MicroED. ChemRxiv [Preprint] (2025). 10.26434/chemrxiv-2025-4p4c3 (Accessed 19 May 2025). 41211926

[44] M. J. De La Cruz, “Automation of continuous-rotation data collection for MicroED” in cryoEM, T. Gonen, B. L. Nannenga, Eds. (Methods in Molecular Biology, Springer, US, 2021), pp. 321–327. 10.1007/978-1-0716-0966-8_16 33368012

[45] R. Bücker , Serial protein crystallography in an electron microscope. Nat. Commun. **11**, 996 (2020). 32081905 10.1038/s41467-020-14793-0 PMC7035385

[46] C. Zhao, P. B. O’Connor, Removal of polyethylene glycols from protein samples using titanium dioxide. Anal. Biochem. **365**, 283–285 (2007). 17462581 10.1016/j.ab.2007.03.024 PMC1949484

[47] M. A. Mendes, J. M. Chies, A. C. D. O. Dias, S. A. Filho, M. S. Palma, The shielding effect of glycerol against protein ionization in electrospray mass spectrometry. Rapid Commun. Mass Spectrom. **17**, 672–677 (2003). 12661019 10.1002/rcm.958

[48] W. Kabsch, XDS. Acta Crystallogr. D Biol. Crystallogr. **66**, 125–132 (2010). 20124692 10.1107/S0907444909047337 PMC2815665

[49] W. Kabsch, Integration, scaling, space-group assignment and post-refinement. Acta Crystallogr. D Biol. Crystallogr. **66**, 133–144 (2010). 20124693 10.1107/S0907444909047374 PMC2815666

[50] M. S. Weiss, G. J. Palm, R. Hilgenfeld, Crystallization, structure solution and refinement of hen egg-white lysozyme at pH 8.0 in the presence of MPD. Acta Crystallogr. D Biol. Crystallogr. **56**, 952–958 (2000). 10944331 10.1107/s0907444900006685

[51] Y. Chen, J. Delmas, J. Sirot, B. Shoichet, R. Bonnet, Atomic resolution structures of CTX-M β-lactamases: Extended spectrum activities from increased mobility and decreased stability. J. Mol. Biol. **348**, 349–362 (2005). 15811373 10.1016/j.jmb.2005.02.010

[52] A. J. McCoy , *Phaser* crystallographic software. J. Appl. Crystallogr. **40**, 658–674 (2007). 19461840 10.1107/S0021889807021206 PMC2483472

[53] P. D. Adams , PHENIX: A comprehensive Python-based system for macromolecular structure solution. Acta Crystallogr D Biol Crystallogr **66**, 213–221 (2010). 20124702 10.1107/S0907444909052925 PMC2815670

[54] M. T. Marty , Bayesian deconvolution of mass and ion mobility spectra: From binary interactions to polydisperse ensembles. Anal. Chem. **87**, 4370–4376 (2015). 25799115 10.1021/acs.analchem.5b00140 PMC4594776

[55] J. Orlans , Advancing macromolecular structure determination with microsecond X-ray pulses at a 4th generation synchrotron. Commun. Chem. **8**, 6 (2025). 39775172 10.1038/s42004-024-01404-y PMC11707155

[56] M. Oscarsson , MXCuBE2: The dawn of MXCuBE collaboration. J. Synchrotron Rad. **26**, 393–405 (2019). 10.1107/S1600577519001267 PMC6412183 30855248

[57] S. Debionne , “LImA2: Edge distributed acquisition and processing framework for high performance 2D detectors” in *19th Biennial International Conference on Accelerator and Large Experimental Physics Control Systems* (JACoW Publishing, 2023), vol. 226-0358, pp. 1269–1274.

[58] T. G. G. Battye, L. Kontogiannis, O. Johnson, H. R. Powell, A. G. W. Leslie, iMOSFLM: A new graphical interface for diffraction-image processing with MOSFLM. Acta Crystallogr. D Biol. Crystallogr. **67**, 271–281 (2011). 21460445 10.1107/S0907444910048675 PMC3069742

[59] Y. Gevorkov , XGANDALF–Extended gradient descent algorithm for lattice finding. Acta Crystallogr. A Found. Adv. **75**, 694–704 (2019). 31475914 10.1107/S2053273319010593 PMC6718201

[60] T. A. White , Recent developments in *CrystFEL*. J. Appl. Crystallogr. **49**, 680–689 (2016). 27047311 10.1107/S1600576716004751 PMC4815879

[61] N. Vlahakis, J. A. Rodriguez, MicroED structure of the apo-form of papain. RCSB Protein Data Bank. 10.2210/pdb9NAG/pdb. Deposited 12 February 2025.

[62] N. Vlahakis, J. A. Rodriguez, MicroED structure of papain co-crystallized with E-64. RCSB Protein Data Bank. 10.2210/pdb9NAE/pdb. Deposited 11 February 2025.

[63] N. W. Vlahakis, J. A. Rodriguez, MicroED structure of papain microcrystals soaked with E-64 for 10 minutes. RCSB Protein Data Bank. 10.2210/pdb9NAR/pdb. Deposited 12 February 2025.

[64] N. W. Vlahakis, J. A. Rodriguez, MicroED structure of papain complexed with natural product E64-A65. RCSB Protein Data Bank. 10.2210/pdb9NAO/pdb. Deposited 12 February 2025.

[65] N. W. Vlahakis, J. A. Rodriguez, MicroED structure of papain co-crystallized with E-64C. RCSB Protein Data Bank. 10.2210/pdb9N9D/pdb. Deposited 10 February 2025.

[66] N. Vlahakis, J. A. Rodriguez, MicroED structure of papain co-crystallized with E-64D. RCSB Protein Data Bank. 10.2210/pdb9NBQ/pdb. Deposited 14 February 2025.

[67] N. Vlahakis, J. A. Rodriguez, MicroED structure of papain-E-64 complex from microcrystals soaked with protease inhibitor cocktail. RCSB Protein Data Bank. 10.2210/pdb9NC1/pdb. Deposited 14 February 2025.

[68] N. W. Vlahakis, J. A. Rodriguez, MicroED structure of the papain-E-64 complex from microcrystals soaked with crude biosynthetic reaction mixture. RCSB Protein Data Bank. 10.2210/pdb9NAX/pdb. Deposited 13 February 2025.

[69] N. W. Vlahakis, J. A. Rodriguez, MicroED structure of papain complexed with natural product E-64-A65 from microcrystals soaked in crude biosynthetic reaction mixture. RCSB Protein Data Bank. 10.2210/pdb9NAY/pdb. Deposited 13 February 2025.

[70] N. Vlahakis, J. A. Rodriguez, MicroED structure of the papain-E-64 complex from microcrystals mixed on-grid with microarrayed ligand. RCSB Protein Data Bank. 10.2210/pdb9NBP/pdb. Deposited 14 February 2025.

[71] N. W. Vlahakis, C. W. Flowers, J. A. Rodriguez, MicroED structure of apo-form lysozyme. RCSB Protein Data Bank. 10.2210/pdb9ORZ/pdb. Deposited 23 May 2025.

[72] N. W. Vlahakis, C. W. Flowers, J. A. Rodriguez, MicroED structure of lysozyme co-crystallized with N,N',N"-triacetylchitotriose. RCSB Protein Data Bank. 10.2210/pdb9OS1/pdb. Deposited 23 May 2025.

[73] N. W. Vlahakis, C. W. Flowers, J. A. Rodriguez, MicroED structure of lysozyme soaked with N,N',N"-triacetylchitotriose. RCSB Protein Data Bank. 10.2210/pdb9OS8/pdb. Deposited 23 May 2025.

[74] N. W. Vlahakis, C. W. Flowers, J. A. Rodriguez, MicroED structure of lysozyme complexed with N,N',N"-triacetylchitotriose from cocktail-soaked crystals. RCSB Protein Data Bank. 10.2210/pdb9OS0/pdb. Deposited 23 May 2025.

[75] N. Vlahakis, J. A. Rodriguez, L. M. C. Jacobs, Y. Chen, MicroED structure of apo-form CTX-M-14 beta-lactamase. RCSB Protein Data Bank. 10.2210/pdb9ORG/pdb. Deposited 22 May 2025.

[76] N. W. Vlahakis, J. A. Rodriguez, L. M. C. Jacobs, Y. Chen, MicroED structure of CTX-M-14 beta-lactamase co-crystallized with avibactam. RCSB Protein Data Bank. 10.2210/pdb9ORS/pdb. Deposited 22 May 2025.

[77] N. W. Vlahakis, J. A. Rodriguez, L. M. C. Jacobs, Y. Chen, MicroED structure of CTX-M-14 beta-lactamase soaked with avibactam. RCSB Protein Data Bank. 10.2210/pdb9ORL/pdb. Deposited 22 May 2025.

[78] N. Vlahakis, J. A. Rodriguez, L. M. C. Jacobs, Y. Chen, MicroED structure of the CTX-M-14 beta-lactamase-avibactam complex from inhibitor cocktail-soaked crystals. RCSB Protein Data Bank. 10.2210/pdb9ORH/pdb. Deposited 22 May 2025.

[79] N. W. Vlahakis, J. A. Rodriguez, X-ray diffraction structure of papain soaked with E-64. RCSB Protein Data Bank. 10.2210/pdb9NB2/pdb. Deposited 13 February 2025.

[80] N. W. Vlahakis, N. W. Flowers, J. A. Rodriguez, X-ray diffraction structure of papain co-crystallized with leupeptin. RCSB Protein Data Bank. 10.2210/pdb9NAT/pdb. Deposited 12 February 2025.

[81] N. Vlahakis, J. A. Rodriguez, MicroED structure of microcrystals soaked with a mixture of E-64, E-64C, and E-64D. RCSB Protein Data Bank. 10.2210/pdb9NCA/pdb. Deposited 15 February 2025.

[82] N. W. Vlahakis, J. A. Summers, S. Wakatsuki, J. A. Rodriguez, Serial synchrotron X-ray diffraction structure of papain microcrystals soaked with E-64. RCSB Protein Data Bank. 10.2210/pdb9NBF/pdb. Deposited 13 February 2025.

[83] N. W. Vlahakis, J. A. Summers, S. Wakatsuki, J. A. Rodriguez, Serial synchrotron X-ray diffraction structure of papain microcrystals soaked with E-64C. RCSB Protein Data Bank. 10.2210/pdb9NBJ/pdb. Deposited 13 February 2025.

[84] N. W. Vlahakis, J. A. Summers, S. Wakatsuki, J. A. Rodriguez, Serial synchrotron X-ray diffraction structure of papain microcrystals soaked with E-64D. RCSB Protein Data Bank. 10.2210/pdb9NBK/pdb. Deposited 13 February 2025.

[85] N. W. Vlahakis, J. A. Summers, S. Wakatsuki, J. A. Rodriguez, Serial synchrotron X-ray diffraction structure of papain microcrystals soaked with a mixture of E-64, E-64C, and E-64D. RCSB Protein Data Bank. 10.2210/pdb9NBN/pdb. Deposited 14 February 2025.

[86] N. W. Vlahakis, J. A. Summers, S. Wakatsuki, J. A. Rodriguez, Serial synchrotron X-ray diffraction structure of papain microcrystals soaked with natural product E-64-A65. RCSB Protein Data Bank. 10.2210/pdb9NB4/pdb. Deposited 13 February 2025.

[87] N. W. Vlahakis, J. A. Summers, S. Wakatsuki, J. A. Rodriguez, Serial synchrotron X-ray diffraction structure of papain microcrystals soaked with natural product E405. RCSB Protein Data Bank. 10.2210/pdb9NB7/pdb. Deposited 13 February 2025.

[88] C. W. Flowers, N W. Vlahakis, J. A. Rodriguez, X-ray diffraction structure of apo-form lysozyme. RCSB Protein Data Bank. 10.2210/pdb9ORW/pdb. Deposited 23 May 2025.

[89] C. W. Flowers, N. W. Vlahakis, J. A. Rodriguez, X-ray diffraction structure of lysozyme co-crystallized with N,N',N"-triacetylchitotriose. RCSB Protein Data Bank. 10.2210/pdb9ORV/pdb. Deposited 22 May 2025.

[90] C. W. Flowers, N. W. Vlahakis, J. A. Rodriguez, X-ray diffraction structure of lysozyme soaked with N,N',N"-triacetylchitotriose. RCSB Protein Data Bank. 10.2210/pdb9ORY/pdb. Deposited 23 May 2025.

[91] C. W. Flowers, N. W. Vlahakis, J. A. Rodriguez, X-ray diffraction structure of lysozyme complexed with N,N',N"-triacetylchitotriose from a cocktail-soaked crystal. RCSB Protein Data Bank. 10.2210/pdb9ORX/pdb. Deposited 23 May 2025.

[92] N. W. Vlahakis, J. A. Rodriguez, L. M. C. Jacobs, Y. Chen, X-ray diffraction structure of apo-form CTX-M-14 beta-lactamase. RCSB Protein Data Bank. 10.2210/pdb9OQE/pdb. Deposited 20 May 2025.

[93] N. W. Vlahakis, J. A. Rodriguez, L. M. C. Jacobs, Y. Chen, X-ray diffraction structure of CTX-M-14 beta-lactamase co-crystallized with avibactam. RCSB Protein Data Bank. 10.2210/pdb9OR3/pdb. Deposited 21 May 2025.

[94] N. W. Vlahakis, J. A. Rodriguez, L. M. C. Jacobs, Y. Chen, X-ray diffraction structure of CTX-M-14 beta-lactamase soaked with avibactam. RCSB Protein Data Bank. 10.2210/pdb9OR7/pdb. Deposited 21 May 2025.

[95] N. W. Vlahakis, J. A. Rodriguez, L. M. C. Jacobs, Chen, Y. X-ray diffraction structure of the CTX-M-14 beta-lactamase-avibactam complex an inhibitor cocktail-soaked crystal. RCSB Protein Data Bank. 10.2210/pdb9ORB/pdb. Deposited 21 May 2025.

[96] N. Vlahakis, J. A. Rodriguez, CCDC 2423833: Experimental Crystal Structure Determination. CCDC. 10.5517/ccdc.csd.cc2mc65d. Deposited 14 February 2025.

[97] N. Vlahakis, C. Flowers, J. Rodriguez, ED-MS MicroED: Electron diffraction tilt series from microcrystals of lysozyme and lysozyme-ligand complexes [Data set]. Zenodo. 10.5281/zenodo.15527919. Deposited 27 May 2025.

[98] N. Vlahakis, ED-MS MicroED: Electron diffraction tilt series from microcrystals of papain and papain-ligand complexes [Data set]. Zenodo. 10.5281/zenodo.14876608. Deposited 11 February 2025.

[99] N. Vlahakis, J. Rodriguez, ED-MS MicroED: Electron diffraction tilt series from microcrystals of CTX-M-14 beta-lactamase and CTX-M-14 complexes with avibactam [Data set]. Zenodo. 10.5281/zenodo.15529745. Deposited 27 May 2025.

[100] N. Vlahakis, C. Flowers, J. Rodriguez, ED-MS MicroED: Single crystal X-ray diffraction datasets acquired from lysozyme, CTX-M-14 beta-lactamase, and ligand-bound complexes of these protein targets [Data set]. Zenodo. 10.5281/zenodo.15530247. Deposited 27 May 2025.

[101] N. Vlahakis, ED-MS MicroED: Single crystal X-ray diffraction datasets acquired from papain and papain-ligand comlexes [Data set]. Zenodo. 10.5281/zenodo.14876819. Deposited 16 February 2025.

[102] N. Vlahakis, J. Summers, D. de Sanctis, S. Wakatsuki, J. A. Rodriguez, Serial synchrotron X-ray diffraction datasets of papain microcrystals soaked with E-64 and analogs. ESRF Data Portal. https://doi.esrf.fr/10.15151/ESRF-DC-2206265994. Deposited 7 July 2025.

[103] N. Vlahakis, ED-MS MicroED: supplementary MicroED, single crystal XRD, and serial synchrotron XRD structures of papain crystals soaked with natural product ligands [Data set]. Zenodo. 10.5281/zenodo.14885217. Deposited 18 February 2025.

[104] N. Vlahakis, ED-MS MicroED: supplementary single crystal XRD structures of papain crystals soaked with mixtures of natural product ligands [Data set]. Zenodo. 10.5281/zenodo.15530411. Deposited 27 May 2025.

[105] C. Flowers, ED-MS nMS: main and supplementary raw spectra files [Data set]. Zenodo. https://zenodo.org/records/15522990. Deposited 27 May 2025.

