# Supplementary material for: Combining MicroED and native mass spectrometry for structural discovery of enzyme–small molecule complexes
Source: Proc Natl Acad Sci U S A. 2025 Jul 28;122(31):e2503780122. doi: 10.1073/pnas.2503780122 (PMC12337315; doi:10.1073/pnas.2503780122)
Supplement: Supplementary file 1 — Appendix 01 (PDF) [file pnas.2503780122.sapp.pdf]

Supporting Information for

Combining MicroED and native mass spectrometry for structural discovery of enzyme-small molecule complexes

Niko W. Vlahakis[1*], Cameron W. Flowers[1], Mengting Liu[2], Matthew Agdanowski[1], Samuel Johnson[3], Jacob A. Summers[4,5], Lian M. C. Jacobs[6], Catherine Keyser[1], Phoebe Russell[1], Samuel L. Rose[7], Julien Orlans[7], Nima Adhami[1], Yu Chen[1], Michael R. Sawaya[1], Shibom Basu[8], Daniele de Sanctis[7], Yu Chen[6], Soichi Wakatsuki[4,5], Hosea M. Nelson[3], Joseph A. Loo[1], Yi Tang[2], and Jose A. Rodriguez[1*]

[1] Department of Chemistry and Biochemistry; UCLA-DOE Institute for Genomics and Proteomics; STROBE, NSF Science and Technology Center; University of California, Los Angeles (UCLA); Los Angeles, CA 90095, USA.

[2] Department of Chemical and Biomolecular Engineering; University of California, Los Angeles (UCLA); Los Angeles, CA 90095, USA.

[3] Division of Chemistry and Chemical Engineering, California Institute of Technology, Pasadena, California, 91125, USA

[4] Department of Structural Biology, Stanford University School of Medicine; Stanford, CA 94304, USA.

[5] Biological Sciences Division, SLAC National Accelerator Laboratory, Menlo Park, CA 94025, USA.

[6] Department of Molecular Medicine, Morsani College of Medicine, University of South Florida, Tampa, FL 33612, USA.

[7] ESRF – The European Synchrotron, 71 Avenue des Martyrs, Grenoble, 38000, France.

[8] European Molecular Biology Laboratory, 71 Avenue des Martyrs, Grenoble, 38000, France.

*Correspondence to: *Niko W. Vlahakis (nwvlahakis@g.ucla.edu) and Jose A. Rodriguez (jrodriguez@mbi.ucla.edu)*

This PDF file includes:

Figures S1 to S23

Tables S1 to S19

CheckCIF structure validation report and response

Other supporting materials for this manuscript include the following:

Datasets deposited on *Zenodo*

Structures deposited to the PDB and CCDC

PDB structure validation reports

Supplementary Tables

Table S1. Statistics of crystallographic data reduction and refinement for MicroED structures of apo-form lysozyme, lysozyme co-crystallized with N,N',N"-triacetylchitotriose (ACT), lysozyme soaked with ACT, and lysozyme soaked with inhibitor cocktail including ACT.

	Lysozyme (apo)	Lysozyme (ACT cocrystal)	Lysozyme (ACT-soaked)	Lysozyme (ACT/cocktail soaked)
**PDB ID**	9ORZ	9OS1	9OS8	9OS0
**Data Collection and Processing**				
No. crystals merged	1	1	1	1
Temperature (K)	100	100	100	100
Electron wavelength (Å)	0.0251	0.0251	0.0251	0.0251
Resolution (Å)	54.82 – 2.30 (2.40-2.30)	34.50 – 2.30 (2.40-2.30)	54.50 – 2.30 (2.40-2.30)	54.21 – 2.40 (2.50-2.40)
Space Group	$P4_32_12$	$P4_32_12$	$P4_32_12$	$P4_32_12$
a, *b*, *c* (Å)	77.53, 77.53, 37.33	77.15, 77.15, 37.49	77.08, 77.08, 38.05	76.99, 76.99, 38.22
α, *β*, *γ* (°)	90, 90, 90	90, 90, 90	90, 90, 90	90, 90, 90
# total reflections	30058 (3642)	40130 (4949)	30229 (3569)	24994 (2927)
# unique reflections	5060 (603)	4738 (562)	4648 (536)	4019 (453)
R_{merge} (%)	25.2 (103.2)	28.3 (122.2)	29.3 (115.9)	29.0 (107.0)
CC1/2 (%)	98.0 (52.3)	98.1 (44.7)	96.3 (39.3)	97.7 (43.8)
$\langle I/\sigma I \rangle$	6.01 (2.22)	6.47 (2.31)	5.21 (1.99)	5.47 (2.04)
Completeness (%)	92.9 (95.6)	87.5 (88.6)	84.9 (84.8)	83.6 (86.5)
**Phasing**				
Search model PDB	1DPX	1DPX	1DPX	1DPX
**Refinement**				
Resolution (Å)	54.82 – 2.30	34.50 – 2.30	54.50 – 2.30	54.21 – 2.40
R_{work} (%)	20.58	22.46	22.35	20.46

R_{free} (%)	24.97	26.37	26.89	25.55
# protein atoms	1001	1005	1005	1003
# ligand/ion atoms	1	44	43	44
# solvent atoms	15	4	1	4
Occupancy of ligand atoms (%)	N/A	100	73	97
Average B-factor (Å^2)				
Protein	26.30	32.37	22.29	21.78
Ligand/ion	27.36	40.19	31.45	30.39
Solvent	20.28	22.03	8.00	11.46
Ramachandran (%)				
Favored (%)	97.64	96.06	97.64	96.85
Allowed (%)	2.36	3.94	2.36	3.15
Outliers (%)	0.00	0.00	0.00	0.00
R.M.S. deviations				
Bond lengths (Å)	0.009	0.007	0.006	0.010
Bond angles (°)	1.225	1.231	1.133	1.397
Clashscore	6.12	9.44	6.96	6.47

Table S2. Statistics of crystallographic data reduction and refinement for single-crystal XRD structures of apo-form lysozyme, lysozyme co-crystallized with N,N',N"-triacetylchitotriose (ACT), lysozyme soaked with ACT, and lysozyme soaked with inhibitor cocktail including ACT.

	Lysozyme (apo)	Lysozyme (ACT cocrystal)	Lysozyme (ACT--soaked)	Lysozyme (ACT/cocktail- soaked)
**PDB ID**	9ORW	9ORV	9ORY	9ORX
**Data Collection and Processing**				
Temperature (K)	100	100	100	100
Electron wavelength (Å)	0.0251	0.0251	0.0251	0.0251
Resolution (Å)	54.38 – 1.50 (1.55-1.50)	38.77 – 1.60 (1.70-1.60)	54.26 – 1.40 (1.50-1.40)	27.41 – 1.50 (1.60-1.50)
Space Group	$P4_32_12$	$P4_32_12$	$P4_32_12$	$P4_32_12$
a, *b*, *c* (Å)	76.90, 76.90, 37.15	77.53, 77.53, 37.89	76.74, 76.74, 37.75	77.53, 77.53, 37.80
α, *β*, *γ* (°)	90, 90, 90	90, 90, 90	90, 90, 90	90, 90, 90
# total reflections	464548 (79950)	209654 (33808)	379777 (37415)	497680 (85756)
# unique reflections	34094 (6013)	29129 (4827)	42165 (7695)	35260 (6213)
R_{merge} (%)	4.1 (18.8)	6.3 (49.6)	4.2 (27.1)	5.3 (139.7)
CC1/2 (%)	100.0 (99.5)	99.9 (87.9)	100.0 (97.0)	100.0 (49.7)
$\langle I/\sigma I \rangle$	38.90 (13.10)	17.39 (3.67)	27.35 (3.85)	25.42 (1.88)
Completeness (%)	100.0 (100.0)	99.9 (100.0)	99.4 (97.2)	99.8 (100.0)
**Phasing**				
Search model PDB	193L	193L	193L	1DPX
**Refinement**				
Resolution (Å)	54.38 – 1.50	38.77 – 1.60	54.26 – 1.40	27.41 – 1.50
R_{work} (%)	18.18	18.84	18.93	20.67
R_{free} (%)	21.84	21.00	21.54	23.37
# protein atoms	1035	1040	1040	1005
# ligand/ion atoms	3	46	46	44
# solvent atoms	110	99	117	70

Occupancy of ligand atoms (%)	N/A	86	89	74
Average B-factor ($Å^2$)				
Protein	26.58	22.57	19.64	29.45
Ligand/ion	20.21	28.05	27.47	39.13
Solvent	23.17	32.84	28.85	33.34
Ramachandran (%)				
Favored (%)	98.43	98.43	98.43	97.64
Allowed (%)	1.57	1.57	1.57	2.36
Outliers (%)	0.00	0.00	0.00	0.00
R.M.S. deviations				
Bond lengths (Å)	0.008	0.007	0.005	0.008
Bond angles (º)	1.023	0.860	0.828	0.988
Clashscore	3.93	3.35	3.35	2.48

Table S3. Statistics of crystallographic data reduction and refinement for MicroED structures of apo-form papain, papain co-crystallized with E-64, and papain co-crystallized with E-64-A65.

	Papain (apo)	Papain-E-64	Papain-E-64-A65
**PDB ID**	9NAG	9NAE	9NAO
**Data Collection and Processing**			
No. crystals merged	2	1	1
Temperature (K)	100	100	100
Electron wavelength (Å)	0.0251	0.0251	0.0251
Resolution (Å)	50.08 – 2.50 (2.60-2.50)	50.34- 2.30 (2.40-2.30)	50.24 – 2.50 (2.60 – 2.50)
Space Group	$P2_12_12_1$	$P2_12_12_1$	$P2_12_12_1$
a, *b*, *c* (Å)	41.99, 49.09, 100.15	42.18, 49.25, 100.69	42.31, 48.32, 100.48
α, *β*, *γ* (°)	90,90,90	90,90,90	90,90,90
# total reflections	33883 (3776)	30859 (3725)	24784 (2817)
# unique reflections	6918 (759)	9018 (1062)	6665 (742)
R_{merge} (%)	29.5 (47.4)	25.3 (103.6)	24.9 (103.4)
CC1/2 (%)	94.6 (42.8)	96.6 (41.2)	96.2 (45.5)
$\langle I/\sigma I \rangle$	5.48 (3.62)	3.84 (1.53)	4.27 (1.36)
Completeness (%)	90.5 (91.1)	91.4 (92.6)	87.7 (91.2)
**Phasing**			
Search model PDB	9PAP	9PAP	9PAP
**Refinement**			
Resolution (Å)	50.08 – 2.80	50.34 – 2.30	50.24 – 2.50
R_{work} (%)	21.28	20.31	23.35
R_{free} (%)	24.78	25.70	26.46
# protein atoms	1720	1699	1717
# ligand atoms	0	25	26
# solvent atoms	13	22	9
Occupancy of ligand atoms (%)	N/A	73	67

Average B-factor (Å^2)			
Protein	28.34	23.84	31.14
Ligand	N/A	30.62	37.44
Solvent	10.95	19.17	18.09
Ramachandran (%)			
Favored (%)	97.62	98.10	97.14
Allowed (%)	2.38	1.90	2.86
Outliers (%)	0.00	0.00	0.00
R.M.S. deviations			
Bond lengths (Å)	0.007	0.007	0.007
Bond angles (°)	0.999	0.959	1.044
Clashscore	5.35	4.87	8.55

Table S4. Statistics of crystallographic data reduction and refinement for MicroED structures of papain soaked with E-64 for 30 seconds, 4 minutes, and 10 minutes. Structures marked with * did not feature adequately clear density to model a ligand, so the refinement statistics reported reference the model without a ligand modeled. The structure corresponding to the longest soaking time was deposited in the PDB.

	Papain-E-64 (30 seconds)*	Papain-E-64 (4 minutes)	Papain-E-64 (10 minutes)
**PDB ID**	N/A	N/A	9NAR
**Data Collection and Processing**			
No. crystals merged	1	2	2
Temperature (K)	100	100	100
Electron wavelength (Å)	0.0251	0.0251	0.0251
Resolution (Å)	26.85 – 2.50 (2.60 -2.50)	49.98 – 2.20 (2.30 – 2.20)	50.12 – 2.50 (2.60 – 2.50)
Space Group	$P2_12_12_1$	$P2_12_12_1$	$P2_12_12_1$
a, *b*, *c* (Å)	41.28, 49.36, 101.28	42.00, 49.39, 99.95	41.92, 48.32, 100.24
α, *β*, *γ* (°)	90,90,90	90,90,90	90,90,90
# total reflections	41792 (4814)	70608 (8476)	41982 (4636)
# unique reflections	6708 (732)	11081 (1335)	7342 (790)
R_{merge} (%)	33.2 (56.0)	31.3 (83.6)	29.6 (112.8)
CC1/2 (%)	96.7 (29.0)	97.7 (55.6)	97.1 (55.4)
$\langle I/\sigma I \rangle$	5.44 (3.41)	5.76 (2.49)	6.31 (2.19)
Completeness (%)	87.9 (88.5)	99.4 (97.9)	97.7 (98.5)
**Phasing**			
Search model PDB	9PAP	9PAP	9PAP
**Refinement**			
Resolution (Å)	26.85 – 2.50	49.98 – 2.50	50.12 – 2.50
R_{work} (%)	23.09	18.76	19.24
R_{free} (%)	27.10	24.64	20.59
# protein atoms	1716	1711	1680
# ligand atoms	0	25	25

# solvent atoms	6	41	31
Occupancy of ligand atoms (%)	N/A	72	77
Average B-factor (Å^2)			
Protein	14.57	18.77	21.80
Ligand	N/A	29.45	23.98
Solvent	3.38	12.42	12.74
Ramachandran (%)			
Favored (%)	97.14	98.10	97.62
Allowed (%)	2.86	1.90	2.38
Outliers (%)	0.00	0.00	0.00
R.M.S. deviations			
Bond lengths (Å)	0.011	0.008	0.007
Bond angles (°)	1.382	0.971	0.935
Clashscore	11	6.18	5.08

Table S5. Model-map correlations for soaking-time series MicroED structures of papain soaked with E-64

	Papain-E-64 (30 seconds)*	Papain-E-64 (4 minutes)	Papain-E-64 (10 minutes)
**Overall CC (no ligand modeled)**	0.84	0.87	0.85
**Overall CC (ligand modeled)**	0.84	0.90	0.86
**Local CC (no ligand modeled)**	0.88	0.87	0.89
**Local CC (ligand modeled)**	0.88	0.89	0.89

Table S6. Statistics of crystallographic data reduction and refinement for single-crystal XRD structures of papain soaked with E-64 for 30 seconds, 4 minutes, 10 minutes, 80 minutes, and 72 hours. Structures marked with * did not feature adequately clear density to model a ligand, so the refinement statistics reported reference the model without a ligand modeled. The structure corresponding to the longest soaking time was deposited in the PDB.

	Papain-E-64 (30 seconds)*	Papain-E-64 (4 minutes)*	Papain-E-64 (10 minutes)*	Papain-E-64 (80 minutes)	Papain-E-64 (72 hours)
**PDB ID**	N/A	N/A	N/A	N/A	9NB2
**Data Collection and Processing**					
X-ray wavelength (Å)	1.54	1.54	1.54	1.54	1.54
Temperature (K)	100	100	100	100	100
Resolution (Å)	50.80 – 1.50 (1.60 – 1.50)	50.81 – 1.50 (1.60 – 1.50)	39.21 – 1.50 (1.60 – 1.50)	44.23 – 1.60 (1.70 – 1.60)	27.86 – 1.50 (1.60 – 1.50)
Space Group	$P2_12_12_1$	$P2_12_12_1$	$P2_12_12_1$	$P2_12_12_1$	$P2_12_12_1$
a, *b*, *c* (Å)	42.65, 49.18, 101.61	42.57, 49.10, 101.62	42.51, 49.27, 101.54	42.57, 49.15 101.38	42.41, 48.91, 101.71
α, *β*, *γ* (°)	90,90,90	90,90,90	90,90,90	90,90,90	90,90,90
# total reflections	440990 (77330)	442445 (76714)	458274 (78829)	355576 (63574)	417970 (71570)
# unique reflections	34702 (5895)	34861 (6005)	34970 (6033)	27776 (4456)	34540 (6000)
R_{merge} (%)	11.3 (99.2)	15.1 (83.3)	7.2 (55.0)	5.6 (36.4)	7.8 (71.0)
CC1/2 (%)	99.8 (89.5)	99.6 (67.0)	99.9 (92.1)	99.9 (96.7)	99.9 (85.0)
$\langle I/\sigma I \rangle$	12.53 (2.95)	11.29 (3.37)	20.53 (4.93)	26.44 (6.13)	18.22 (3.72)
Completeness (%)	98.8 (97.0)	99.8 (99.3)	99.8 (99.6)	96.1 (94.1)	99.4 (100.0)
**Phasing**					
Search model PDB	9PAP	9PAP	9PAP	9PAP	9PAP
**Refinement**					
Resolution (Å)	50.80 – 1.50	50.80 – 1.50	39.21 – 1.50	44.23 – 1.60	27.86 – 1.50
R_{work} (%)	19.71	19.33	18.95	22.00	18.35
R_{free} (%)	22.30	21.53	20.75	26.51	21.24
# protein atoms	1721	1703	1706	1699	1725
# ligand atoms	0	0	0	25	25

# solvent atoms	213	242	283	211	230
Occupancy of ligand atoms (%)	N/A	N/A	N/A	61	82
Average B-factor (Å^2)					
Protein	20.34	17.36	18.29	19.63	18.41
Ligand	N/A	N/A	N/A	24.14	28.96
Solvent	29.18	26.48	29.71	27.38	26.95
Ramachandran (%)					
Favored (%)	98.07	98.55	98.07	98.10	98.57
Allowed (%)	1.93	1.45	1.93	1.90	1.43
Outliers (%)	0.00	0.00	0.00	0.00	0.00
R.M.S. deviations					
Bond lengths (Å)	0.006	0.006	0.006	0.007	0.006
Bond angles (º)	0.898	0.892	0.857	0.898	0.838
Clashscore	1.49	1.80	2.10	1.48	2.33

Table S7. Expected molecular weights of target enzymes and ligands referenced for nMS analysis. Entries in rows indicating protein masses are bolded for clarity.

**Species**	**Average mass (Da)**	**Protein + ligand mass (Da)**
**Papain(Reduced C25)**	**23422.9**	**N/A**
**Papain(Sulfinic C25)**	**23454.89**	**N/A**
E-64	357.2	23780.1
E-64-A65	369.23	23792.13
E-64C	314.38	23737.28
E-64D	342.44	23765.34
Leupeptin	426.6	23849.5
E315	315.18	23738.08
E371	371.21	23794.11
E405	405.19	23828.09
**CTX-M-14 β-lactamase**	**27956**	**NA**
Avibactam	265.24	28221.24
**Hen egg-white lysozyme**	**14304**	**N/A**
N,N',N"-triacetylchitotriose (ACT)	627.60	14931.6

Table S8. Statistics of crystallographic data reduction and refinement for MicroED structures of papain soaked with ligand mixtures. For structures marked with *, active-site ligand density was not sufficiently clear for confident ligand modeling, so ligands were not included in refinement for these structures and they were not deposited in the PDB.

	Papain-protease inhibitor cocktail	Papain-E-64,E-64C, and E-64D mixture	Papain-E-64-A65, E315, E372, and E405 mixture*	Papain-E315, E372, and E405 mixture*
**PDB ID**	9NC1	9NCA	N/A	N/A
**Data Collection and Processing**				
No. crystals merged	2	3	1	1
Temperature (K)	100	100	100	100
Electron wavelength (Å)	0.0251	0.0251	0.0251	0.0251
Resolution (Å)	32.06 – 2.40 (2.50 – 2.40)	49.82 – 2.50 (2.60 – 2.50)	19.86 – 2.40 (2.50 – 2.40)	31.86 – 2.50 (2.60 – 2.50)
Space Group	$P2_12_12_1$	$P2_12_12_1$	$P2_12_12_1$	$P2_12_12_1$
a, *b*, *c* (Å)	42.57, 48.85, 99.86	42.74, 48.96, 99.64	42.22, 49.15, 101.25	41.83, 49.18, 99.99
α, *β*, *γ* (°)	90,90,90	90,90,90	90,90,90	90,90,90
# total reflections	36267 (4240)	49787 (5215)	34102 (4020)	26915 (3040)
# unique reflections	8231 (940)	6282 (638)	7088 (806)	6628 (733)
R_{merge} (%)	24.6 (67.2)	33.5 (120.7)	38.2 (126.0)	26.4 (95.6)
CC1/2 (%)	96.7 (58.8)	86.7 (62.3)	95.9 (42.0)	95.7 (35.5)
<I/σI>	5.31 (2.63)	6.12 (2.45)	4.69 (1.90)	5.03 (1.79)
Completeness (%)	95.2 (96.7)	81.5 (76.9)	81.3 (81.3)	87.0 (87.8)
**Phasing**				
Search model PDB	9PAP	9PAP	9PAP	9PAP
**Refinement**				
Resolution (Å)	32.06 – 2.40	49.82 – 2.50	19.86 – 2.40	31.86 – 2.50
R_{work} (%)	20.33	22.16	23.65	20.75
R_{free} (%)	26.09	27.56	28.27	26.07
# protein atoms	1655	1681	1674	1695
# ligand atoms	12	47	0	0

# solvent atoms	25	2	13	13
Occupancy of ligand atoms (%)	76	52 (E-64), 42 (E-64C)	N/A	N/A
Average B-factor (Å^2)				
Protein	22.83	18.10	27.65	25.46
Ligand	29.16	38.04	N/A	N/A
Solvent	14.70	2.83	24.10	13.22
Ramachandran (%)				
Favored (%)	97.62	98.10	97.14	96.67
Allowed (%)	2.38	1.90	2.86	3.33
Outliers (%)	0.00	0.00	0.00	0.00
R.M.S. deviations				
Bond lengths (Å)	0.006	0.009	0.013	0.011
Bond angles (º)	0.914	1.311	1.606	1.472
Clashscore	2.43	10.68	8.55	9.33

Table S9. Statistics of crystallographic data reduction and refinement for single-crystal XRD structure of papain soaked with ligand mixtures for 20 hours. Active-site ligand density was not sufficiently clear for confident ligand modeling, so ligands were not included in refinement for these structures and they were not deposited in the PDB.

	Papain-protease inhibitor cocktail (XRD)	Papain-E-64,E-64C, and E-64D mixture	Papain-E-64-A65, E315, E372, and E405 mixture
**PDB ID**	N/A	N/A	N/A
**Data Collection and Processing**			
X-ray wavelength (Å)	1.54	1.54	1.54
Temperature (K)	100	100	100
Resolution (Å)	44.03 – 1.50 (1.60 – 1.50)	51.24 – 1.40 (1.50 – 1.40)	47.98 – 2.00 (2.10 – 2.00)
Space Group	$P2_12_12_1$	$P2_12_12_1$	$P2_12_12_1$
a, *b*, *c* (Å)	42.37, 48.87, 101.49	42.44, 49.12, 102.49	42.26, 45.57, 95.97
α, *β*, *γ* (°)	90,90,90	90,90,90	90,90,90
# total reflections	459015 (78661)	525721 (71913)	172797 (22983)
# unique reflections	34593 (5971)	42660 (7496)	24177 (3275)
R_{merge} (%)	11.8 (72.0)	8.7 (62.9)	14.8 (119.5)
CC1/2 (%)	99.7 (89.7)	99.9 (20.6)	99.7 (67.4)
<I/σI>	13.84 (3.42)	15.22 (2.15)	8.95 (1.42)
Completeness (%)	100.0 (100.0)	99.0 (94.7)	99.8 (100.0)
**Phasing**			
Search model PDB	9PAP	9PAP	9PAP
**Refinement**			
Resolution (Å)	44.03 – 1.50	51.24 – 1.40	47.98 – 2.00
R_{work} (%)	21.41	21.39	20.44
R_{free} (%)	23.68	23.53	26.66
# protein atoms	1652	1655	1667
# ligand atoms	0	0	0
# solvent atoms	108	107	55
Occupancy of ligand atoms (%)	N/A	N/A	N/A

Average B-factor (Å^2)			
Protein	20.76	22.77	34.33
Ligand	N/A	N/A	N/A
Solvent	24.36	26.09	31.91
Ramachandran (%)			
Favored (%)	97.62	97.14	98.57
Allowed (%)	2.38	2.86	1.43
Outliers (%)	0.00	0.00	0.00
R.M.S. deviations			
Bond lengths (Å)	0.006	0.007	0.007
Bond angles (º)	1.047	0.938	0.824
Clashscore	2.47	3.69	4.89

Table S10. Statistics of crystallographic data reduction and refinement for single-crystal XRD structure of papain co-crystallized with leupeptin.

	Papain-leupeptin (XRD)
**PDB ID**	9NAT
**Data Collection and Processing**	
X-ray/electron wavelength (Å)	1.54
Temperature (K)	100
Resolution (Å)	39.11 – 1.60 (1.70 – 1.60)
Space Group	$P2_12_12_1$
a, *b*, *c* (Å)	42.45, 48.91, 100.80
α, *β*, *γ* (°)	90,90,90
# total reflections	380919 (62121)
# unique reflections	28509 (4665)
R_{merge} (%)	6.5 (16.9)
CC1/2 (%)	99.9 (99.4)
<I/σI>	26.69 (11.90)
Completeness (%)	100 (100)
**Phasing**	
Search model PDB	9PAP
**Refinement**	
Resolution (Å)	39.11 – 1.60
R_{work} (%)	16.92
R_{free} (%)	19.76
# protein atoms	1708
# ligand atoms	30
# solvent atoms	267
Occupancy of ligand atoms (%)	75
Average B-factor (Å^2)	

Protein	15.44
Ligand	28.28
Solvent	24.87
Ramachandran (%)	
Favored (%)	98.10
Allowed (%)	1.90
Outliers (%)	0.00
R.M.S. deviations	
Bond lengths (Å)	0.006
Bond angles (º)	0.936
Clashscore	1.48

Table S11. Statistics of crystallographic data reduction and refinement for serial synchrotron X-ray diffraction structures of apo-form papain, papain soaked with E-64, papain soaked with E-64C, papain soaked with E-64D, and papain soaked with an equimolar mixture of all three ligands. For the mixture soaking experiment, the common structural fragment between the three potential ligands was modeled in the structure and refined.

	Papain (apo)	Papain-E-64	Papain-E-64C	Papain-E-64D	Papain-E-64,E-64C,E-64D mixture
**PDB ID**	9NCC	9NBF	9NBJ	9NBK	9NBN
**Data Collection and Processing**					
X-ray wavelength (Å)	1.072	1.072	1.072	1.072	1.073
Temperature (K)	293	293	293	293	293
# hits	9705	7857	22890	41210	23328
# indexed images	7284	6390	18301	33457	15005
# crystals indexed	8201	6921	19985	36673	16147
Resolution (Å)	44.05 – 1.80 (1.83 – 1.80)	44.05 – 1.80 (1.83 – 1.80)	44.05 – 1.80 (1.83 – 1.80)	44.05 – 1.80 (1.83 – 1.80)	44.05 – 1.80 (1.83 – 1.80)
Space Group	$P2_12_12_1$	$P2_12_12_1$	$P2_12_12_1$	$P2_12_12_1$	$P2_12_12_1$
a, *b*, *c* (Å)	42.5, 48.9, 101.5	42.5, 48.9, 101.5	42.5, 48.9, 101.5	42.5, 48.9, 101.5	42.5, 48.9, 101.5
α, *β*, *γ* (°)	90,90,90	90,90,90	90,90,90	90,90,90	90,90,90
# total reflections	3195456 (109871)	2591698 (90854)	9961279 (349481)	18939476 (661124)	4617351 (162646)
# unique reflections	37921 (1877)	37298 (1877)	37928 (1877)	37928 (1877)	37928 (1877)
R_{split} (%)	31.7 (212.7)	37.5 (239.5)	21.3 (86.5)	15.4 (58.9)	30.4 (239.2)
CC*(%)	96.7 (46.9)	94.7 (39.3)	98.2 (76.3)	99.1 (86.4)	97.3 (39.5)
<I/σI>	2.6 (0.5)	2.3 (0.5)	4.3 (1.2)	6.1 (1.8)	2.6 (0.5)
Completeness (%)	100 (100)	100 (100)	100 (100)	100 (100)	100 (100)
**Phasing**					
Search model PDB	9PAP	9PAP	9PAP	9PAP	9PAP
**Refinement**					
Resolution (Å)	44.05 – 1.80	44.05 – 1.80	44.05 – 1.80	44.05 – 1.80	44.05 – 1.80
R_{work} (%)	19.75	20.23	17.00	15.89	19.40

R_{free} (%)	23.25	23.90	21.00	19.75	23.77
# protein atoms	1712	1714	1698	1709	1683
# ligand atoms	0	25	22	24	17
# solvent atoms	59	87	102	106	77
Occupancy of ligand atoms (%)	N/A	60	58	65	73
Average B-factor (Å^2)					
Protein	32.43	33.30	32.22	32.11	35.34
Ligand	N/A	39.80	42.11	44.31	42.68
Solvent	34.20	37.18	38.09	39.52	37.66
Ramachandran (%)					
Favored (%)	97.14	97.14	97.14	97.14	97.62
Allowed (%)	2.86	2.86	2.86	2.86	2.38
Outliers (%)	0.00	0.00	0.00	0.00	0.00
R.M.S. deviations					
Bond lengths (Å)	0.008	0.007	0.007	0.008	0.008
Bond angles (°)	0.983	0.939	0.958	0.954	0.959
Clashscore	4.47	4.69	4.15	3.81	3.31

Table S12. Statistics of crystallographic data reduction and refinement for MicroED structures of papain co-crystallized with E-64C and E-64D.

	Papain-E-64C	Papain-E-64D
**PDB ID**	9N9D	9NBQ
**Data Collection and Processing**		
No. crystals merged	1	1
Temperature (K)	100	100
Electron wavelength (Å)	0.0251	0.0251
Resolution (Å)	50.46 – 2.20 (2.30 – 2.20)	32.32 – 2.30 (2.40 – 2.30)
Space Group	$P2_12_12_1$	$P2_12_12_1$
a, *b*, *c* (Å)	41.88, 48.93, 100.93	42.08, 49.34, 100.92
α, *β*, *γ* (°)	90,90,90	90,90,90
# total reflections	43354 (5189)	37992 (4444)
# unique reflections	10957 (1314)	9338 (1076)
R_{merge} (%)	26.8 (98.8)	34.4 (94.6)
CC1/2 (%)	97.0 (45.9)	94.3 (47.4)
<I/σI>	5.29 (2.00)	4.68 (2.04)
Completeness (%)	98.7 (97.8)	94.5 (93.6)
**Phasing**		
Search model PDB	9PAP	9PAP
**Refinement**		
Resolution (Å)	50.46 – 2.20	32.32 – 2.30
R_{work} (%)	18.12	19.33
R_{free} (%)	23.63	24.70
# protein atoms	1729	1724
# ligand atoms	41	24
# solvent atoms	23.25	33
Occupancy of ligand atoms (%)	81	74

Average B-factor ($Å^2$)		
Protein	23.00	19.29
Ligand	39.02	43.79
Solvent	17.46	12.32
Ramachandran (%)		
Favored (%)	97.14	97.62
Allowed (%)	2.86	2.38
Outliers (%)	0.00	0.00
R.M.S. deviations		
Bond lengths (Å)	0.009	0.007
Bond angles (º)	1.327	0.958
Clashscore	4.36	6.41

Table S13. Statistics of crystallographic data reduction and refinement for MicroED structure of E-64D

	E-64D
**CCDC number**	2423833
**Data Collection and Processing**	
No. crystals merged	1
Electron wavelength (Å)	0.0251
Temperature (K)	100
Resolution (Å)	12.08 – 0.8 (0.9 – 0.8)
Space Group	$P2_12_12_1$
a, *b*, *c* (Å)	4.72, 13.31, 28.78
α, *β*, *γ* (°)	90,90,90
# total reflections	8127 (2218)
# unique reflections	1992 (551)
R_{merge} (%)	11.7 (34.9)
CC1/2 (%)	99.3 (76.9)
<I/σI>	7.02 (2.85)
Completeness (%)	90.0 (89.4)
**Phasing**	
Software	*SHELXT*
**Refinement**	
Resolution (Å)	12.08 – 0.8
R_1 (%)	15.75
wR_2 (%)	40.95
Goodness of fit	1.400

Table S14. Statistics of crystallographic data reduction and refinement for MicroED structure of papain from microcrystals mixed on-grid with arrayed E-64.

	Papain-E-64 (array sample preparation)
**PDB ID**	9NBP
**Data Collection and Processing**	
No. crystals merged	2
Temperature (K)	100
Electron wavelength (Å)	0.0251
Resolution (Å)	50.22 – 2.80 (2.90 – 2.80)
Space Group	$P2_12_12_1$
a, *b*, *c* (Å)	41.70, 49.28, 100.44
α, *β*, *γ* (°)	90,90,90
# total reflections	25374 (2426)
# unique reflections	4950 (471)
R_{merge} (%)	37.6 (95.8)
CC1/2 (%)	94.8 (62.3)
<I/σI>	4.74 (2.02)
Completeness (%)	90.1 (89.9)
**Phasing**	
Search model PDB	9PAP
**Refinement**	
Resolution (Å)	50.22 – 2.80
R_{work} (%)	19.79
R_{free} (%)	24.61
# protein atoms	1682
# ligand atoms	10
# solvent atoms	25
Occupancy of ligand atoms (%)	75
Average B-factor ($Å^2$)	
Protein	19.82
Ligand	26.53

Solvent	7.15
Ramachandran (%)	
Favored (%)	97.62
Allowed (%)	2.38
Outliers (%)	0.00
R.M.S. deviations	
Bond lengths (Å)	0.007
Bond angles (º)	1.014
Clashscore	7.47

Table S15. Statistics of crystallographic data reduction and refinement for serial synchrotron X-ray diffraction structures of papain soaked with E-64-A65, papain soaked with E315, papain soaked with E371, papain soaked with E405, and papain soaked with an equimolar mixture of all four natural product compounds. For structures marked with *, active-site ligand density was not sufficiently clear for confident ligand modeling, so ligands were not included in refinement for these structures and they were not deposited in the PDB.

	Papain-E-64-A65	Papain-E315*	Papain-371*	Papain-E405	Papain-E-64-A65,E315,E371,E405 mixture*
**PDB ID**	9NB4	N/A	N/A	9NB7	N/A
**Data Collection and Processing**					
X-ray wavelength (Å)	1.072	1.072	1.072	1.072	1.072
Temperature (K)	293	293	293	293	293
# hits	25777	37779	28180	27585	24241
# indexed images	22420	31349	20318	21818	20831
# crystals indexed	24052	33986	21621	23401	22368
Resolution (Å)	44.05 – 1.80 (1.83 – 1.80)	44.05 – 1.80 (1.83 – 1.80)	44.05 – 1.80 (1.83 – 1.80)	44.05 – 1.80 (1.83 – 1.80)	44.05 – 1.80 (1.83 – 1.80)
Space Group	$P2_12_12_1$	$P2_12_12_1$	$P2_12_12_1$	$P2_12_12_1$	$P2_12_12_1$
a, *b*, *c* (Å)	42.5, 48.9, 101.5	42.5, 48.9, 101.5	42.5, 48.9, 101.5	42.5, 48.9, 101.5	42.5, 48.9, 101.5
α, *β*, *γ* (°)	90,90,90	90,90,90	90,90,90	90,90,90	90,90,90
# total reflections	12627156 (441126)	16706172 (582261)	10335316 (359513)	11270147 (394517)	8021599 (281167)
# unique reflections	37928 (1877)	37928 (1877)	37928 (1877)	37928 (1877)	37928 (1877)
R_{split} (%)	18.6 (79.6)	16.6 (70.9)	19.2 (124.2)	20.6 (92.1)	23.3 (23.3)
CC* (%)	98.7 (77.0)	99.0 (80.6)	98.8 (64.4)	98.3 (75.4)	98.0 (74.6)
<I/σI>	4.9 (1.3)	5.6 (1.5)	4.2 (0.9)	4.6 (1.2)	3.9 (1.1)
Completeness (%)	100 (100)	100 (100)	100 (100)	100 (100	100 (100)
**Phasing**					
Search model PDB	9PAP	9PAP	9PAP	9PAP	9PAP

Refinement

Resolution (Å)	44.05 – 1.80	44.05 – 1.80	44.05 – 1.80	44.05 – 1.80	44.05 – 1.80
R_{work} (%)	16.67	16.29	17.48	17.39	19.33
R_{free} (%)	20.96	19.68	20.23	21.50	23.28
# protein atoms	1721	1700	1669	1706	1652
# ligand atoms	26	0	0	29	0
# solvent atoms	95	83	102	79	49
Occupancy of ligand atoms (%)	68	N/A	N/A	64	N/A
Average B-factor (Å^2)					
Protein	31.79	32.13	32.82	31.93	31.76
Ligand	41.74	N/A	N/A	43.33	N/A
Solvent	36.25	35.82	38.96	37.22	31.13
Ramachandran (%)					
Favored (%)	98.57	97.14	96.67	97.62	97.14
Allowed (%)	1.43	2.86	3.33	2.38	2.86
Outliers (%)	0.00	0.00	0.00	0.00	0.00
R.M.S. deviations					
Bond lengths (Å)	0.007	0.008	0.008	0.007	0.007
Bond angles (°)	0.965	0.936	0.985	1.041	0.881
Clashscore	1.77	5.06	5.50	2.37	4.01

Table S16. Statistics of crystallographic data reduction and refinement for MicroED structures of papain soaked with crude preparations of E-64 and E-64-A65

	Papain-E-64 (crude)	Papain-E-64-A65 (crude)
**PDB ID**	9NAX	9NAY
**Data Collection and Processing**		
No. crystals merged	2	2
Temperature (K)	100	100
Electron wavelength (Å)	0.0251	0.0251
Resolution (Å)	49.93 – 2.30 (2.40 – 2.30)	50.05 – 2.50 (2.60 – 2.50)
Space Group	$P2_12_12_1$	$P2_12_12_1$
a, *b*, *c* (Å)	41.85, 48.63, 99.87	42.20, 48.78, 100.10
α, *β*, *γ* (°)	90,90,90	90,90,90
# total reflections	69072 (7944)	40867 (4205)
# unique reflections	8337 (958)	6582 (688)
R_{merge} (%)	28.0 (88.2)	27.5 (93.7)
CC1/2 (%)	98.1 (66.3)	97.0 (55.4)
<I/σI>	6.46 (2.55)	5.46 (1.92)
Completeness (%)	87.0 (87.6)	86.4 (85.4)
**Phasing**		
Search model PDB	9PAP	9PAP
**Refinement**		
Resolution (Å)	49.93 – 2.30	50.05 – 2.50
R_{work} (%)	18.99	17.97
R_{free} (%)	24.75	22.77
# protein atoms	1666	1689
# ligand atoms	25	26
# solvent atoms	25	4

Occupancy of ligand atoms (%)	75	75
Average B-factor (Å^2)		
Protein	25.08	29.41
Ligand	31.38	41.61
Solvent	18.05	13.83
Ramachandran (%)		
Favored (%)	97.62	98.10
Allowed (%)	2.38	1.90
Outliers (%)	0.00	0.00
R.M.S. deviations		
Bond lengths (Å)	0.007	0.007
Bond angles (°)	0.964	1.119
Clashscore	5.13	7.50

Table S17. Statistics of crystallographic data reduction and refinement for MicroED structures of apo-form CTX-M-14 β-lactamase, CTX-M-14 β-lactamase co-crystallized with avibactam, CTX-M-14 β-lactamase soaked with avibactam, and soaked with inhibitor cocktail including avibactam.

	CTX-M-14 (apo)	CTX-M-14 (avibactam cocrystal)	CTX-M-14 (avibactam-soaked)	CTX-M-14 (avibactam/cocktail soaked)
**PDB ID**	9ORG	9ORS	9ORL	9ORH
**Data Collection and Processing**				
No. crystals merged	5	3	3	2
Temperature (K)	100	100	100	100
Electron wavelength (Å)	0.0251	0.0251	0.0251	0.0251
Resolution (Å)	43.99 – 2.50 (2.60-2.50)	46.81 – 2.00 (2.10-2.00)	46.63 – 2.30 (2.40-2.30)	27.65 – 2.00 (2.10-2.00)
Space Group	$P2_1$	$P2_1$	$P2_1$	$P2_1$
a, *b*, *c* (Å)	44.87, 107.27, 47.62	44.80, 105.79, 47.68	44.99, 105.70, 47.49	44.90, 106.42, 47.52
α, *β*, *γ* (°)	90,101.371,90	90, 100.957, 90	90, 100.951, 90	90, 100.689, 90
# total reflections	113750 (11763)	164695 (22732)	86854 (10503)	96106 (13339)
# unique reflections	13467 (1437)	24699 (3386)	18817 (2241)	28065 (3848)
R_{merge} (%)	38.7 (100.1)	29.1 (82.6)	26.8 (75.9)	25.4 (77.9)
CC1/2 (%)	94.5 (54.4)	96.1 (53.6)	97.0 (56.2)	96.1 (57.0)
<I/σI>	5.85 (2.78)	5.25 (2.52)	5.10 (2.37)	4.22 (2.01)
Completeness (%)	87.8 (86.8)	83.7 (84.4)	96.8 (96.1)	94.6 (95.6)
**Phasing**				
Search model PDB	1YLT	1YLT	1YLT	1YLT
**Refinement**				
Resolution (Å)	43.99 – 2.50	46.81 – 2.00	46.63 – 2.30	27.65 – 2.00
R_{work} (%)	20.88	20.29	17.16	19.74
R_{free} (%)	23.88	25.60	22.19	24.55
# protein atoms	3921	3931	3922	3950
# ligand atoms	N/A	34	34	34

# solvent atoms	12	32	27	38
Occupancy of ligand atoms (%)	N/A	93 (chain A), 82 (chain B)	81 (chain A), 85 (chain B)	81 (chain A), 84 (chain B)
Average B-factor (Å^2)				
Protein	18.81	21.01	20.42	20.62
Ligand	N/A	23.72	20.19	22.91
Solvent	14.52	15.65	12.64	15.30
Ramachandran (%)				
Favored (%)	98.07	97.88	97.50	97.50
Allowed (%)	1.54	1.73	1.92	2.12
Outliers (%)	0.39	0.38	0.58	0.38
R.M.S. deviations				
Bond lengths (Å)	0.004	0.005	0.005	0.008
Bond angles (º)	0.979	1.045	1.064	1.192
Clashscore	6.35	7.07	4.05	4.89

Table S18. Statistics of crystallographic data reduction and refinement for single-crystal XRD structures of apo-form CTX-M-14 β-lactamase, CTX-M-14 β-lactamase co-crystallized with avibactam, CTX-M-14 β-lactamase soaked with avibactam, and CTX-M-14 β-lactamase soaked with inhibitor cocktail including avibactam.

	CTX-M-14 (apo)	CTX-M-14 (avibactam cocrystal)	CTX-M-14 (avibactam-soaked)	CTX-M-14 (avibactam/cocktail soaked)
**PDB ID**	9OQE	9OR3	9OR7	9ORB
**Data Collection and Processing**				
Temperature (K)	100	100	100	100
X-ray wavelength (Å)	1.54	1.54	1.54	1.54
Resolution (Å)	46.61 – 1.50 (1.60-1.50)	53.40 – 1.60 (1.70-1.60)	46.94 – 1.50 (1.60-1.50)	35.29– 1.50 (1.60-1.50)
Space Group	$P2_1$	$P2_1$	$P2_1$	$P2_1$
a, *b*, *c* (Å)	45.18, 106.61, 47.71	45.04, 106.79, 47.59	45.29, 106.82, 47.98	45,21, 107.19, 7.92
α, *β*, *γ* (°)	90, 102.323, 90	90, 102.377, 90	90, 101.978, 90	90, 101.877, 90
# total reflections	463634 (82105)	413200 (66310)	484349 (82772)	492888 (83766)
# unique reflections	138574 (24441)	110382 (17859)	137873 (24033)	139145 (24375)
R_{merge} (%)	11.2 (58.6)	12.3 (63.1)	4.2 (23.3)	3.4 (7.7)
CC1/2 (%)	98.9 (73.0)	99.0 (66.0)	99.8 (95.1)	99.9 (99.2)
<I/σI>	9.10 (3.64	7.31 (2.24)	19.84 (6.62)	23.87 (12.71)
Completeness (%)	99.4 (99.9)	96.5 (94.0)	97.7 (97.0)	98.6 (98.2)
**Phasing**				
Search model PDB	1YLT	1YLT	1YLT	1YLT
**Refinement**				
Resolution (Å)	46.61 – 1.50	53.40 – 1.60	46.94 – 1.50	35.29 – 1.50
R_{work} (%)	14.77	15.53	18.71	15.23
R_{free} (%)	18.03	19.10	22.03	17.53
# protein atoms	4133	4076	4028	4030
# ligand/ion atoms	27	51	49	39
# solvent atoms	800	452	675	581

Occupancy of ligand atoms (%)	N/A	90 (chain A), 90 (chain B)	87 (chain A), 87 (chain B)	83 (chain A), 83 (chain B)
Average B-factor (Å^2)				
Protein	13.40	14.46	13.64	11.19
Ligand/ion	32.94	22.49	19.81	14.20
Solvent	26.02	25.83	23.20	22.18
Ramachandran (%)				
Favored (%)	97.88	97.69	98.27	97.88
Allowed (%)	1.73	1.92	1.35	1.73
Outliers (%)	0.38	0.38	0.38	0.38
R.M.S. deviations				
Bond lengths (Å)	0.006	0.007	0.006	0.006
Bond angles (º)	0.872	0.951	0.979	0.944
Clashscore	2.28	2.90	1.84	1.60

Table S19. Instrument tuning parameters for ion transmission.

**Instrument parameters**	
**Ion mode**	Positive
**Detector m/z Optimization**	Low m/z
**Source DC Offset (V)**	21
**In-source Trapping (V)**	Off
**Ion Transfer Target m/z**	Low m/z
**Injection Flatapole DC (V)**	5
**Inter Flatapole Lens (V)**	4
**Bent Flatapole DC (V)**	2
**Transfer Multipole DC (V)**	0
**C-Trap Entrance Lens Inject (V)**	2
**Trapping gas pressure setting**	3
**Nano ESI parameters**	
**spray Voltage(kV)**	0.6-1.4
**Capillary Temp (C°)**	200
**S-lens RF level**	200

Supplementary Figures

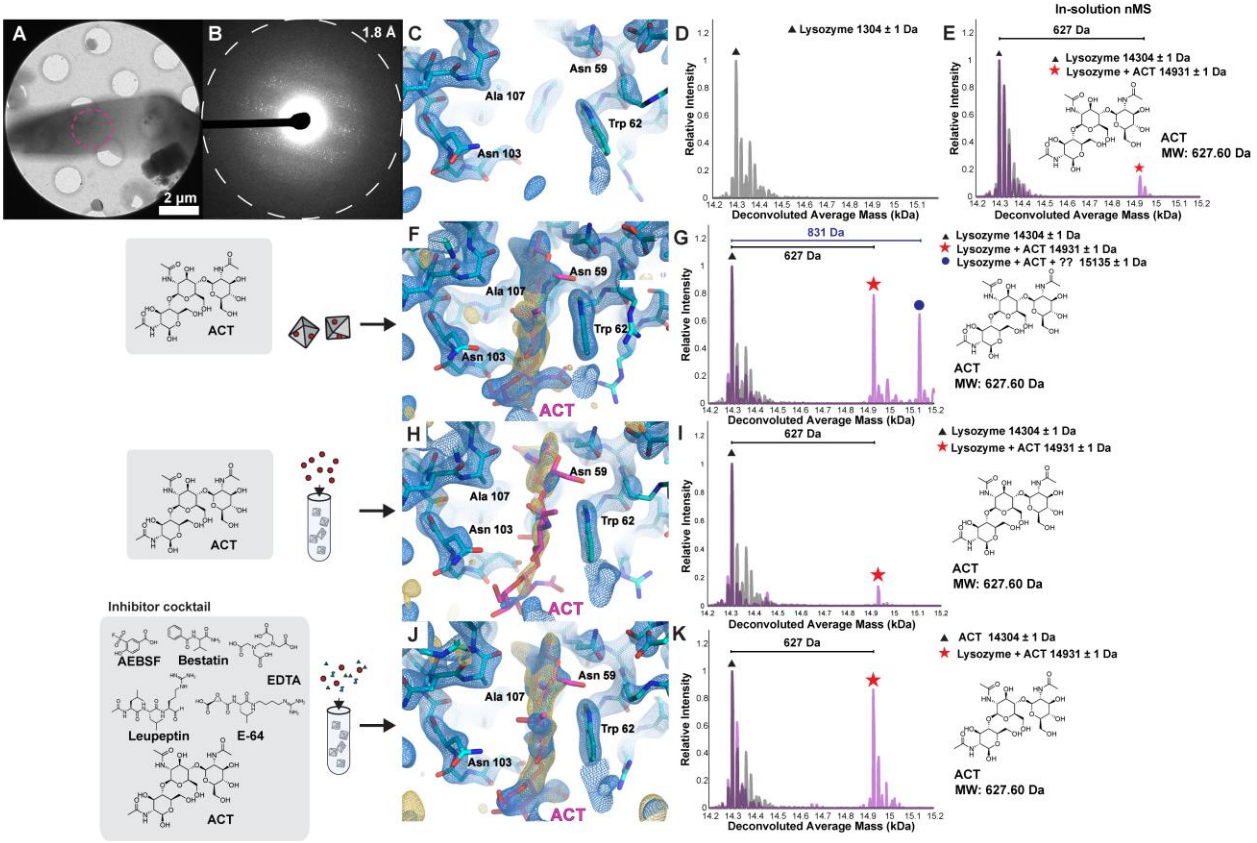

Figure S1. ED-MS of lysozyme with non-covalent ligand N,N',N''-triacetylchitotriose (ACT). TEM image of microcrystals of lysozyme (A) and representative electron diffraction pattern (B). View of the active site from MicroED structure of the apo-form of lysozyme at 2.3 Å resolution (C), and nMS spectrum measured from the on-grid crystals that yielded this structure (D). Representative nMS spectrum of lysozyme mixed with ACT in solution (magenta plot) superimposed on nMS spectrum of apo-form lysozyme without ligand (gray) (E).Active-site view of 2.3 Å MicroED structure of lysozyme co-crystallized with ACT (F), and nMS spectrum of the on-grid lysozyme-ACT cocrystals (magenta plot) superimposed on nMS spectrum of the apo-form lysozyme crystals in panel D (gray) (G). Active-site view of 2.3 Å MicroED structure of lysozyme microcrystals soaked for 10 minutes with pure ACT (H), and nMS spectrum of the on-grid lysozyme-ACT crystal (magenta plot) superimposed on nMS spectrum of the apo-form lysozyme crystals in panel D (gray) (I). Active-site view of 2.4 Å MicroED structure of lysozyme microcrystals soaked for 10 minutes with inhibitor cocktail containing ACT (J), and nMS spectrum of the on-grid cocktail-soaked crystals (magenta plot) superimposed on nMS spectrum of the apo-form lysozyme crystals in panel D (gray) (K).

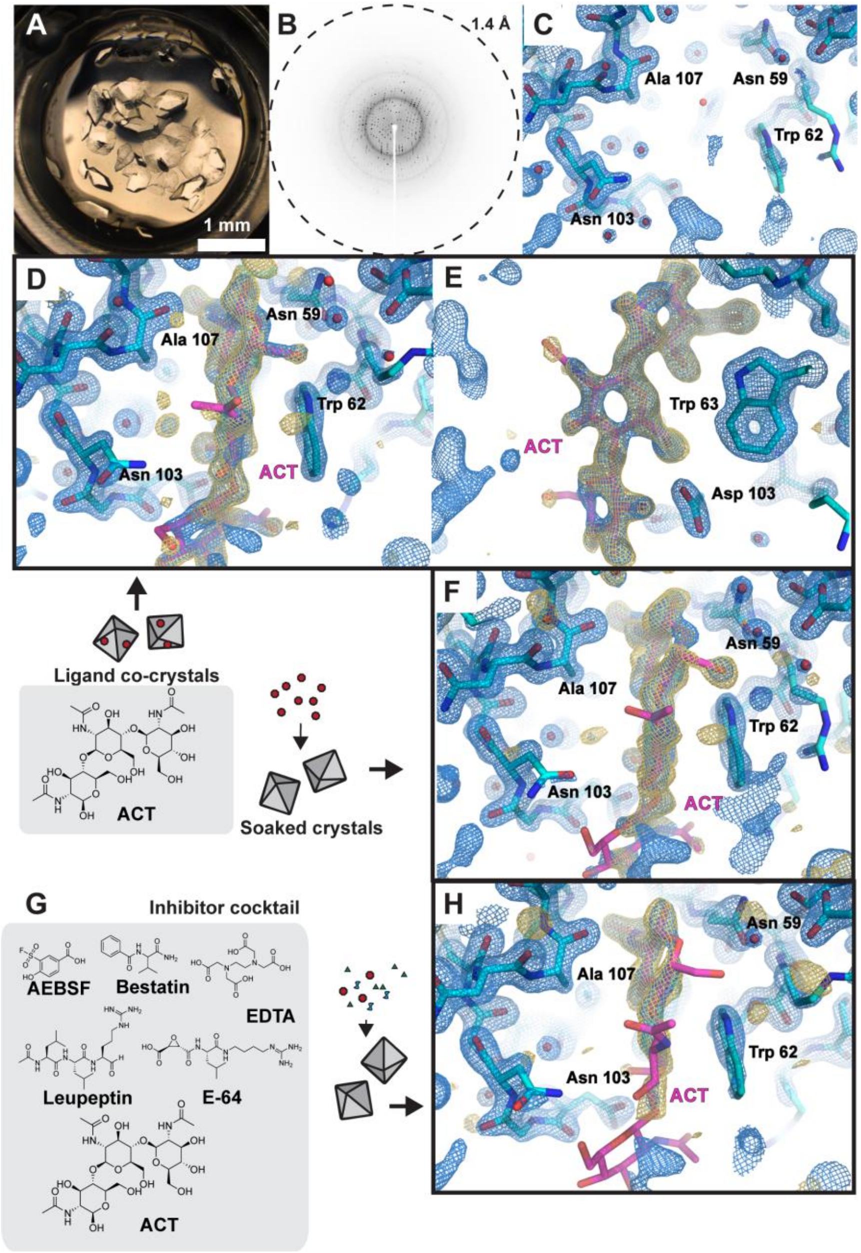

Figure S2. Single-crystal XRD of lysozyme and N,N',N''-triacetylchitotriose (ACT) complexes. Crystals of lysozyme (A) and representative X-ray diffraction pattern (B). View of the active site from single-crystal XRD structures of the apo-form of lysozyme at 1.5 Å resolution (C). Active-site view of single-crystal XRD structure at 1.6 Å resolution of lysozyme co-crystallized with ACT (D), and alternative view of the ligand site from the same structure for (E). Single-crystal XRD structure at 1.4 Å resolution of lysozyme soaked with pure ACT for 20 hours (F). When crystals are instead soaked with an inhibitor cocktail including ACT (G) for 20 hours, the resulting 1.5 Å resolution XRD structure shows comparable results to that from crystals soaked with pure compound (H).

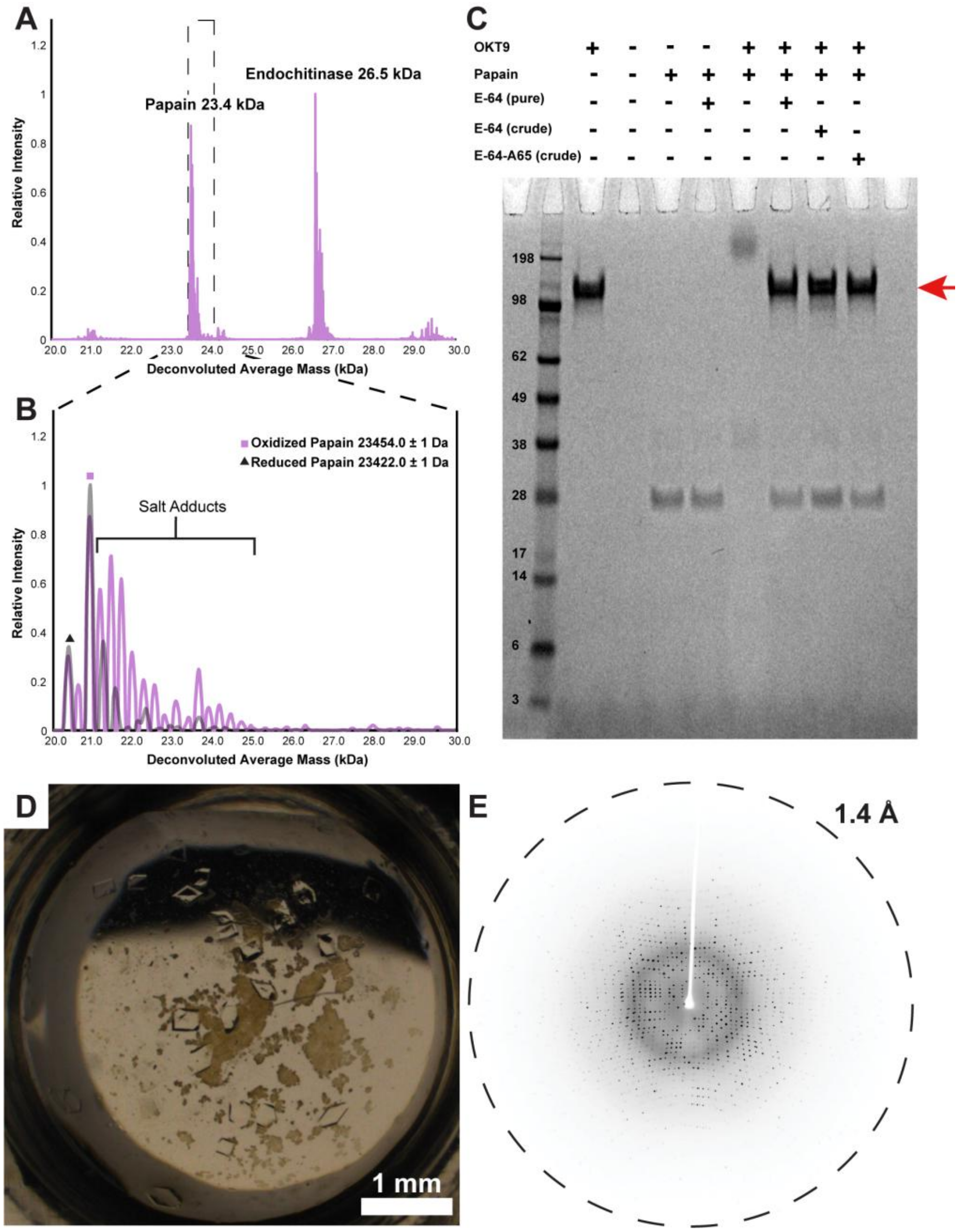

Figure S3. Biochemical characterization of papain. Native mass spectrum of twice-crystallized papain extract used for crystallography experiments (A), with enhanced view of the x-axis region of the spectrum encompassing the masses corresponding to papain present in the sample (spectrum measured from the extract is shown in magenta, overlaid with a spectrum measured from apo-form papain harvested from TEM grids) (B). SDS-PAGE gel indicating activity of papain present in the extract for cleaving model protein OKT9 (150 kDa) in the absence and presence of E-64, as well as crude in-house preparations of E-64 and the analog E-64-A65 from biosynthetic reaction (C). Representative crystals of papain from this sample (D) and single-crystal X-ray diffraction pattern of one such crystal (E).

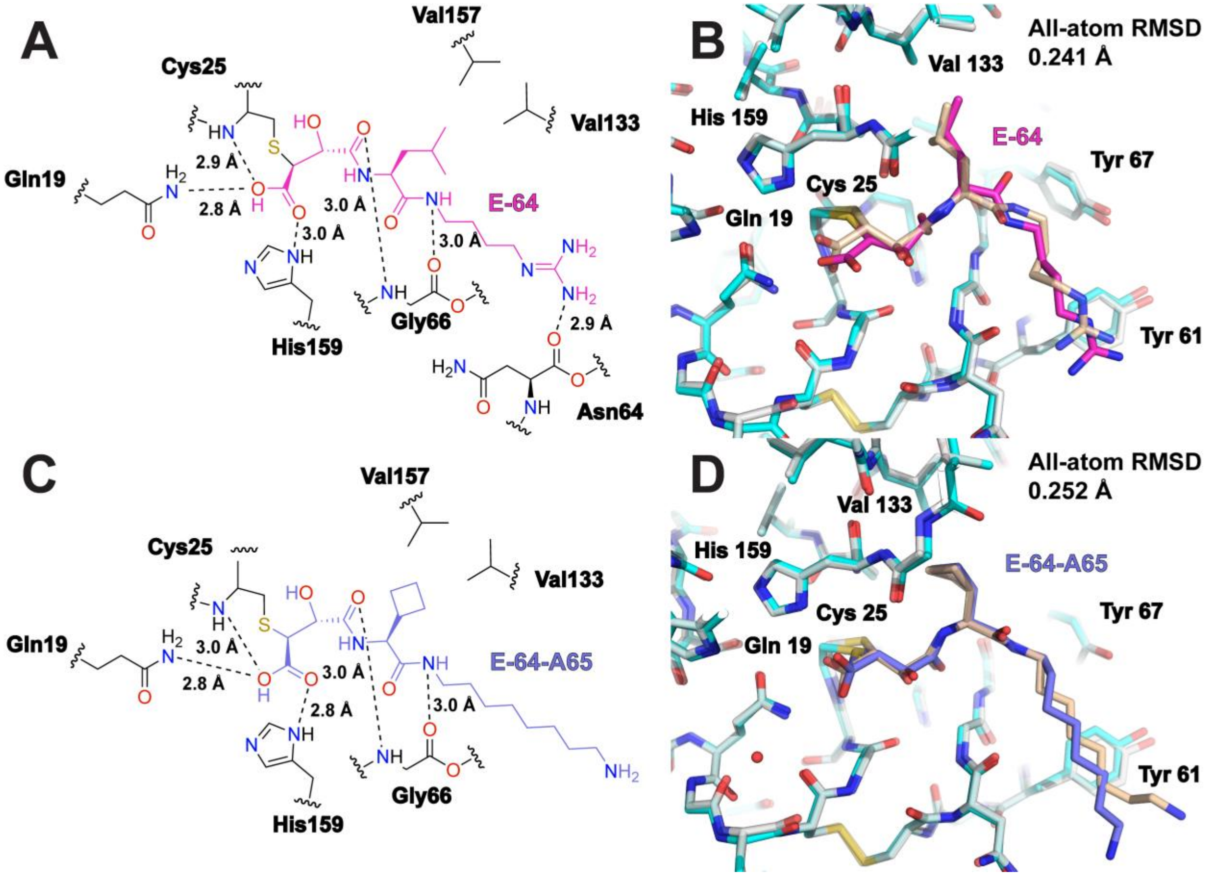

Figure S4. Structure of papain active site complexed with E-64. 2D representation of chemical structure of E-64 bound in papain's active site (A). Overlay of papain-E-64 active site determined by MicroED and the equivalent site determined by single crystal XRD (PDB ID: 9CKT) (B). 2D representation of chemical structure of E-64-A65 bound in papain's active site (C). Overlay of papain-E-64 active site determined by MicroED and the equivalent site determined by single crystal XRD (PDB ID: 9CKT) (D).

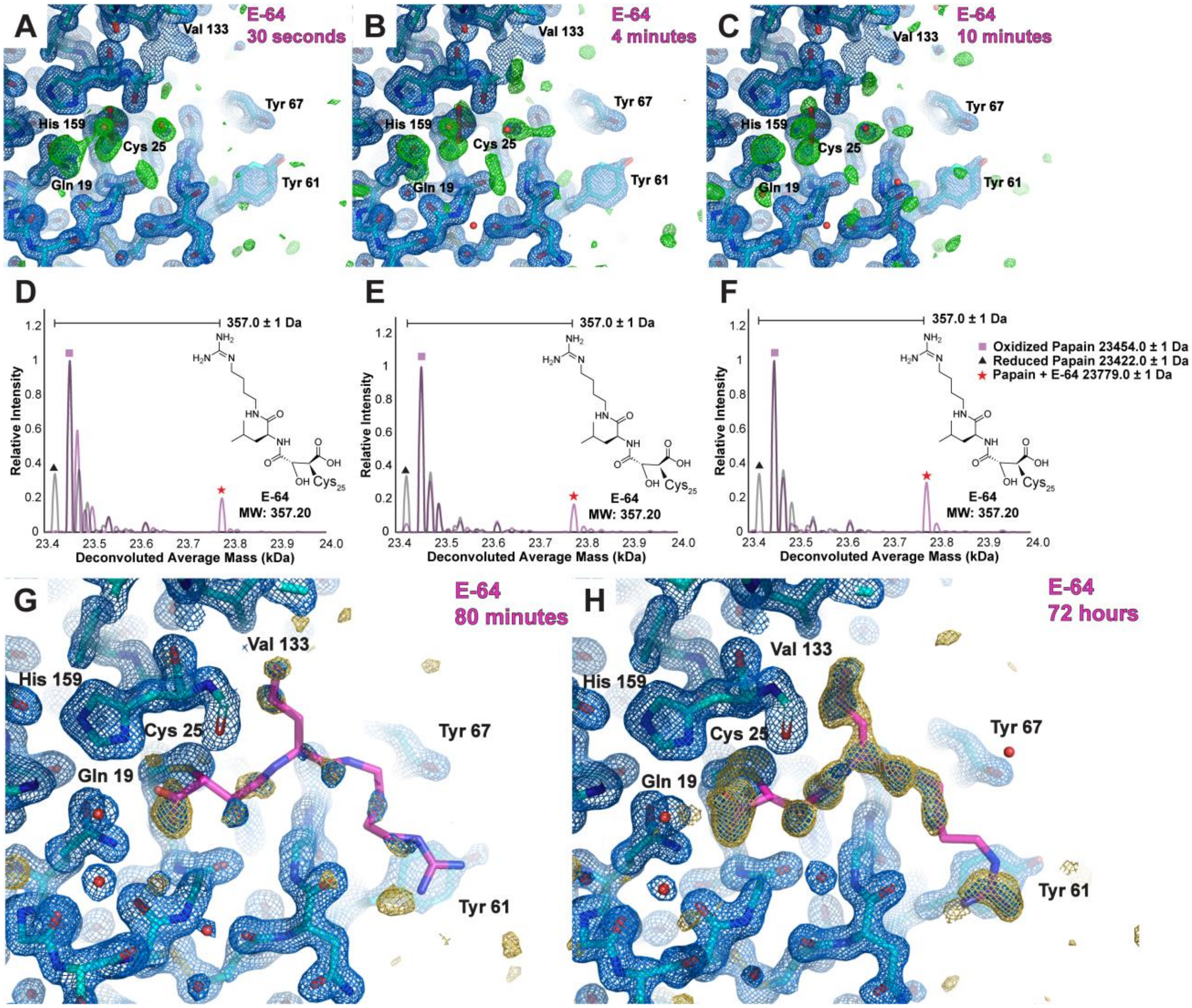

Figure S5. Single crystal XRD of macroscopic papain crystals soaked with E-64 as a function of time. scXRD structures of papain active site at 1.5 Å resolution from crystals frozen following 30 seconds (A), 4 minutes (B), and 10 minutes (C) of soaking with ~2.5 mM E-64. In each case, active site density corresponding to E-64 is not yet visible. Accompanying native mass spectrum measured from a dissolved macroscopic crystal treated in each way (D-F) reveals the presence of some papain-E-64 complex, in addition to the reduced cysteine and cysteine sulfinic acid apo-forms of papain, in each crystal sample, supporting that binding occurs but is diffusion-limited, and that the ligand requires additional time to permeate and bind throughout a macroscopic crystal. For all nMS plots, spectra measured from the soaked crystals (magenta) are compared to that of the apo-protein (gray). scXRD structures of papain active site from crystals frozen following significantly longer times soaking in an equivalent E-64 solution, for 80 minutes (structure at 1.6 Å resolution) (G) and 72 hours (structure at 1.5 Å resolution) (H), where evidence of the ligand becomes more apparent. Blue mesh indicates $2F_o$-F_c map at 1.5σ levels, green mesh indicates F_o-F_c map at 3σ levels at the current stage of refinement the figure at which the structure in the image is displayed, and gold mesh indicates the F_o-F_c map at 3σ levels that was present prior to modeling a ligand which is ultimately satisfied once refinement is complete.

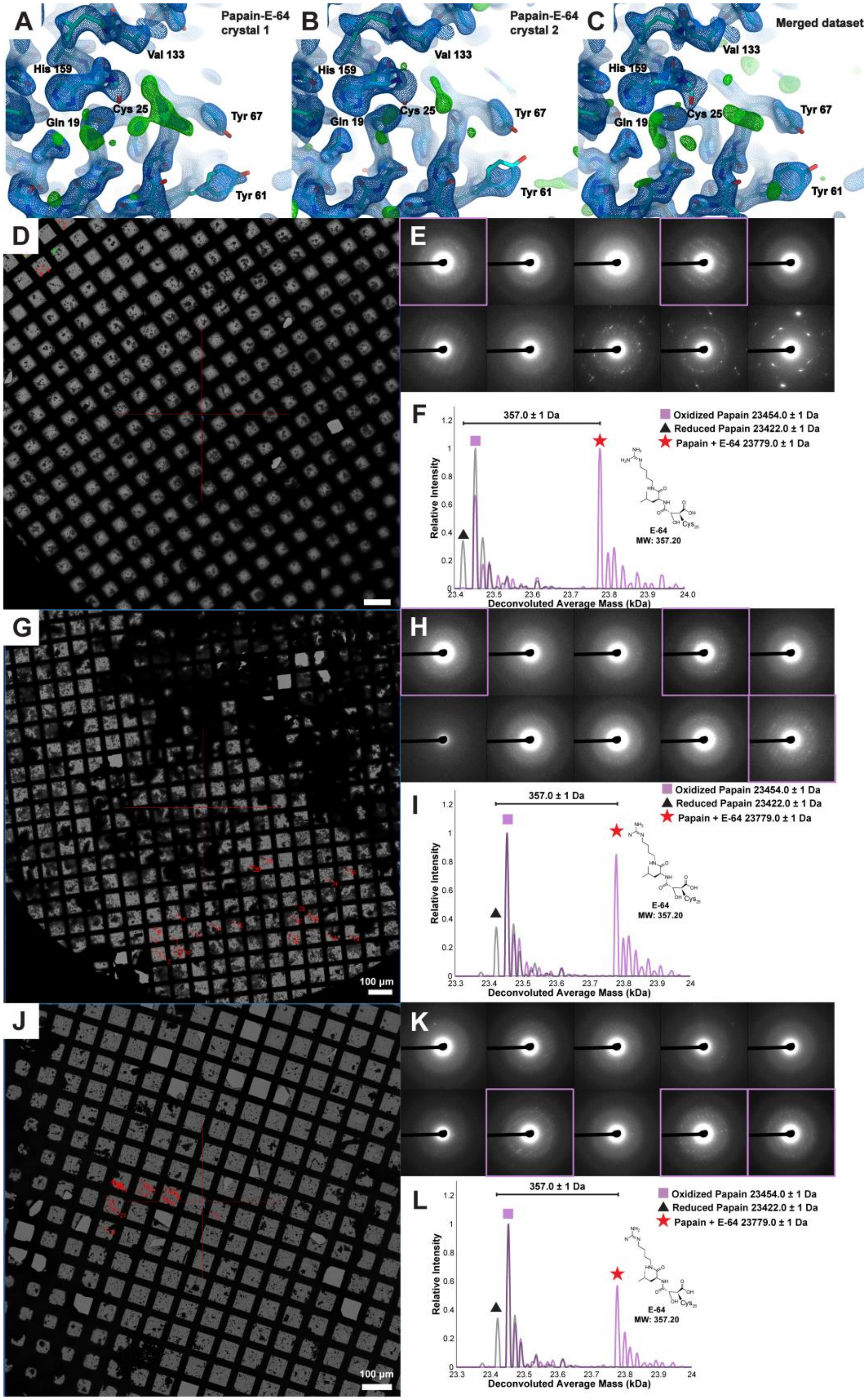
A
Papain-E-64 crystal 1
B
Papain-E-64 crystal 2
C
Merged dataset
His 159
Val 133
Gln 19
Cys 25
Tyr 67
Tyr 61
D
E
F
357.0 ± 1 Da
Oxidized Papain 23454.0 ± 1 Da
Reduced Papain 23422.0 ± 1 Da
Papain + E-64 23779.0 ± 1 Da
Relative Intensity
Deconvoluted Average Mass (kDa)
E-64
MW: 357.20
G
H
I
100 µm
J
K
L

Figure S6. Consistency of ED-MS results between different microcrystals on the same grid, and different TEM grids, for papain complexed with E-64. MicroED structure of the papain active site at 2.3 Å resolution from a single papain microcrystal co-crystallized with E-64 (A), and MicroED structure of the papain active site from a different papain microcrystal on the same TEM grid at 2.2 Å resolution (B) prior to modeling of any ligand in the active site. Density in the structure determined from the first crystal shows distinct evidence of an unmodeled ligand, while the structure determined from the second crystal shows only very weak unsatisfied density in the active site. When reflections from these two crystals are merged to yield a more complete dataset, the resulting structure has only weak residual density in the active site that might indicate a ligand's presence (C). Blue mesh indicates $2F_o$-F_c map at 1.5σ levels and green mesh indicates F_o-F_c map at 3σ levels. Low magnification TEM grid atlas (D), set of representative static diffraction patterns acquired from possibly crystalline particles identified from the atlas, with promising diffraction patterns indicating sites that would be selected for MicroED data collection boxed in lavender (E), and native mass spectrum acquired from the on-grid material after imaging (magenta) compared to the apo-protein (gray) (F), for a sample of papain microcrystals soaked with E-64 for 10 minutes. The same is shown in panels G-I and J-L for different TEM grids prepared in the same fashion.

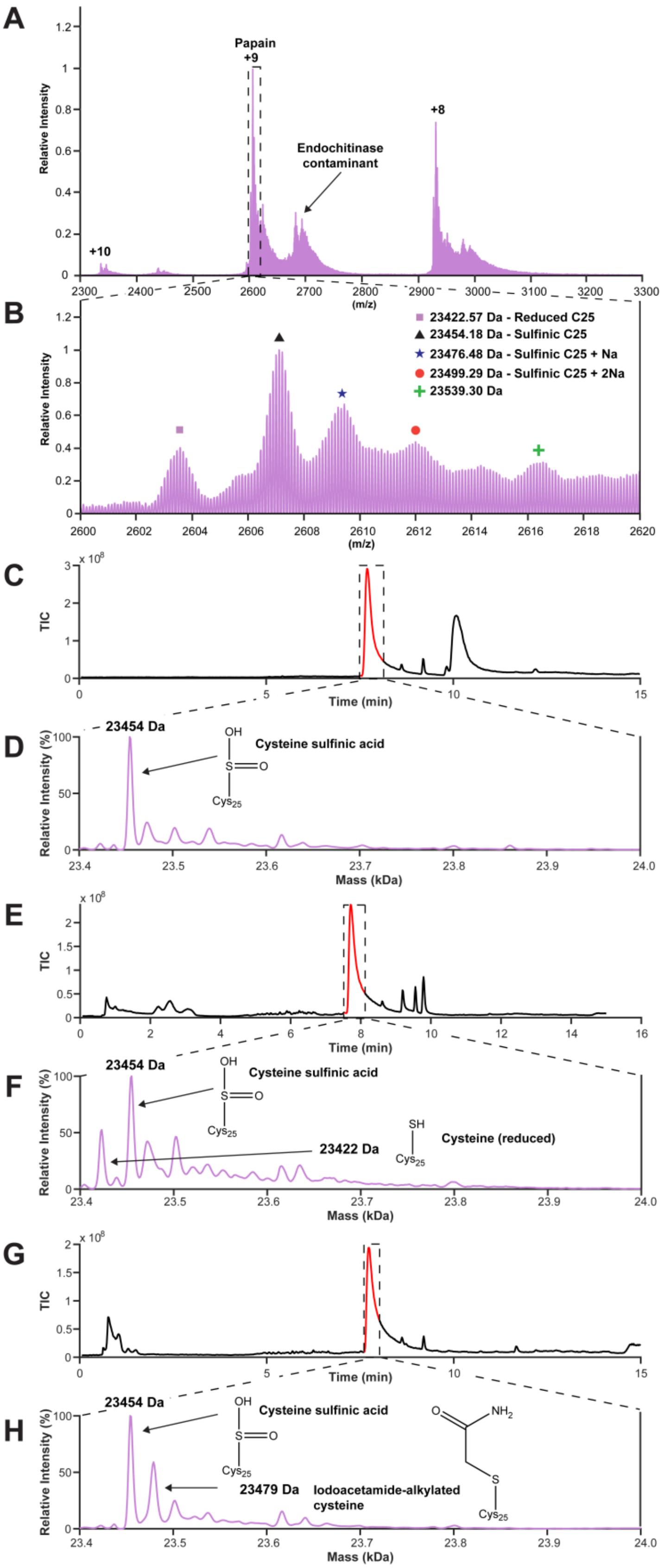
A
Papain
+9
+8
+10
Endochitinase contaminant
Relative Intensity
(m/z)
B
23422.57 Da - Reduced C25
23454.18 Da - Sulfinic C25
23476.48 Da - Sulfinic C25 + Na
23499.29 Da - Sulfinic C25 + 2Na
23539.30 Da
Relative Intensity
(m/z)
C
TIC
Time (min)
D
23454 Da
Cysteine sulfinic acid
Relative Intensity (%)
Mass (kDa)
E
TIC
Time (min)
F
23454 Da
Cysteine sulfinic acid
23422 Da
Cysteine (reduced)
Relative Intensity (%)
Mass (kDa)
G
TIC
Time (min)
H
23454 Da
Cysteine sulfinic acid
23479 Da
Iodoacetamide-alkylated cysteine
Relative Intensity (%)
Mass (kDa)

Figure S7. LCMS analysis of Cys25 oxidation state in papain crystals. High resolution native mass spectrum of twice-crystallized papain extract (A). Enlarged view of 2600 to 2620 m/z identifying average mass of isotopically resolved peaks in the +9 charge state of papain (B). Liquid chromatography plot and mass spectrum of the primary LC peak for papain without reducing agent present (C-D), papain in the presence of an excess of TCEP (E-F) and papain in the presence of an excess TCEP and the free cysteine binder iodoacetamide (G-H). Without reducing agent present, the sulfinic acid form of papain's catalytic cysteine 25 dominates (D), while application of reducing conditions using TCEP is able to recover some of the reduced cysteine form (F). Incubation of papain in reducing conditions with iodoacetamide confirms that the papain-iodoacetamide complex forms, indicated by a 57 Da mass-shifted peak in the mass spectrum relative to reduced papain (H). This indicates that in its reduced form Cys25 is available for binding to covalent ligands.

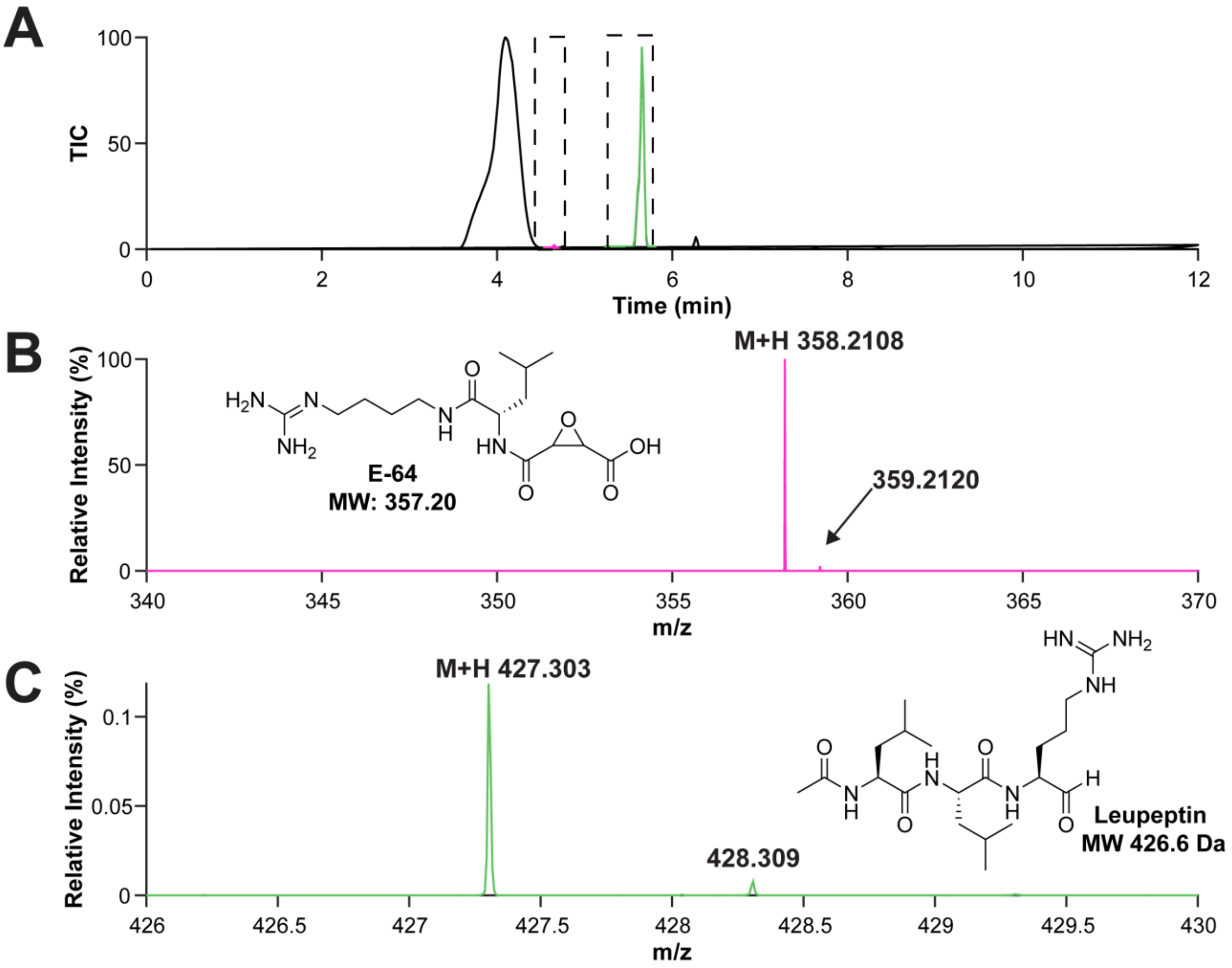

Figure S8. LCMS analysis of commercial protease inhibitor cocktail. Liquid chromatography plot from LCMS of protease inhibitor cocktail, with the elution peak for E-64 highlighted in magenta and elution peak for leupeptin highlighted in lime (A). Mass spectrum of the magenta LC peak in panel A confirming presence of E-64 (B) and mass spectrum of the lime LC peak in panel A confirming presence of leupeptin (C).

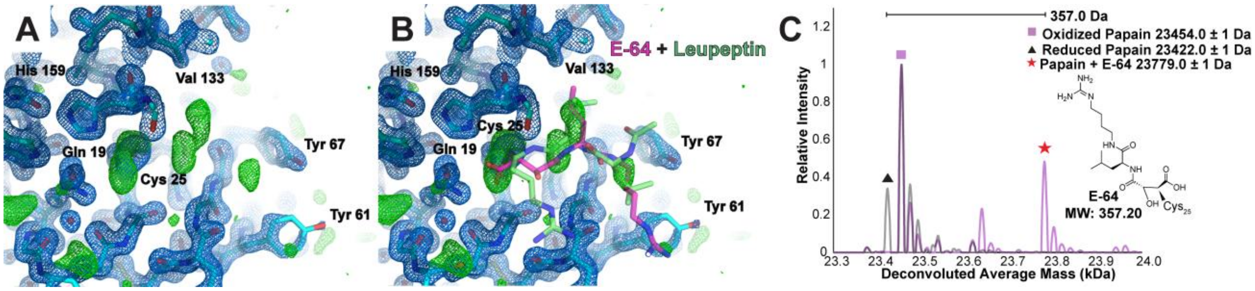

Figure S9. Single crystal XRD structure and nMS spectrum of papain crystals soaked with a commercial protease inhibitor cocktail for 20 hours. scXRD structure of the papain active site at 1.5 Å resolution from crystals frozen after 20 hours of soaking with commercially available protease inhibitor cocktail containing E-64, AEBSF, leupeptin, aprotinin, bestatin, and EDTA at varying relative concentrations. Model superimposed with $2F_o$-F_c map at 1.5σ and F_o-F_c map at 3σ following refinement without any ligand modeled (A), and the same structure overlayed with E-64 (magenta coordinates) and leupeptin (lime coordinates) in the active site (B). Either potential binder satisfies the active site density reasonably well, signaling that even with the improved resolution afforded by XRD compared to MicroED unambiguously distinguishing structurally similar ligands is non-trivial without nMS. nMS spectrum from crystal used for XRD data collection, revealing binding of E-64 to papain (magenta) compared to the apo-protein (gray) (C).

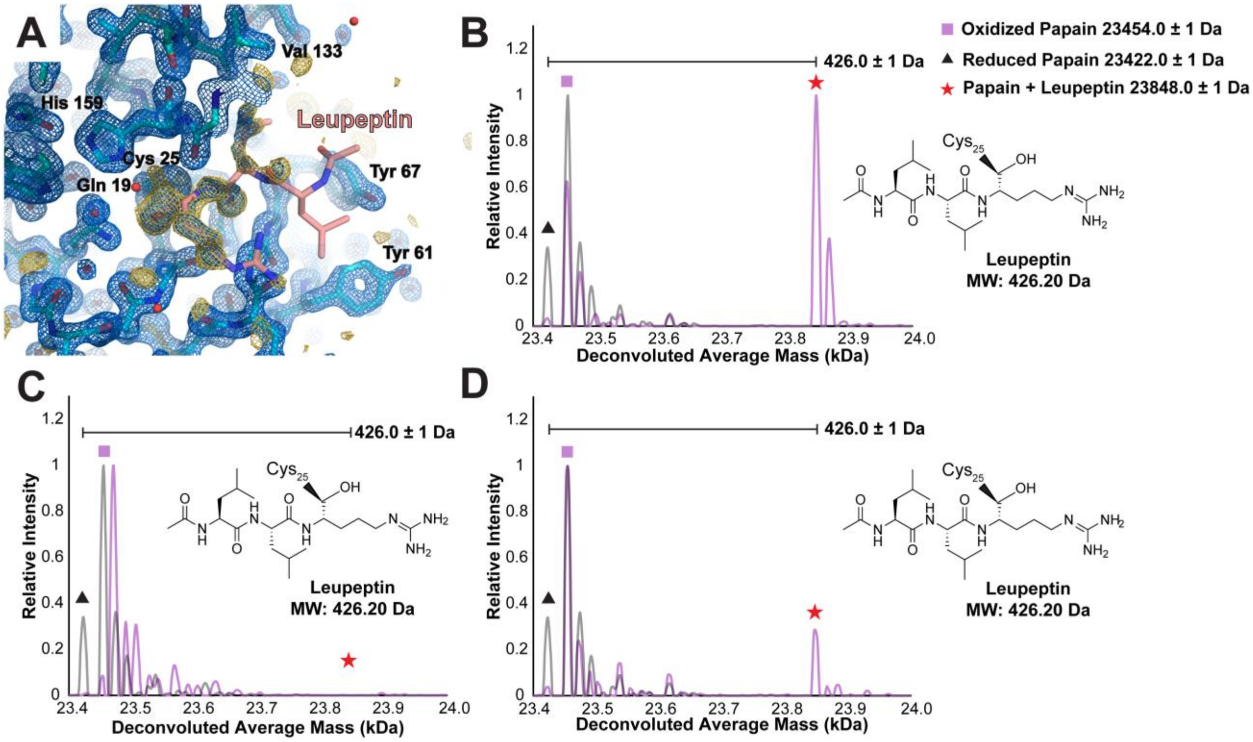

Figure S10. Structures of the papain-leupeptin complex. Single-crystal XRD structure at 1.6 Å resolution of papain co-crystallized with leupeptin with $2F_o$-F_c map in blue at 1.5σ following modeling of leupeptin, alongside gold mesh indicating the F_o-F_c map at 3σ levels that was present prior to modeling a ligand (A). Native mass spectrum of papain mixed with leupeptin in solution (B), macroscopic papain-leupeptin co-crystals suitable for single-crystal X-ray diffraction (C), and papain-leupeptin (micro) cocrystals harvested from a TEM grid (D). In all nMS plots, spectra measured from the sample (magenta) are superimposed on that from the apo-protein (gray).

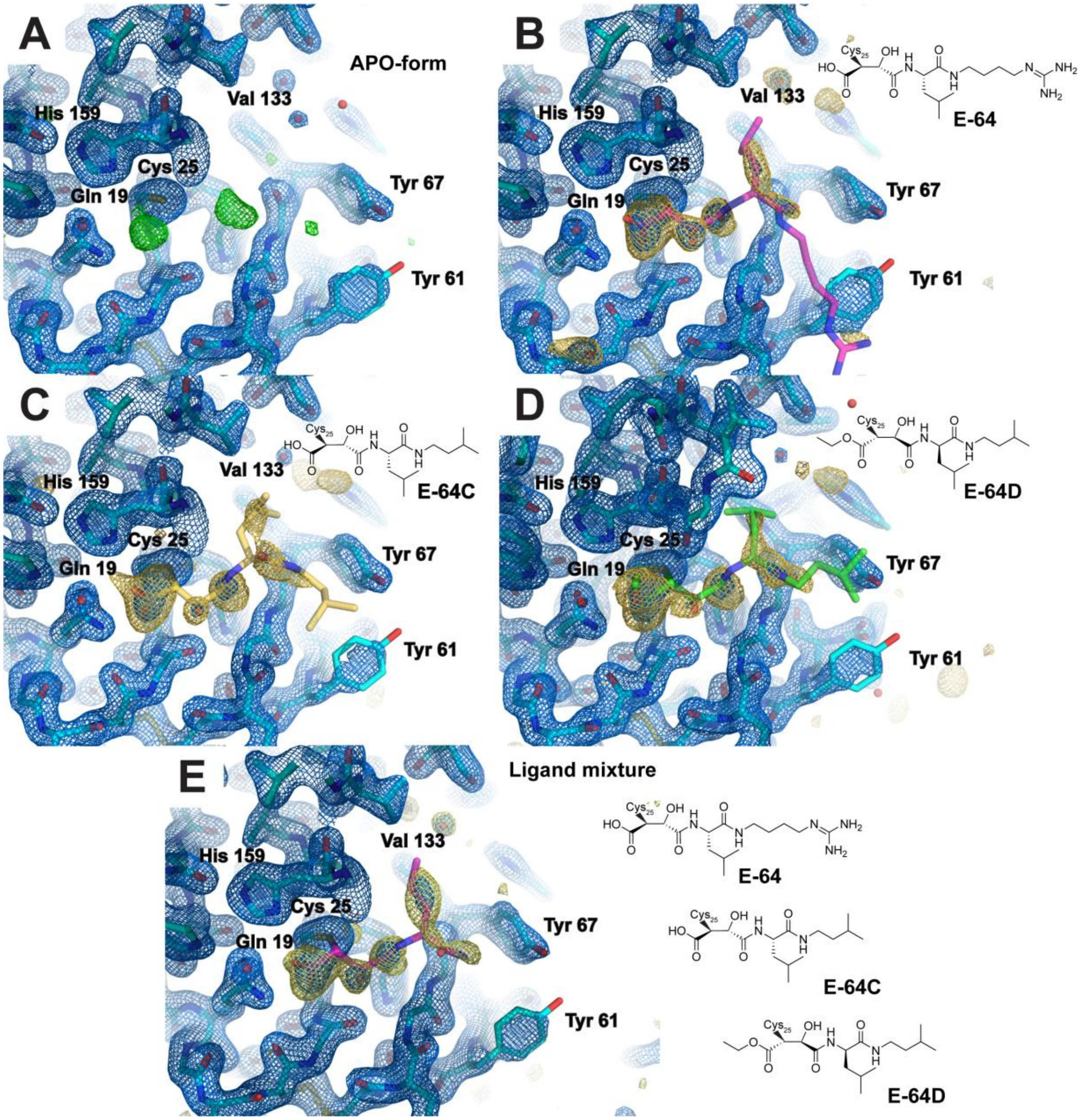

Figure S11. Serial synchrotron XRD structures of papain and high-affinity ligands. Active-site views at 1.8 Å resolution of papain microcrystal structures determined by serial synchrotron X-ray crystallography at beamline ID29 at the European Synchrotron Radiation Facility (ESRF) in apo-form (A), soaked with E-64 (B), soaked with E-64C (C), soaked with E-64D (D), and soaked with an equimolar mixture of all three ligands (E). For the structure determined from the mixture-soaking experiment, the portion of the ligand that is structurally conserved across all three potential ligands can be modeled into the difference density. Blue mesh indicates $2F_o$-F_c map at 1.5σ levels, green mesh indicates F_o-F_c map at 3σ levels at the current stage of refinement the figure at which the structure in the image is displayed, and gold mesh indicates the F_o-F_c map at 3σ levels that was present prior to modeling a ligand which is ultimately satisfied once refinement is complete.

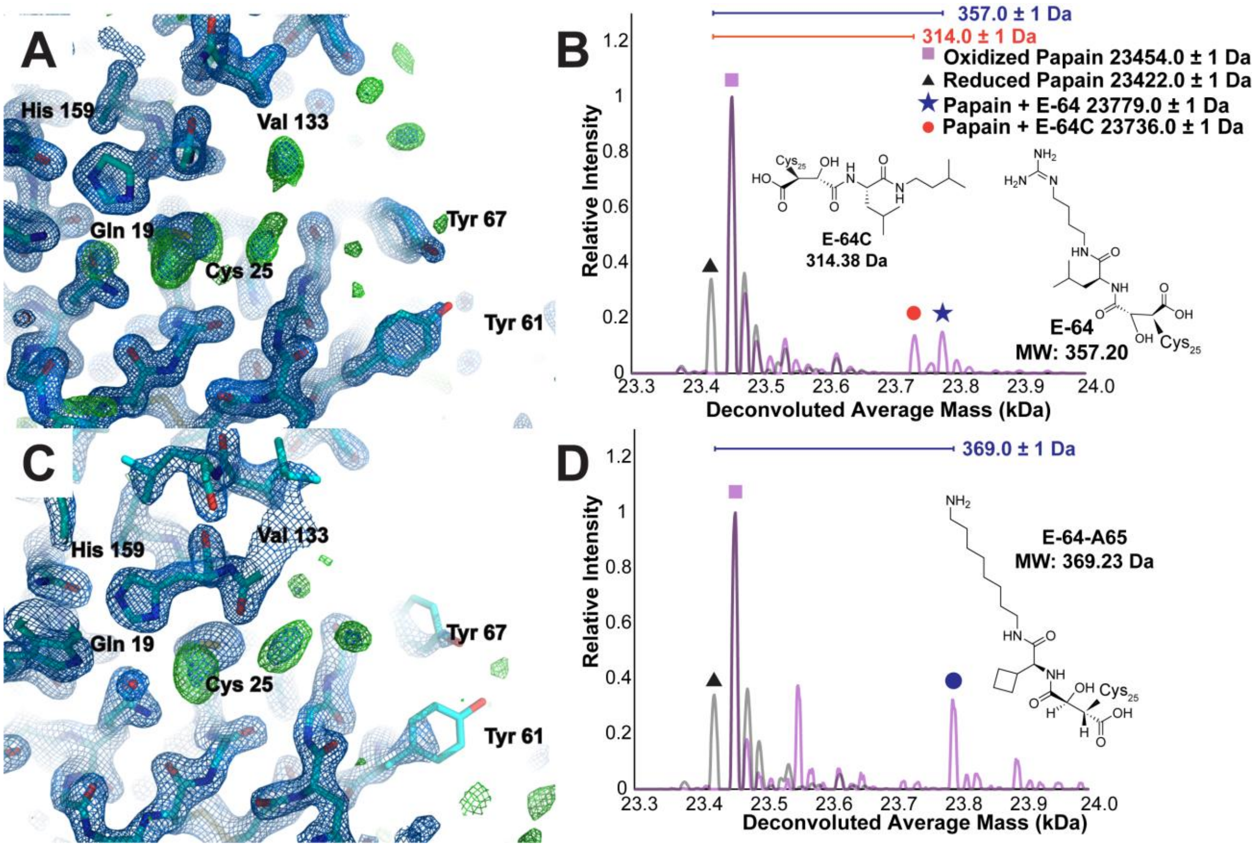

Figure S12. Single crystal XRD structure and nMS spectrum of papain crystals soaked with mixtures of E-64 and E-64 analogs. scXRD structure of the papain active site at 1.4 Å resolution from crystals frozen after 20 hours of soaking with a cocktail of E-64, E-64C, and E-64D at equimolar concentrations. Model superimposed with $2F_o$-F_c map at 1.5σ and F_o-F_c map at 3σ following refinement without any ligand modeled (A). nMS spectrum from crystal used for XRD data collection, revealing some binding of E-64, and E-64C to papain (B). **s**cXRD structure of the papain active site at 2.0 Å resolution from crystals frozen after 20 hours of soaking with a cocktail of biosynthetic E-64 analogs E-64-A65, E315, E371, and E405 at equimolar concentrations. Model superimposed with $2F_o$-F_c map at 1.5σ and F_o-F_c map at 3σ following refinement without any ligand modeled (C). nMS spectrum from crystal used for XRD data collection, revealing binding of E-64-A65 to papain (D). In all nMS plots, spectra measured from the sample (magenta) are superimposed on that from the apo-protein (gray).

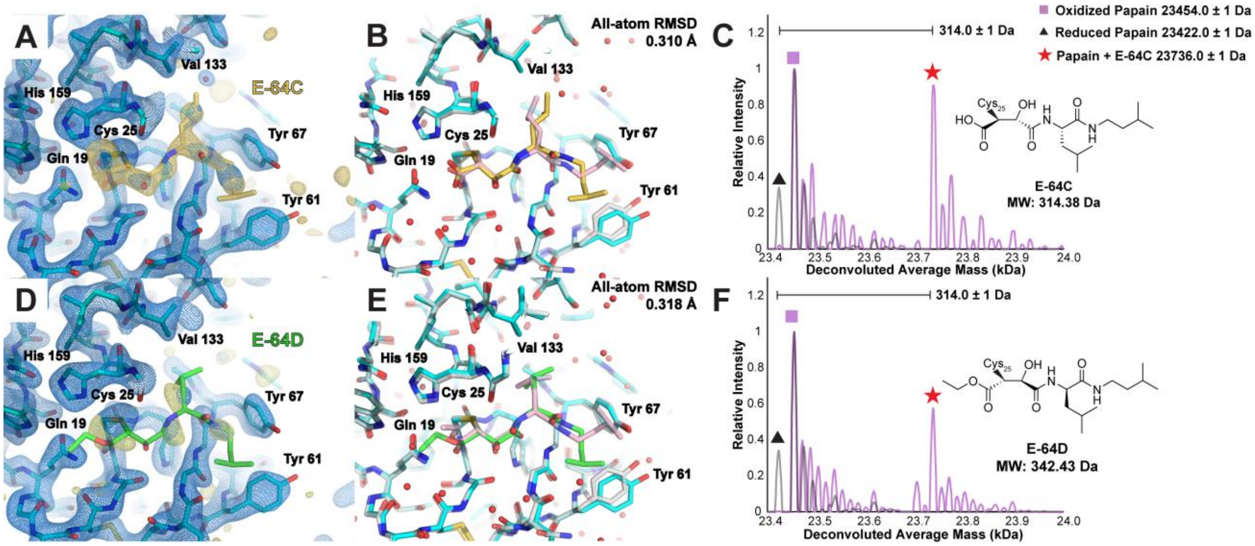

Figure S13. ED-MS of papain-E-64C and papain-E-64D cocrystals. 2.2 Å resolution MicroED structure of papain co-crystallized with E-64C (A), and overlay of this structure with a single crystal XRD structure of the same complex (PDB ID: 9EG7) (B). Native mass spectrum of papain-E-64C co-crystals harvested from TEM grid after MicroED data collection (C). 2.3 Å resolution MicroED structure of papain co-crystallized with E-64D (D), and overlay of this structure with a single crystal XRD structure of the same complex (PDB ID: 9CKW) (E). Native mass spectrum of papain-E-64D co-crystals harvested from TEM grid after MicroED data collection (F). For structure images, the $2F_o$-F_c map is shown in blue at 1.5σ following modeling of the ligand, alongside gold mesh indicating the F_o-F_c map at 3σ levels that was present prior to modeling the ligand. For all nMS plots, spectra measured from the sample (magenta) are superimposed on that from the apo-protein (gray).

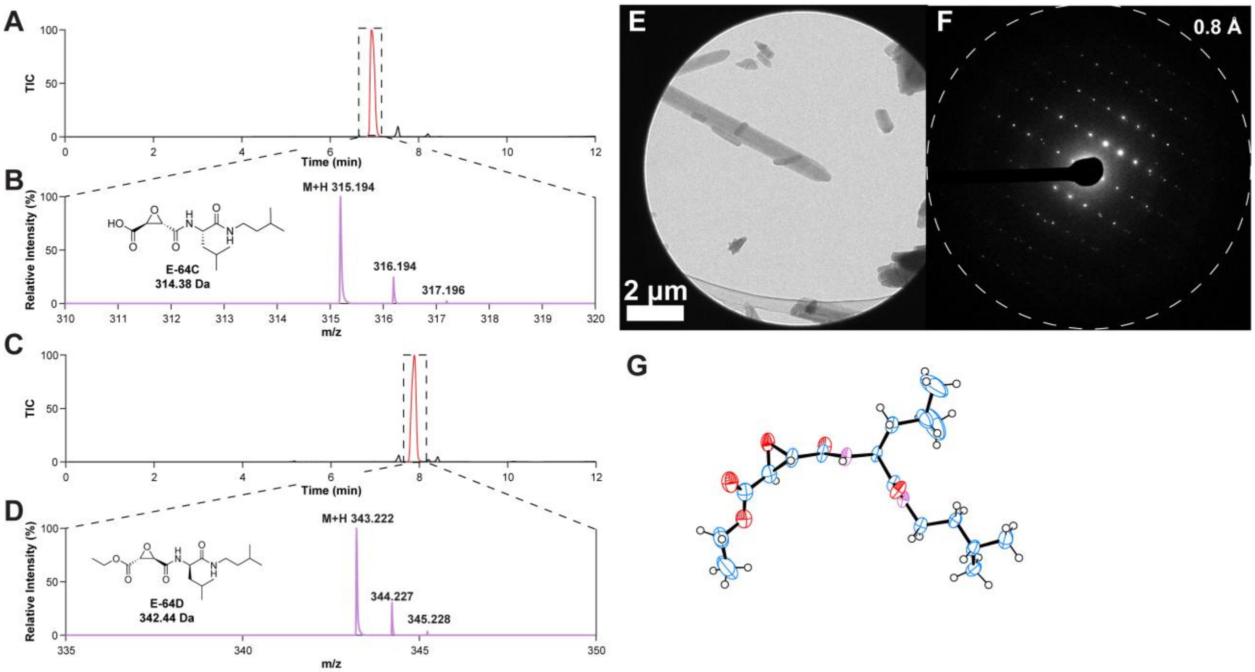

Figure S14. Characterization of E-64D prior to soaking into crystals. LC-MS analysis of E-64C (A-B) and E-64D (C-D) in solution, revealing masses at the expected molecular weights for each. MicroED structure determination of E-64D powder, where nanocrystals (E) yielded single-crystal diffraction to high resolution (F). ORTEP diagram of MicroED structure of E-64 at 0.8 Å resolution, with the terminal ester bound to the epoxide warhead intact (G).

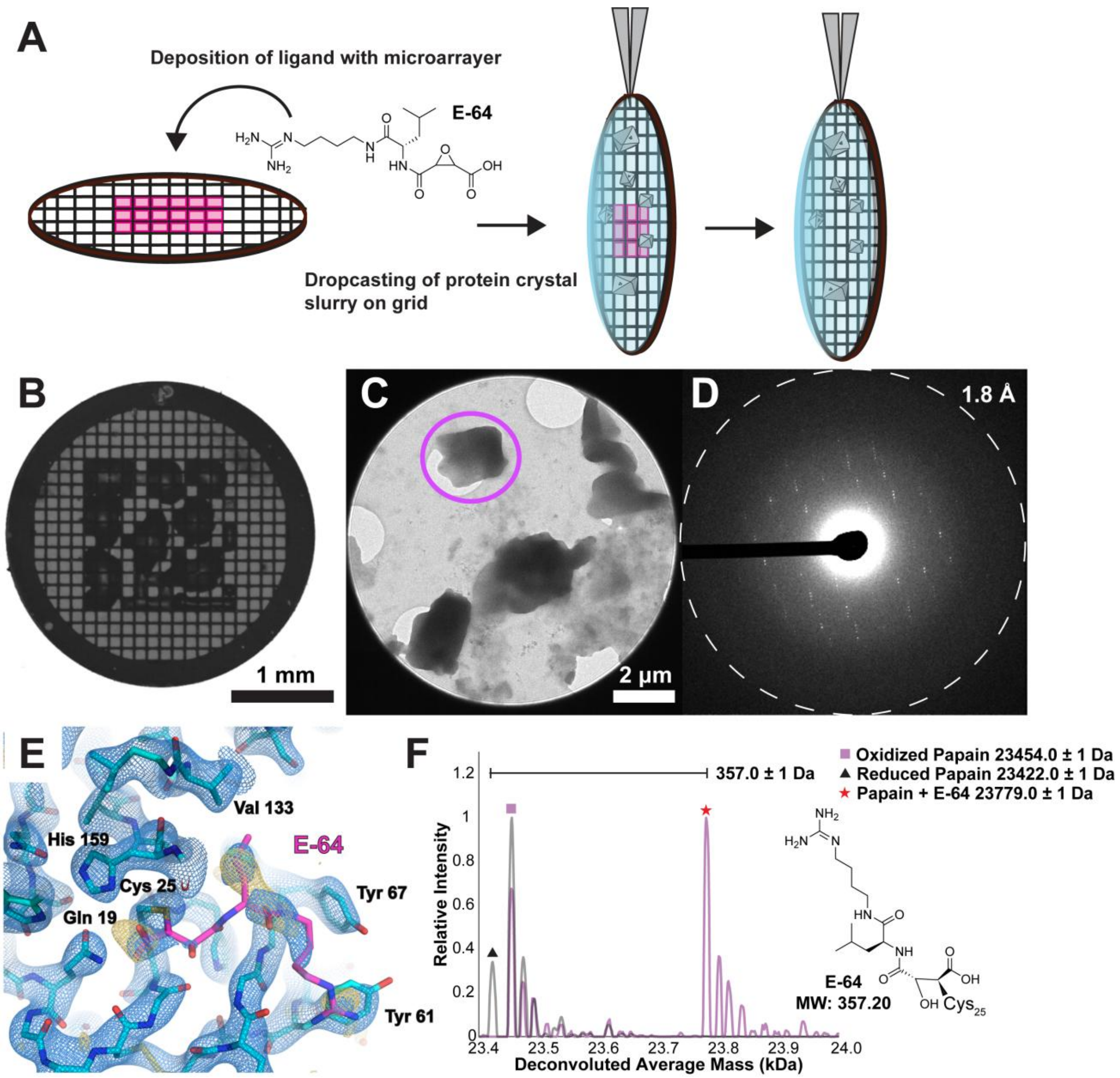

Figure S15. MicroED structure determination of papain microcrystals prepared on ligand-arrayed TEM grids. Schematic of small molecule printing on TEM grids with a microarrayer, after which protein microcrystals were incubated on the printed grid prior to blotting and plunge-freezing (A). Representative light microscopy image of one such grid following arraying with a 26 mM solution of E-64 (B). Representative microcrystal image (C) and electron diffraction pattern (D) of papain crystals prepared on arrayed grids in this fashion. 2.8 Å resolution MicroED structure of the papain active site from microcrystals mixed with arrayed E-64 ligand on grid, with $2F_o$-F_c map in blue at 1.5σ following modeling of E-64 (magenta coordinates), alongside gold mesh indicating the F_o-F_c map at 3σ levels that was present prior to modeling a ligand (E). nMS spectrum of grid-adsorbed material following MicroED collection revealing presence of the papain-E-64 complex (magenta) compared to the apo-protein (gray) (F).

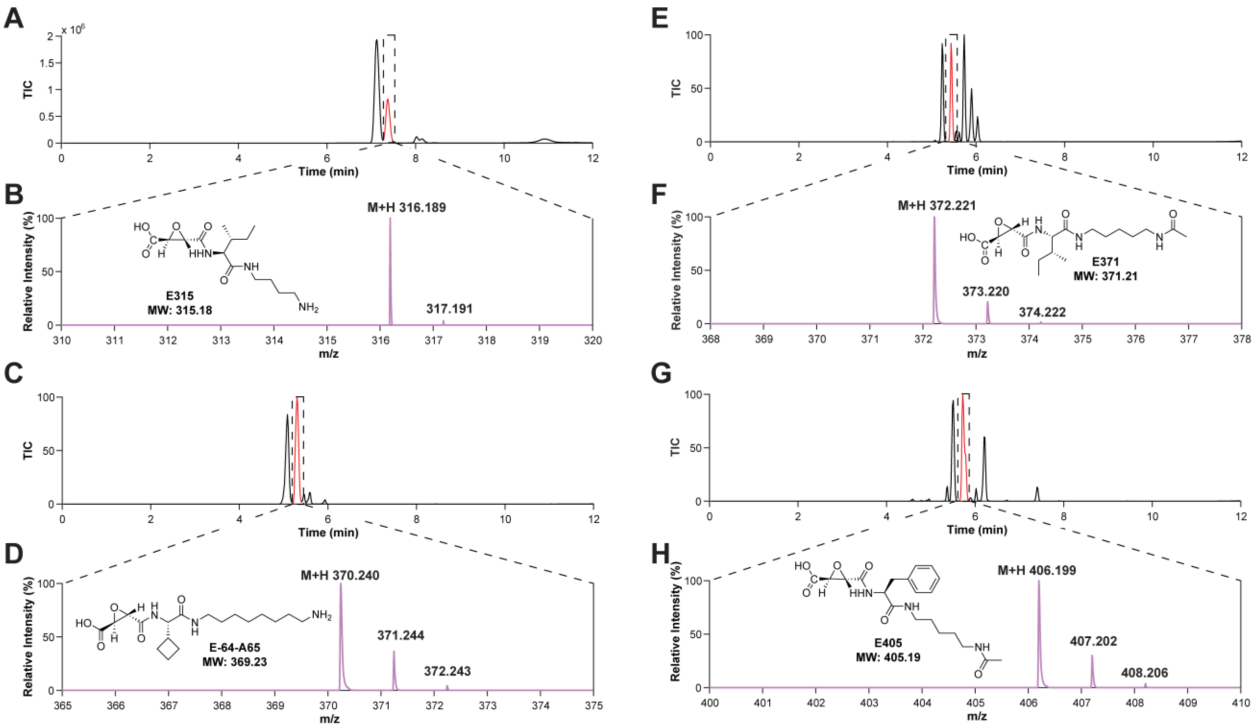

Figure S16. LCMS of biosynthetic E-64 analogs. LC-MS analysis of the biosynthetic E-64 analogs E315 (A-B), E-64-A65 (C-D), E371 (E-F), and E405 (G-H). Panels (A, C, E, G) display chromatograms with highlighted retention times indicating where mass spectra were extracted. Panels (B, D, F, H) present the corresponding mass spectra, confirming the molecular ion peaks for each analog.

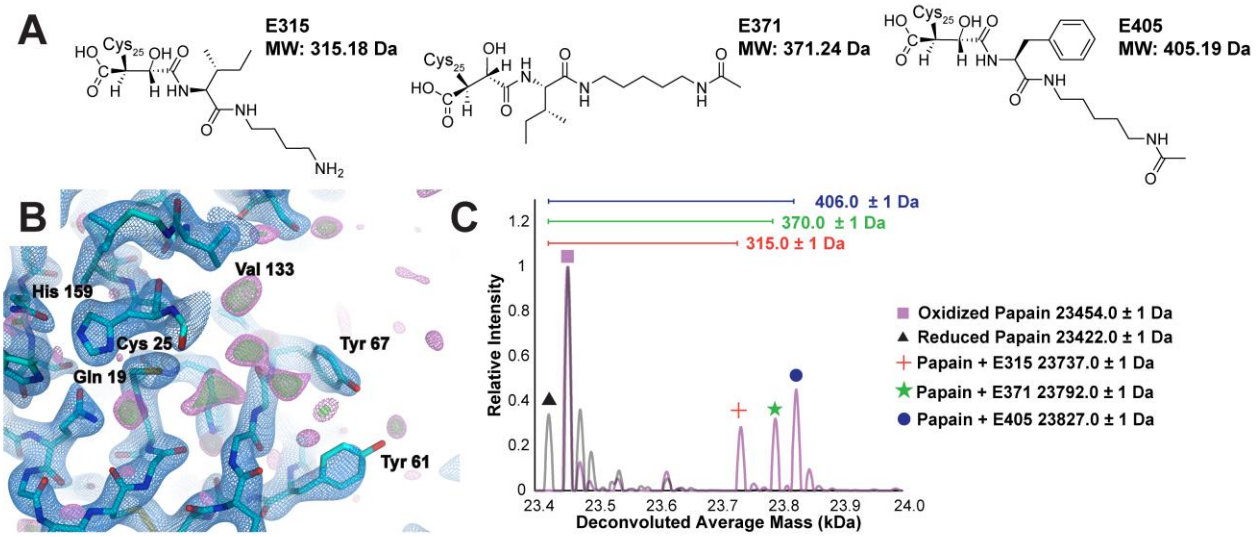

Figure S17. ED-MS analysis of microcrystals soaked with an equimolar mixture of biosynthetic E-64 analogs without E-64-A65. Upon noting by ED-MS that the natural product E-64-A65 bound papain and could be visualized in a MicroED structure, and was likewise detectable as part of a bound species within papain crystals soaked with a mixture of natural product E-64 analogs, a follow-up experiment was performed soaking papain crystals with the same natural product mixture excluding E-64-A65 (A) to identify weaker binders. 2.5 Å resolution MicroED structure of the papain active site soaked with an equimolar mixture of analogs E316, E372, and E406 for 10 minutes revealing weak residual density in the F_o-F_c map within the active site, with 2F_o-F_c map at 1.5σ in blue, F_o-F_c map at 3σ in green, and F_o-F_c map at 2.5σ in magenta (B). Native mass spectrum measured from the same crystals harvested after MicroED data collection (magenta) compared to the apo-protein (gray) (C). While ligand density in the MicroED structure is too weak to easily interpret, nMS indicates that papain-E315, papain-E371, and papain-E405 are all present as bound species.

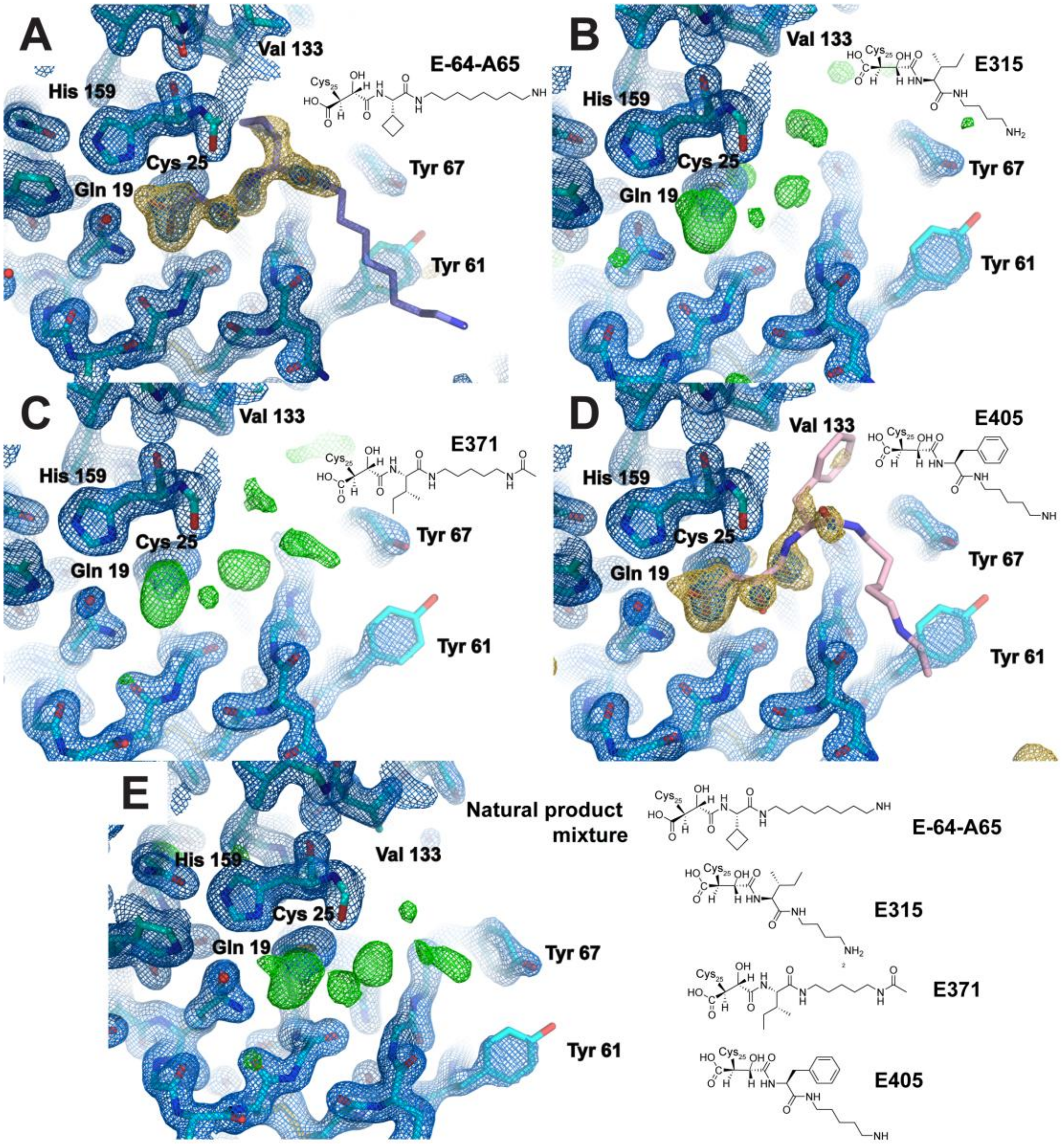

Figure S18. Serial synchrotron XRD structures of papain soaked with natural product inhibitors. Active-site visualizations at 1.8 Å resolution of structures from papain microcrystals determined by serial synchrotron X-ray diffraction at Beamline ID29 at the European Synchrotron Radiation Facility (ESRF). Microcrystals were soaked with either E-64-A65 (A), E315 (B), E371 (C), E405 (D) or an equimolar mixture of all four (E). In cases where the ligand density was clear enough to interpret, as was true for E-64-A65 (panel A, violet coordinates) and E405 (panel D, pink coordinates), the ligand was modeled in the active site and the structure refined. Less well-defined density was measured from crystals soaked with E315 and E371, though the presence of strong peaks unaccounted for by the apo-form structure indicate that some trace binding of these ligands may have occurred. Blue mesh indicates $2F_o$-F_c map at 1.5σ levels, green mesh indicates F_o-F_c map at 3σ levels at the current stage of refinement the figure at which the structure in the image is displayed, and gold mesh indicates the F_o-F_c map at 3σ levels that was present prior to modeling a ligand which is ultimately satisfied once refinement is complete

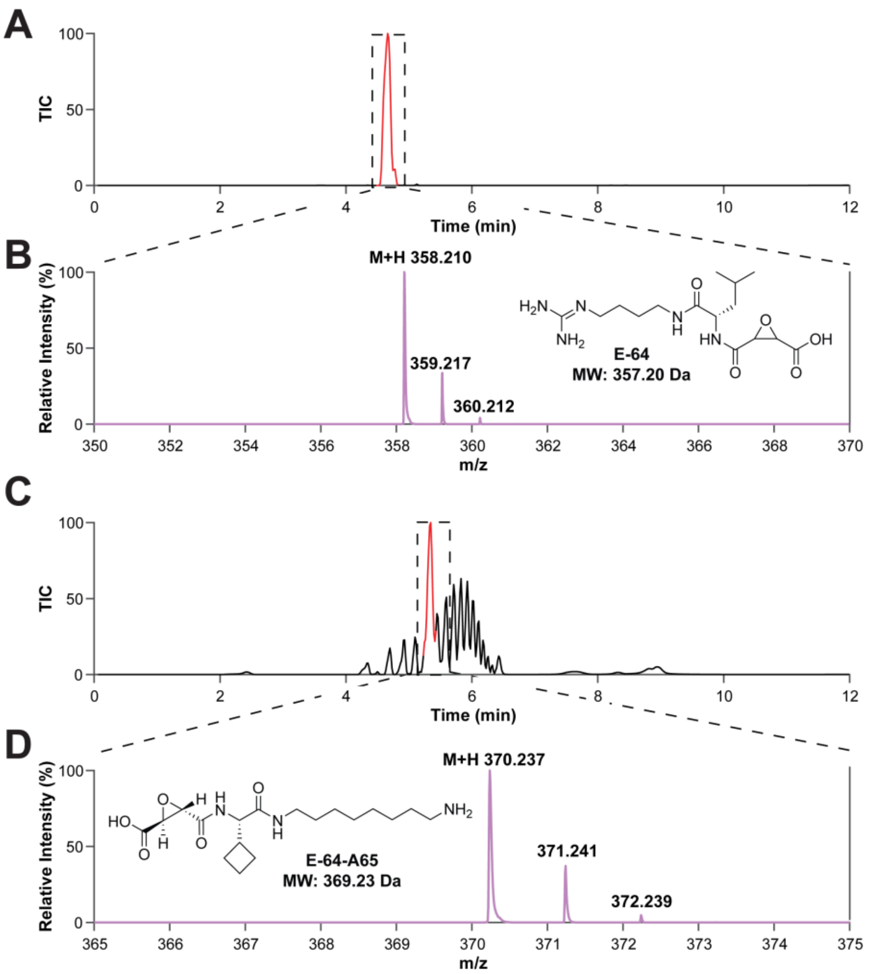

Figure S19. LCMS analysis of crude biosynthetic products: E-64 and E-64-A65. LC-MS analysis of E-64 (A-B) and E-64-A65 (C-D). Panels (A, C) display chromatograms with highlighted retention times indicating where mass spectra were extracted. Panels (B, D) present the corresponding mass spectra, confirming the molecular ion peaks for each compound.

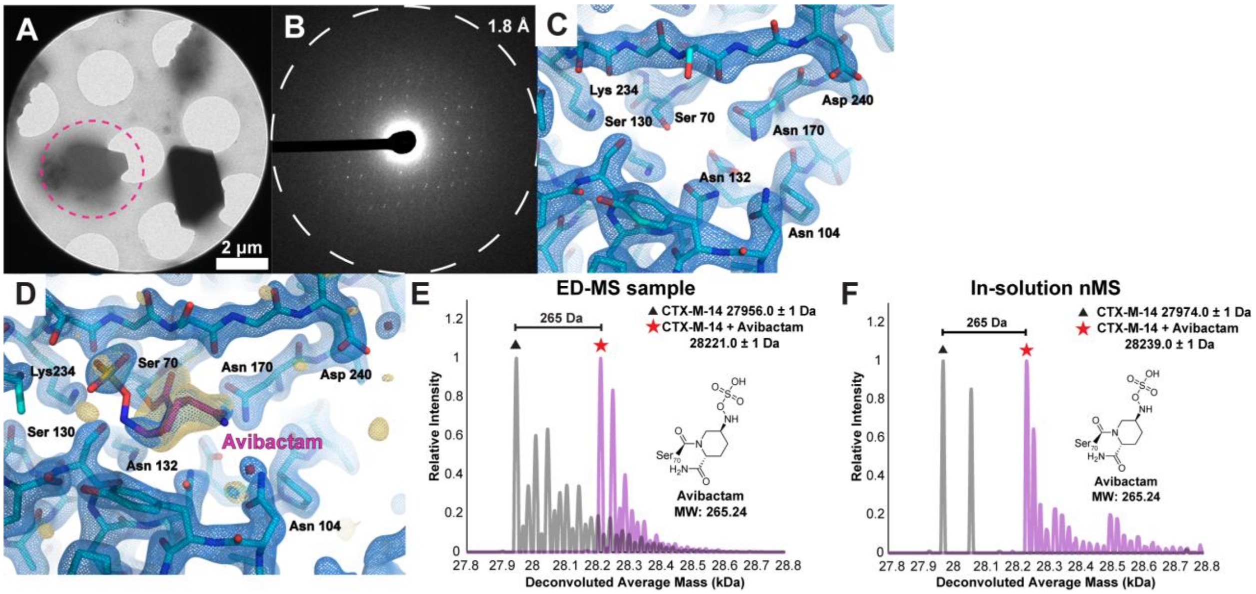

Figure S20. Determination of CTX-M-14 β-lactamase and ligand-complexed structures by MicroED. TEM image of microcrystals of CTX-M-14 β-lactamase (A) and representative electron diffraction pattern (B). View of the active site from MicroED structure of the apo-form of CTX-M-14 β-lactamase at 2.5 Å resolution (C), Active-site view of 2.0 Å MicroED structure of CTX-M-14 β-lactamase co-crystallized with avibactam (D), and nMS spectrum of the on-grid CTX-M-14-avibactam cocrystals (magenta plot) superimposed on nMS spectrum of the apo-form CTX-M-14 crystals that yielded the structure in panel C (gray) (E). Representative nMS spectrum of CTX-M-14 mixed with avibactam in solution (magenta plot) superimposed on nMS spectrum of apo-form CTX-M-14 without ligand (gray) (F).

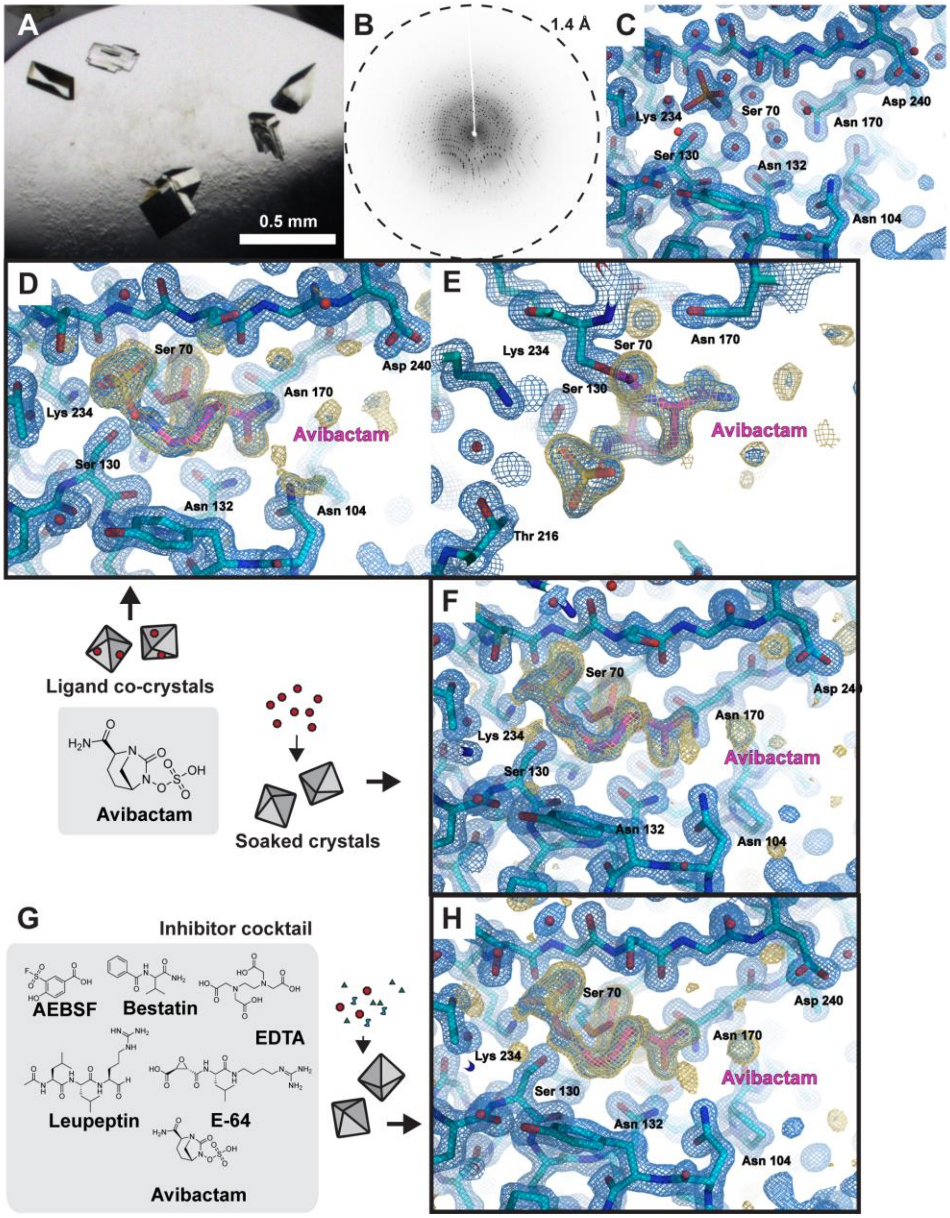

Figure S21. **Single-crystal XRD of CTX-M-14 β-lactamase and avibactam complex.** Crystals of CTX-M-14 β-lactamase (A) and representative X-ray diffraction pattern (B). View of the active site from single-crystal XRD structures of the apo-form of CTX-M-14 β-lactamase at 1.5 Å resolution (C). Active-site view of single-crystal XRD structure at 1.8 Å resolution of CTX-M-14 β-lactamase co-crystallized with avibactam (D), and alternative view of the ligand site from the same structure for clear viewing of the covalent bond between serine 70 and avibactam, and the resolution of avibactam's six-membered ring structure (E). Single-crystal XRD structure at 1.5 Å resolution of CTX-M-14 β-lactamase soaked with pure avibactam for 20 hours (F). When crystals are instead soaked with an inhibitor cocktail including avibactam (G) for 20 hours, the resulting 1.5 Å resolution XRD structure shows comparable results to that from crystals soaked with pure compound (H).

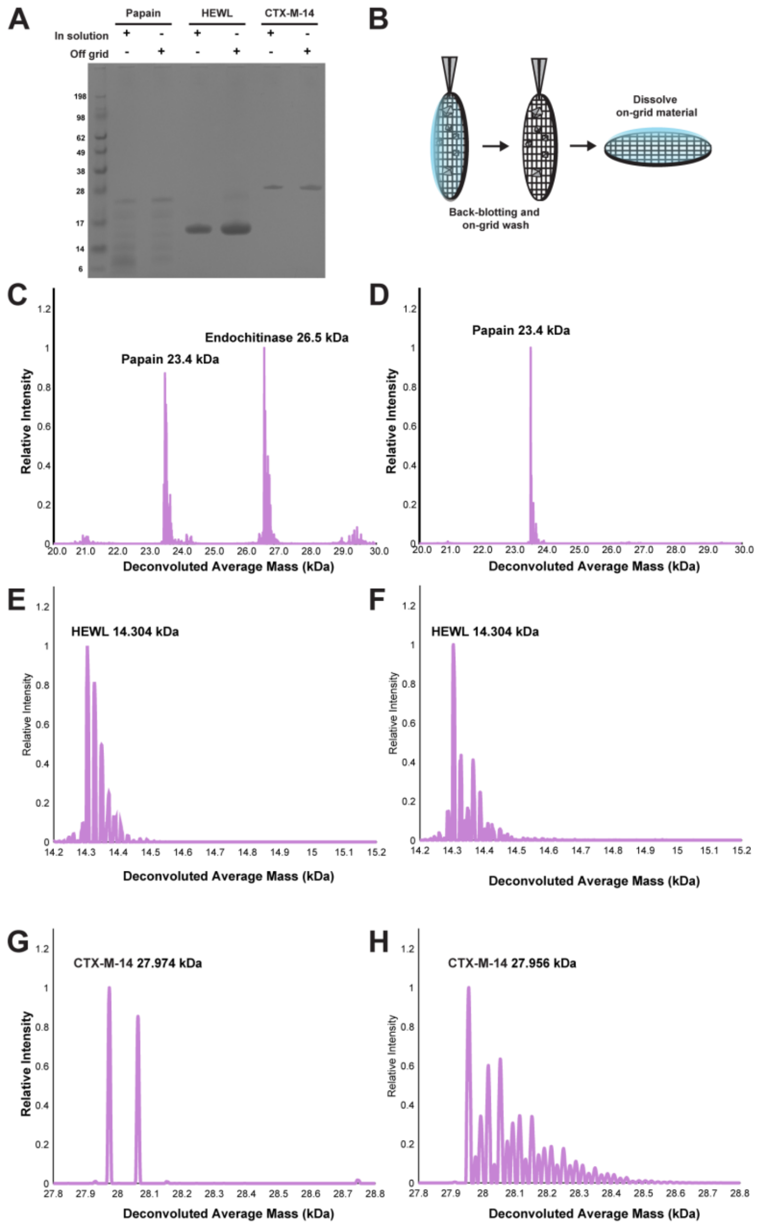

Figure S22. Biochemical verification and native MS analysis of MicroED samples. Image of gel with lanes prepared from papain, hen egg-white lysozyme, and CTX-M-14 β-lactamase, where results from protein in solution, and protein dissolved from crystals harvested from TEM grids used for MicroED are shown side-by-side (A). Schematic of a mock ED-MS sample preparation, where an on-grid wash applied to the sample is successful at removing non-crystalline impurities prior to harvesting, dissolution, and nMS data collection (B). Representative mass spectrum of papain extract in solution (C). Representative mass spectrum of apo-form papain from dissolved crystals harvested from a TEM grid (D). Representative mass spectrum of lysozyme in solution (E). Representative mass spectrum of lysozyme from dissolved crystals harvested from a TEM grid (F). Representative mass spectrum of CTX-M-14 β-lactamase in solution (G). Representative mass spectrum of CTX-M-14 β-lactamase from dissolved crystals harvested from a TEM grid (H).

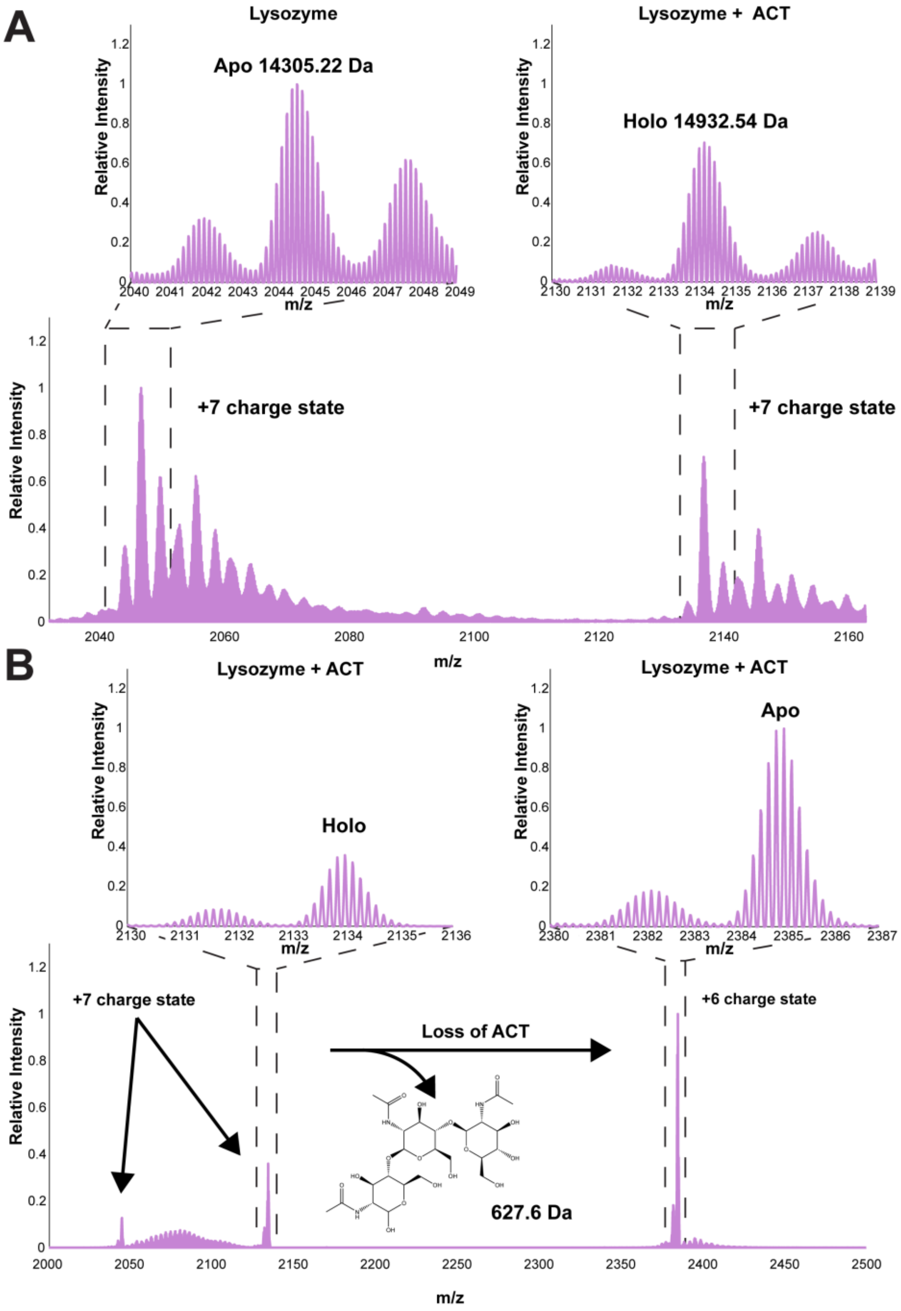

Figure S23 Native mass spectrum and MS/MS spectrum of lysozyme-ACT co-crystals harvested from MicroED grid. High resolution mass spectrum pf lysozyme bound to ACT from crystals on a MicroED grid. Apo (left) and holo (right) isotopically resolved peaks show average masses at the +7 charge state (A). MS/MS mass spectrum of the isolated holo peak acquired with 40CE CID energy (B). The left panel displays the isolated, isotopically resolved holo peak, while the right panel reveals a fragment consistent with the loss of the ACT ligand at the +6 charge state. Additionally, the neutral loss of the ligand can also be seen in the less abundant +7 charge state of apo-form lysozyme (bottom).

CheckCIF/Platon report for MicroED structure of E-64D

Included are responses to A and B level alerts

Alert level A

PLAT029_ALERT_3_A _diffrn_measured_fraction_theta_full value Low . 0.919 Why?

Complete sampling of reflections in MicroED is often limited by accessible tilt range of the TEM, especially in cases where crystals suffer from orientation bias as these thin, plate shaped microcrystals did.

Alert level B

PLAT082_ALERT_2_B High R1 Value .... 0.16 Report
PLAT084_ALERT_3_B High wR2 Value (i.e. > 0.25) .... 0.41 Report

Greater refinement R factors are anticipated in MicroED, when compared to XRD. These mirror the greater R-merge values enountered in data reduction, which are known to be inflated in MicroED by inelastic scattering, dynamical scattering, and other unmodeled aberrations.

PLAT340_ALERT_3_B Low Bond Precision on C-C Bonds .... 0.01954 Ang.

Limited completeness in MicroED data may result in resolution anisotropy of the final electrostatic potential map. This may reduce bond length precision, and might be mitigated by application of further restraints during refinement, at the expense of R-factors.

PLAT911_ALERT_3_B Missing FCF Refl Between Thmin & STh/L= 0.600 158 Report
0 2 0, 0 4 0, 1 5 0, 0 6 0, 2 6 0, 2 7 0,
0 8 0, 0 10 0, 1 10 0, 1 11 0, 0 12 0, 1 12 0,
1 13 0, 0 14 0, 1 14 0, 1 15 0, 0 2 1, 0 3 1,
0 4 1, 0 5 1, 0 6 1, 0 7 1, 0 8 1, 0 9 1,
0 10 1, 0 11 1, 1 11 1, 0 12 1, 1 12 1, 0 13 1,
1 13 1, 0 14 1, 1 14 1, 0 15 1, 1 15 1, 0 3 2,
0 4 2, 0 5 2, 0 6 2, 0 7 2, 0 8 2, 0 9 2,
0 10 2, 0 11 2, 0 12 2, 0 13 2, 1 13 2, 0 14 2,
1 14 2, 0 15 2, 1 15 2, 0 4 3, 0 5 3, 0 6 3,
0 7 3, 0 8 3, 0 9 3, 0 10 3, 0 11 3, 0 12 3,
0 13 3, 0 14 3, 1 14 3, 0 15 3, 1 15 3, 0 0 4,
0 5 4, 0 6 4, 0 7 4, 1 7 4, 0 8 4, 0 9 4,
0 10 4, 0 11 4, 0 12 4, 0 13 4, 0 14 4, 0 15 4,
1 15 4, 0 6 5, 0 7 5, 0 8 5, 0 9 5, 0 10 5,
0 11 5, 0 12 5, 0 13 5, 0 14 5, 0 15 5, 0 0 6,
0 4 6, 0 7 6, 0 8 6, 0 9 6, 0 10 6, 0 11 6,

See response to PLAT082 alert.
